# Supplementary material for: Ultrastrong MXene composite fibers through static-dynamic densification for wireless electronic textiles
Source: Nat Commun. 2025 Dec 9;16:10968. doi: 10.1038/s41467-025-65931-5 (PMC12689709; doi:10.1038/s41467-025-65931-5)
Supplement: Supplementary file 1 — Supplementary Information [file 41467_2025_65931_MOESM1_ESM.pdf]

Supplementary Information

Ultrastrong MXene composite fibers through static-dynamic densification for wireless electronic textiles

Tianzhu Zhou[1,2,3], Jia Yan[4], Can Cao[5], Qiang He[1], Wulong Li[1], Long Chen[1], Chao Wu[6], Yuqi Feng[7], Denvid Lau[7], Qunfeng Cheng[2,3,4,8*], and Lei Wei[1*]

[1]School of Electrical and Electronic Engineering, Nanyang Technological University; 639798, Singapore.

[2]State Key Laboratory of Bioinspired Interfacial Materials Science, School of Nano Science and Technology, Suzhou Institute for Advanced Research, University of Science and Technology of China; Suzhou, Jiangsu 215123, China.

[3]School of Chemistry and Materials Science, University of Science and Technology of China; Hefei, Anhui 230026, China.

[4]School of Chemistry, Key Laboratory of Bio-inspired Smart Interfacial Science and Technology of Ministry of Education, Beihang University; Beijing 100191, China.

[5]School of Materials Science and Engineering, Nanyang Technological University; Singapore 639798, Singapore.

[6]UKCRIC Advanced Infrastructure Materials Laboratory, Department of Civil and Environmental Engineering, Imperial College London; South Kensington, London, SW7 2AZ, UK.

[7]Department of Architecture and Civil Engineering, City University of Hong Kong; Hong Kong 999077, China.

[8]Institute of Energy Materials Science (IEMS), University of Shanghai for Science and Technology; Shanghai 200093, China.

*E-mail: wei.lei@ntu.edu.sg; chengqf@ustc.edu.cn

Contents

Supplementary Methods........3
Supplementary Note 1. Characterization of Ti_3AlC_2 and accordion-like MXene........9
Supplementary Note 2. Characterization of MXene nanosheets........10
Supplementary Note 3. Characterization of CNTs........12
Supplementary Note 4. Liquid crystal properties and rheological properties of spinning dispersion........13
Supplementary Note 5. Interpretation of FTIR and XPS spectra of MXene composite fibers15
Supplementary Note 6. Photo of MXene composite fibers........16
Supplementary Note 7. nano-CT of MXene composites fibers........17
Supplementary Note 8. Porosity and orientation factor of MC fibers fabricated with different lengths of CNTs........18
Supplementary Note 9. SEM images and EDS mapping of MC fibers fabricated with different lengths of CNTs........20
Supplementary Note 10. Mechanical and electrical properties of MC fibers fabricated with different lengths of CNTs........21
Supplementary Note 11. Porosity and orientation factor of MC fibers fabricated with different weight percentages of CNTs........22
Supplementary Note 12. SEM images and EDS mapping of MC fibers fabricated with different weight percentages of CNTs........24
Supplementary Note 13. Characterization of MC fibers fabricated through different diameters of spinning nozzle........26
Supplementary Note 14. FEA simulation and in-situ XRD characterization of MC fiber being heated........29
Supplementary Note 15. Porosity and orientation factor of MCP fibers........31
Supplementary Note 16. EDS mapping of MCP fibers........33
Supplementary Note 17. Mechanical properties of MC fibers fabricated with different weight percentages of CNTs........34
Supplementary Note 18. Mechanical and electrical properties of MCP fibers........35
Supplementary Note 19. Stress-strain curves of loading-unloading cycles for MXene composite fibers........36
Supplementary Note 20. DFT models of four various interfaces........37
Supplementary Note 21. Fracture mechanism of MCP fiber simulated through FEA........38
Supplementary Note 22. Photographs of the smart textiles........40
Supplementary Note 23. Durability of smart textiles........41
Supplementary Note 24. The electromagnetic performance of the textiles at 13.56 MHz......42
Supplementary Note 25. The MVTR of the smart textiles........43
Supplementary Note 26. The performance of the power unit based on smart textiles........44
Supplementary Note 27. Schematic block diagram of the designed chip........45
Supplementary Note 28. The mechanism of long-range, battery-free wireless human health monitoring system........46
Supplementary Note 29. Temperature durability of the wireless communication system.......47
Supplementary Note 30. Battery-free, body-coupled wireless textiles........48
Supplementary Tables........54
Supplementary References........68

Supplementary Methods

1. Fabrication of MXene ($Ti_3C_2T_x$) nanosheet dispersion.

The MXene nanosheet solutions were synthesized using the following procedure: Initially, 2.7 g of Ti_3AlC_2 powders were combined with a solution consisting of 5.7 g of LiF dissolved in 60 mL of 9 M HCl at room temperature. Subsequently, the mixture was stirred at 50 °C for 30 hours to ensure a complete reaction. The resulting accordion-like MXene product underwent thorough washing: three cycles with 9 M HCl solution and then approximately ten cycles with deionized water, each cycle involving 5 minutes of centrifugation at 3,500 rpm (a relative centrifugal force of 1,329×g) using the high-speed refrigerated centrifuge model HC-3016R from Anhui USTC Zonkia Scientific Instruments Co., Ltd. The sediments obtained were dispersed into 150 mL of deionized water with continuous vibration for 15 minutes until the supernatant solution reached a pH of ~7. The solution was then centrifuged at 1,500 rpm (a relative centrifugal force of 244×g) for 30 minutes to obtain the supernatant solution, followed by centrifugation at 3,500 rpm for another 30 minutes to obtain sediments. Finally, these sediments were dispersed in deionized water to prepare the MXene nanosheet solutions with different concentrations.

2. Preparation of carboxylated multiwalled carbon nanotube solution.

0.01 g of a dispersing agent (XFZ20, purchased from Jiangsu Xianfeng Nanomaterials Technology Co., Ltd) was introduced into 50 mL of deionized water and stirred continuously for 1 hour. Following this, 1 g of carboxylated multiwalled carbon nanotubes (CNTs) (five kinds of CNTs: the diameter of 5.6 ± 2.3 nm with the length of ~0.19 μm, ~0.46 μm, ~2.55 μm, ~7.98 μm, and ~13.91 μm) was incorporated into the solution and stirred for 6 hours. The resulting solution containing the CNTs underwent ultrasonic dispersion using an ultrasonic cell disruptor (model SCIENTZ-IID, SCIENTZ Co., Ltd.) operating at 700 W with a pulse mode of 2 seconds on and 2 seconds off for 2 hours in an ice bath. Upon centrifugation of the CNTs dispersion solution at 3,500 rpm for 30 minutes, a supernatant solution containing dispersed nanotubes was obtained.

3. Preparation of MC fibers through wet-spinning.

MXene ($Ti_3C_2T_x$) nanosheets combined with the CNTs with different lengths of ~0.19 μm, ~0.46 μm, ~2.55 μm, ~ 7.98 μm, and ~13.91 μm were utilized in spinning solutions with weight percentages of 1% at a concentration of 40 mg mL^{-1}. These solutions were then extruded through a nozzle with a diameter of 380 μm into a prepared coagulant bath containing absolute

ethyl alcohol. The flow rate was maintained at 2.08×10^{-9} m^{-3} s^{-1} with the inner radius (r) of nozzle of 1.9×10^{-4} m (190 μm), regulated by controlling the speed of the take-up roller with a draw ratio of 3.0. Subsequently, the resulting CNTs filling MXene (MC) fibers were air-dried at room temperature for 24 hours, denoted as MC-0.19, MC-0.46, MC-2.55, MC-7.98, and MC-13.91. Additionally, a series of MC fibers containing both MXene nanosheets and short CNTs were fabricated, with the CNTs accounting for 1 wt%, 2 wt%, 3 wt%, 4 wt%, and 5 wt% of the total fiber weight. These fibers were designated as MC-1%, MC-2%, MC-3%, MC-4%, and MC-5%, respectively. Pure MXene (MX) fibers were also fabricated using the same wet spinning process, albeit with ammonium chloride solution as the coagulant bath.

4. Fabrication of ultrastrong MCP and MCP-V fibers.

The process began with the preparation of a polylactic acid (PLA) hollow column by wrapping PLA with a thickness of approximately 75 μm around a steel rod with a diameter of 6 mm, followed by storage in a vacuum oven at 215 °C for 0.5 hours. Subsequently, ultra-strong CNTs-filled MXene composite fibers with an outer encapsulation layer of PLA were fabricated from MC fibers using a dynamic thermal drawing process. This involved placing the preform in a two-zone heating furnace, with the top and bottom zones heated to 170 °C and 330 °C, respectively, where the central temperature was 210 °C. The preform, along with the PLA hollow column, was then fed into the furnace with various draw-down ratios (τ), while the MC fibers were simultaneously fed into the top of the hollow column. The resulting ultra-compact fibers were collected and denote as MCP. The draw-down ratio (τ) in this context can be defined as:

$$\tau=\sqrt{\frac{v_D}{v_F}} \qquad (1)$$

where v_D is the drawing speed, and v_F is the feeding speed.

To prepare the PLA-$BaTiO_3$ film, 200 g of PLA was dissolved in 200 mL of trichloromethane and stirred for 12 hours, followed by the addition of 100 g of $BaTiO_3$ with an additional 6 hours of stirring to ensure uniform dispersion. The volatile solvent was then removed to obtain the composite film. A hollow thermal drawing tube with an outer diameter of 12 mm was prepared by wrapping the PLA-$BaTiO_3$ film (thickness ~200 μm) around a steel rod with a 6 mm diameter, followed by storage in a vacuum oven at 215 °C for 0.5 hours. Subsequently, MXene composite fibers with an outer encapsulation layer of PLA-$BaTiO_3$ (denoted as MCP_{Ba}) were fabricated from MC fibers through a dynamic thermal drawing process at a τ of 71. The preform

was placed in a two-zone heating furnace, with the top and bottom zones set to 170 °C and 330 °C, respectively, and a central temperature of 210 °C.

Vinyl silicon resin (20:1) and acetoxy silicone resin were combined in a 1:1 mass ratio to form a double-network polymer matrix. $ZnS\text{-}Cu^{2+}$ phosphors were then dispersed into the matrix at a 2:1 mass ratio, mechanically stirred for 15 minutes, and vacuum-defoamed for 5 minutes. Subsequently, the fluorescent dye was added to the phosphor slurry along with the dyeing agent in a 25:1 mass ratio. The resulting luminescent composite slurry was transferred into a conical container with a 0.45 mm aperture. The MCP_{Ba} fiber was passed through the luminescent slurry and the container, then through heating zones at 150 °C, with the rotational speeds of the initial and collecting pulleys controlled at 20 rad min^{-1}. Finally, the fiber, coated with vinyl silicon-acetoxy silicone resin (VSASR) and $ZnS\text{-}Cu^{2+}$ phosphors (denoted as MCP-V), was cured in an 80 °C oven for 1 hour to complete the process.

5. Density functional theory (DFT) for the interfacial interactions.

All calculations were carried out based on density functional theory (DFT) as implemented in the Vienna *ab initio* simulation package (VASP) with exchange-correlation functional of generalized gradient approximation (GGA) of Perdew-Burke-rnzerhof (PBE) method. A grid of 1 × 1 × 1 Monkhorst-Pack k-points was used for the structural relaxation using the method of gamma-centered. The energy cutoff was set to be 450 eV. The convergence criterion for the energy and maximum force for the optimization were set to 10^{-5} eV and 0.05 eV/Å, respectively. Additionally, the charge density difference ($\Delta\rho$) was examined using the VESTA package according to the following equations:

$$\Delta\rho = \Delta\rho_{total} - \Delta\rho_{M} - \Delta\rho_{N} \quad (2)$$

where $\Delta\rho_{total}$ represents the charge of the total system, while $\Delta\rho_{M}$ and $\Delta\rho_{N}$ denote the charges of segments M and N, respectively.

The electron transfer number (*ET*) at both interfaces was determined using the Bader charge analysis method. The adsorption ability with the surface was evaluated by comparing the adsorption energy, the adsorption energy (E_a) is defined as:

$$E_a = E_{adsorb/surf} - E_{surf} - E_{adsorb} \quad (3)$$

where $E_{adsorb/surf}$, E_{surf}, and E_{adsorb} are the calculated total energies of the substrate with adsorbate(s), the clean substrate, and the isolated adsorbate, respectively.

6. Simulation for densifying transverse wrinkles in wet spinning.

The stress behavior of MXene nanosheets with transverse wrinkles was analyzed using finite element analysis under shear stress during wet spinning. MXene nanosheets, each with a thickness of ~1.5 nm, were modeled with a bending angle of 36° using the commercial Abaqus/CAE 2019 software. These MXene nanosheets exhibit an isotropic bulk modulus (E) of ~330 GPa and a Poisson's ratio of 0.30. In the simulations, transverse wrinkles were incorporated on both sides of the nanosheets. Additionally, loading velocity was applied symmetrically to both sides of the MXene nanosheets, and the resulting stress experienced by the MXene nanosheets was determined according to the simulation.

7. Simulation for thermal drawing.

The finite element model was developed using Abaqus/CAE 2019 software to analyze the mechanical behavior of inner MC fibers in the process of thermal drawing. According to experimental findings, during thermal drawing at a central actual temperature of ~210°C, the PLA hollow tube did not make contact with the MC fibers in the hot zone. The study primarily investigated the compressed mechanical behavior of the PLA hollow tube as it contacted the MC fiber and passed through the drawing furnace. For the simulation, a model of the PLA hollow tube was constructed, featuring an inner diameter of 0.102 mm suitable for MC fibers, an outer diameter of 0.202 mm, and a length of 1 mm. This model was used to examine the mechanical response of MC fiber during the thermal drawing process, noting that the MC fibers have a diameter of ~102.0 μm.

The PLA hollow tube possesses an isotropic bulk Young's modulus (E_P) of 0.40 GPa and a Poisson's ratio (υ_P) of 0.35. The inner MC fibers, featuring a porosity of 14.1% when heated during the dynamic thermal drawing process, have an isotropic bulk Young's modulus (E_M) of 1.0 GPa and a Poisson's ratio (υ_M) of 0.30. All mechanical properties of the samples when heated were assessed using a SUNS EUT4103X Tester. In the simulation, fixed boundary conditions were implemented at the top of the MC/PLA model, while a pulling force matching the experimental conditions was applied at the bottom of the PLA hollow tube to analyze the mechanical behavior under varying draw-down ratios during thermal drawing. The models for the inner MC fibers and PLA tube were defined using equations 4 and 5, incorporating isotropic elasticity-plasticity properties.

$$\begin{Bmatrix} \varepsilon_{11} \\ \varepsilon_{22} \\ \varepsilon_{33} \\ \gamma_{12} \\ \gamma_{13} \\ \gamma_{23} \end{Bmatrix} = \begin{bmatrix} 1/E_s & -v_S/E_s & -v_S/E_s & 0 & 0 & 0 \\ -v_S/E_s & 1/E_s & -v_S/E_s & 0 & 0 & 0 \\ -v_S/E_s & -v_S/E_s & 1/E_s & 0 & 0 & 0 \\ 0 & 0 & 0 & 1/G_s & 0 & 0 \\ 0 & 0 & 0 & 0 & 1/G_s & 0 \\ 0 & 0 & 0 & 0 & 0 & 1/G_s \end{bmatrix} \begin{Bmatrix} \sigma_{11} \\ \sigma_{22} \\ \sigma_{33} \\ \sigma_{12} \\ \sigma_{13} \\ \sigma_{23} \end{Bmatrix} \quad (4)$$

$$G_s = \frac{E_s}{2\times(1+\upsilon_s)} \quad (5)$$

where G_s, E_s, and υ_s represent the shear modulus, Young's modulus, and Poisson's ratio of the samples, respectively, including MC fibers and PLA hollow tubes.

8. Conductivity calculation of MXene-based composite fibers.

The electrical conductivity of the fabricated MX, MC, inner MC, and whole composite fibers of MCP and MCP-V fibers was measured employing a Keithley 2700 source meter through the two-point probe method. Silver paste served as a contact point for connecting two ends of the fibers, ensuring conductivity. The conductivity (ρ) of the measured MXene composite fibers was then calculated utilizing an equation (6):

$$\rho = \frac{L}{SR} \quad (6)$$

where L represents the length of the MXene composite fibers, R denotes the electrical resistance, and S signifies the cross-sectional area of each measured fiber obtained from SEM images, while the cross-sectional area of inner MC fiber and whole composite fiber for MCP and MCP-V fibers. Additionally, we employed SEM-FIB to obtain the cross-sections of the fibers, and then used software (Image-Pro Plus) to analyze and determine the cross-sectional area of MXene composite fibers.

9. Estimation of the orientation degree of MXene-based fibers.

The quantification of WAXS/SAXS patterns involved using the scattering vector q, defined as q=4π sinθ/λ, where 2θ is the scattering angle and φ is the azimuthal angle used as coordinates. The orientation of MXene nanosheets within fibers was determined by analyzing the (002) reflection from the WAXS patterns. To quantify the MXene nanosheet orientation degree of fibers, the orientation distribution was converted into a Herman's orientation factor (*f*) as follows:

$$f = \langle \frac{3}{2}\cos^2\varphi - \frac{1}{2} \rangle \quad (7)$$

where the mean-square cosine is calculated by integrating the scattered intensity I (φ) across the azimuthal angle φ:

$$< cos^2\,\varphi > = \frac{\int_0^{\pi} I(\varphi)\, sin\,\varphi\, cos^2\,\varphi d\varphi}{\int_0^{\pi} I(\varphi)\, sin\,\varphi d\varphi} \quad (8)$$

where φ is the azimuthal distribution and the orientation order parameter is determined as follows:

$$f = \int_0^{\pi} \mathrm{I}(\varphi)\left(\frac{3}{2}\cos^2\varphi - \frac{1}{2}\right)\sin(\varphi)\,\mathrm{d}(\varphi) \quad (9)$$

Additionally, the intensity is normalized based on

$$\int_0^{\pi} \mathrm{I}(\varphi)\sin(\varphi)\,\mathrm{d}(\varphi)=1 \tag{10}$$

10. Calculation of the porosity of MXene-based fibers.

The porosity (γ) of the MXene-based fibers was evaluated according to the structural parameters by equations (11) and (12):

$$\gamma\ (\%)=\left(1-\frac{\rho_{\mathrm{M}}d_{002}}{\rho_{\mathrm{TM}}d_{\mathrm{TM}}}\right)\times 100 \tag{11}$$

$$\rho_{\mathrm{M}}=\frac{m_{\mathrm{f}}-S_{\mathrm{P}}L\rho_{P}}{S_{\mathrm{M}}L} \tag{12}$$

where ρ_{M} denotes the density of MX, MC, and the inner MC fibers within MCP and MCP-C fibers, while d_{002} presents the d-spacing (002) of the fibers for MXene nanosheets. Furthermore, ρ_{TM} corresponds to the theoretical value of Ti_3C_2 density with 5.2 g cm^{-3},[18] and d_{TM} is the d-spacing of Ti_3C_2 crystals of 1.02 nm. Furthermore, m_{f} is the total mass of fibers, while S_{P} represents the area of the outsider layer of PLA, as well as S_{M}, is the area of MX, MC, and the inner MC fibers within MCP and MCP-C fibers, respectively. Additionally, ρ_{P} is the density of the PLA polymer (1.26 g cm^{-3}), and L stands for the length of the MXene composite fibers.

11. Preparation of the smart textiles.

Various types of spiral inductors were designed using the ANSYS HFSS (High Frequency Structural Simulator) module from ANSYS Electronics 2017. Following the electromagnetic simulations, the designs were converted into stitch patterns using the commercial software PE-DESIGN 11 from Brother Co., Ltd. These patterns were then executed on a digital embroidery machine (model NV180, Brother) using MCP fiber. The machine features a digital control area of 10 cm×10 cm, with manual control options available for larger areas. To ensure both mechanical and electrical connectivity, flexible printed circuit boards (F-PCB) were soldered to the ends of the spiral inductors incorporated into the MCP fiber-based textiles.

Supplementary Note 1. Characterization of Ti_3AlC_2 and accordion-like MXene

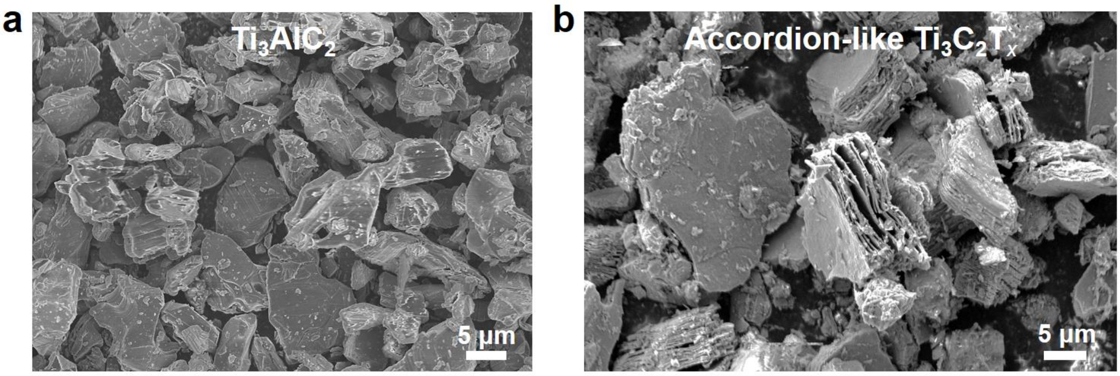

Supplementary Figure 1 | SEM images of Ti_3AlC_2 (**a**) and accordion-like $Ti_3C_2T_x$ (MXene) (**b**).

Supplementary Note 2. Characterization of MXene nanosheets

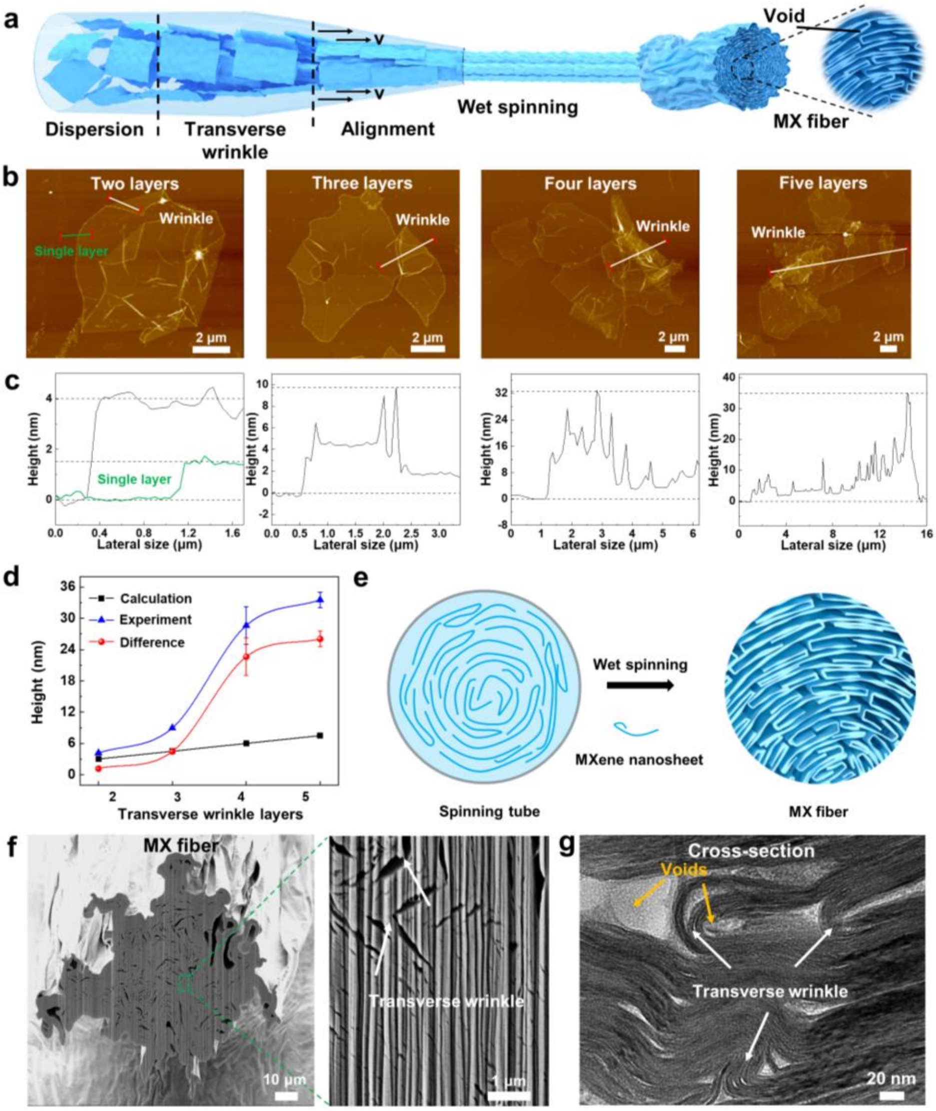

Supplementary Figure 2 | AFM images for the transverse wrinklers of MXene nanosheets.

a, The fabrication process of MX fiber with lots of voids generated caused by transverse wrinkles during the wet spinning. **b**, AFM images of the MXene nanosheets with different layers of transverse wrinkles, while the samples were obtained by spin coating at the speed of 500 rpm min^{-1}. **c**, The corresponding height of the MXene nanosheets with different layers of transverse wrinkles, while the single MXene nanosheet has a thickness of ~1.5 nm (green curve). **d**, The height of the calculation based on the single nanosheets of ~1.5 nm, experiment test obtained from the AFM, and the difference between the experiment and calculation with different layers of transverse wrinkles. All error bars show mean ± standard deviation (SD). **e**, MXene nanosheets with transverse wrinkles were assembled into MX fiber with more voids through wet spinning. **f**, SEM image of the cross-section for the MX fiber with the enlarged image. **g**, HR-TEM image of the cross-section for the MX fiber.

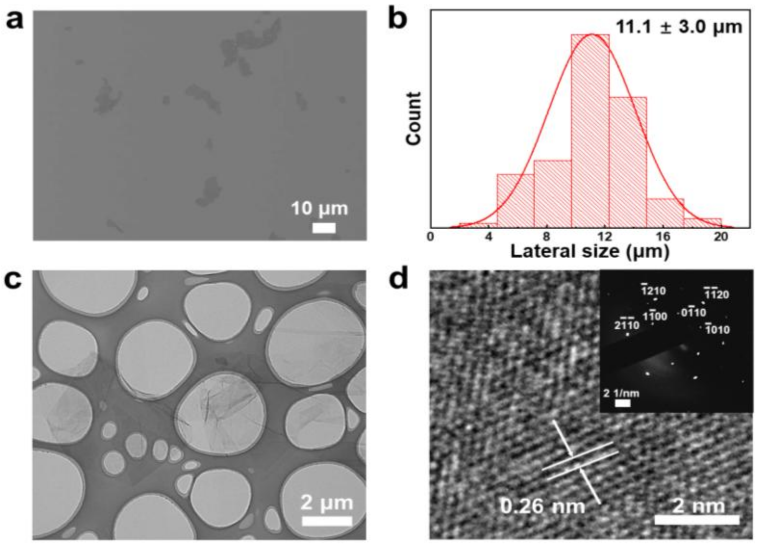

Supplementary Figure 3 | a, and **b,** SEM image and the size distribution for exfoliated MXene nanosheets with a lateral size of ~11.1 µm. **c,** TEM image and **d,** Corresponding HR-TEM image of exfoliated MXene nanosheets. The selected area electron diffraction pattern (inset in d) confirms the hexagonal single crystal structure without obvious defects. The MXene monolayer was further characterized by TEM, revealing well-defined lattice fringes with a spacing of ~0.26 nm, corresponding to the (100) plane of $Ti_3C_2T_x$, as shown in the HR-TEM images.

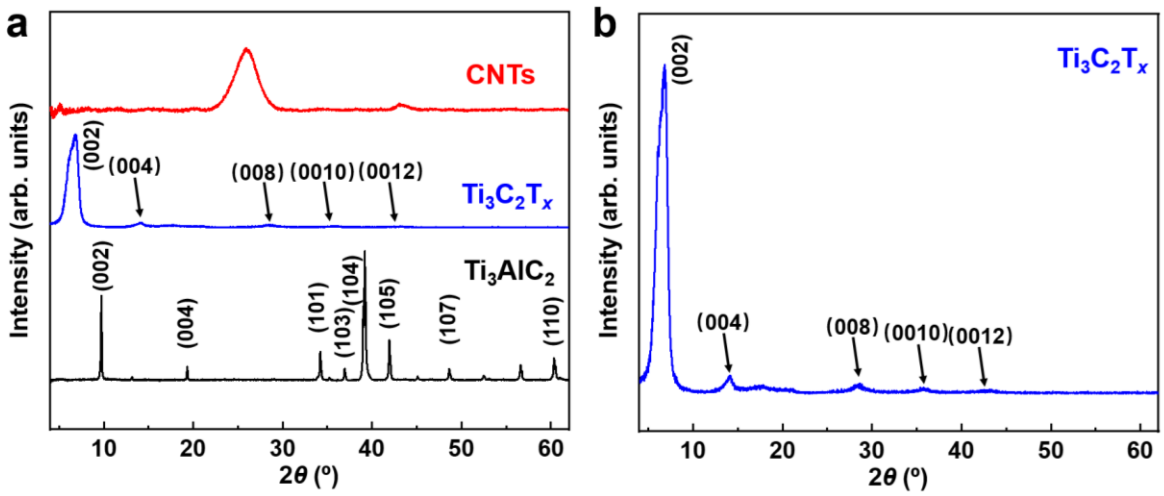

Supplementary Figure 4 | a, XRD patterns of Ti_3AlC_2, $Ti_3C_2T_x$ (MXene), and CNTs. According to the XRD patterns, the absence of (104) and (105) peaks demonstrates a complete removal of the Al layer from the Ti_3AlC_2, indicating the successful preparation of MXene nanosheets. **b**, XRD patterns of MXene show the (00l) diffractions.

Supplementary Note 3. Characterization of CNTs

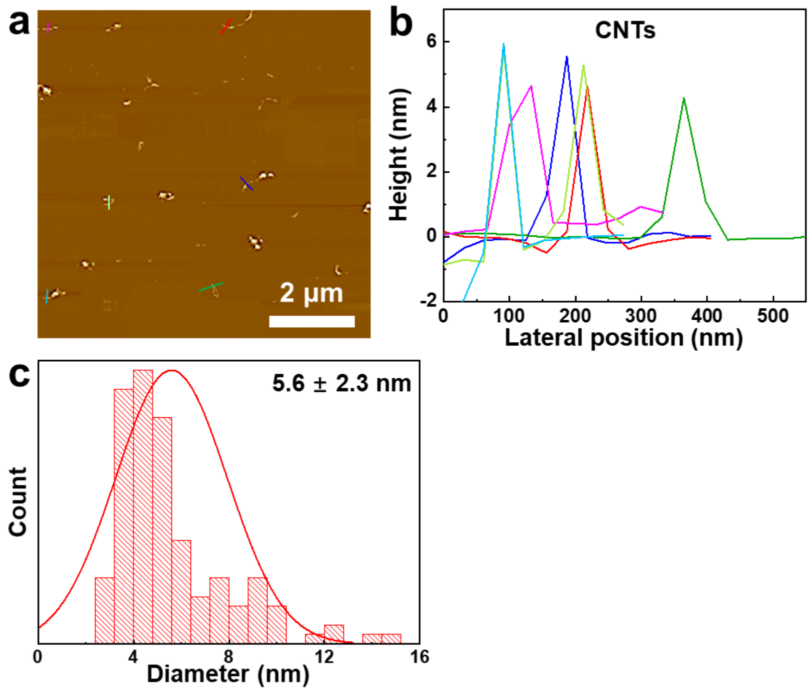

Supplementary Figure 5 | a, AFM images of CNTs with different lengths of ~0.46 μm. **b,** Height vs. lateral position of CNTs. **c,** The diameter distribution of CNTs. The results showed that the CNTs have a diameter range of 5.6 ± 2.3 nm.

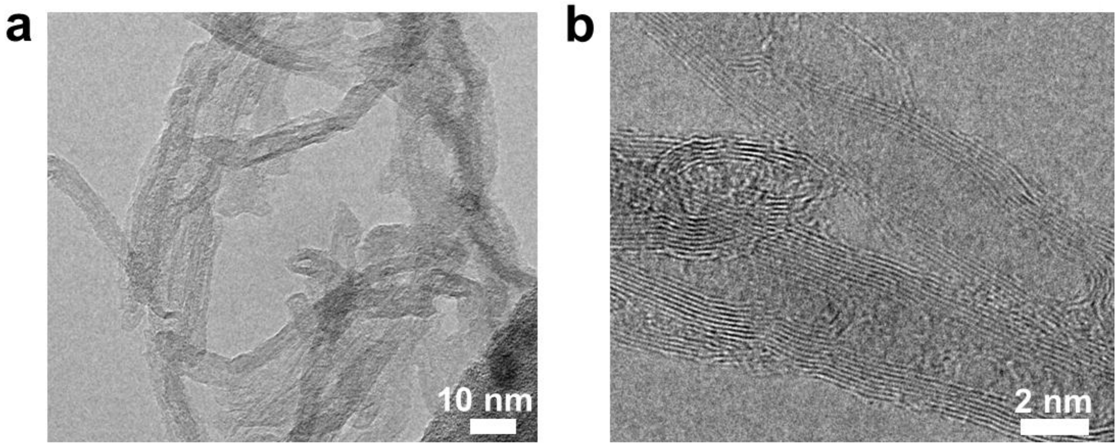

Supplementary Figure 6 | a, TEM image and **b,** Corresponding HR-TEM image of CNTs. The CNTs have a diameter of 5.6 ± 2.3 nm.

Supplementary Note 4. Liquid crystal properties and rheological properties of spinning dispersion

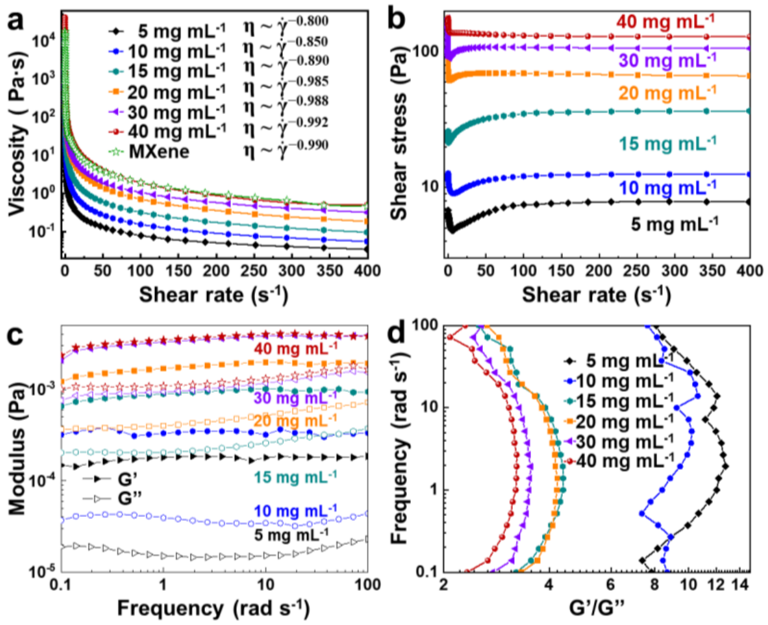

Supplementary Figure 7 | Viscosity (**a**) and Shear stress (**b**) as a function of shear rate (s^{-1}) with different concentrations of MXene-CNTs spinning dispersion from 5 mg mL^{-1} to 40 mg mL^{-1}, and pure MXene spinning dispersion with the concentration of 40 mg mL^{-1}. **c,** Storage modulus (G') and loss modulus (G'') as a function of frequency (rad s^{-1}). **d,** G'/G'' as a function of frequency (rad s^{-1}).

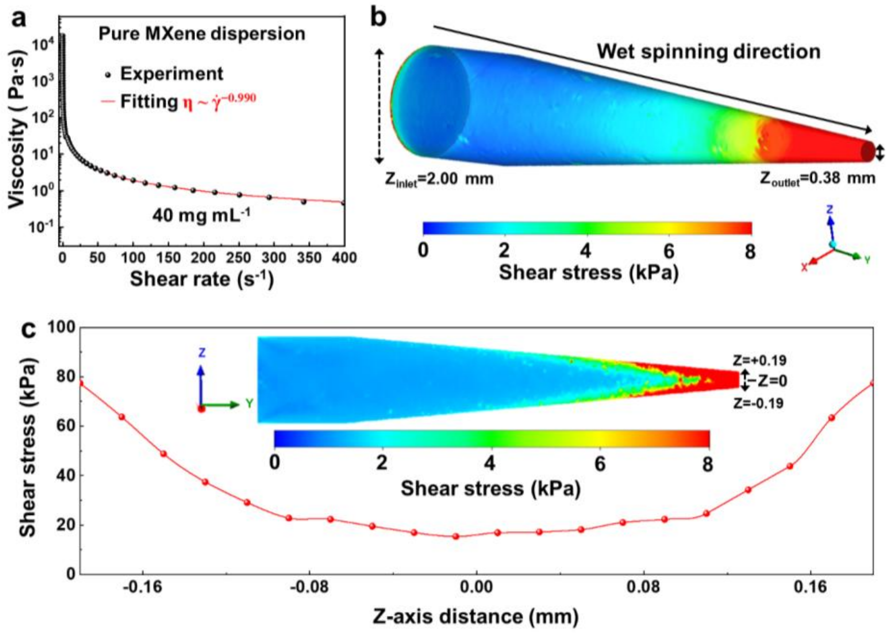

Supplementary Figure 8 | Finite element analysis (FEA) of the wet spinning process. a, Viscosity as a function of shear rate of pure MXene nanosheets spinning dispersion at a concentration of 40 mg mL^{-1}. According to the fitting curve of power law, the flow power-law index of n is ~0.01 less than 1, exhibiting shear-thinning for spinning dispersion. **b**, FEA model of shear stress distribution for pure MXene spinning dispersion during the wet spinning process. **c**, The shear stress along the Z-axis distance according to the FEA simulation. The origin of the Z-axis (Z=-0.19) corresponds to the lower part of the spinning tube outlet, and the endpoint corresponds to the upper part (Z=+0.19) of the outlet.

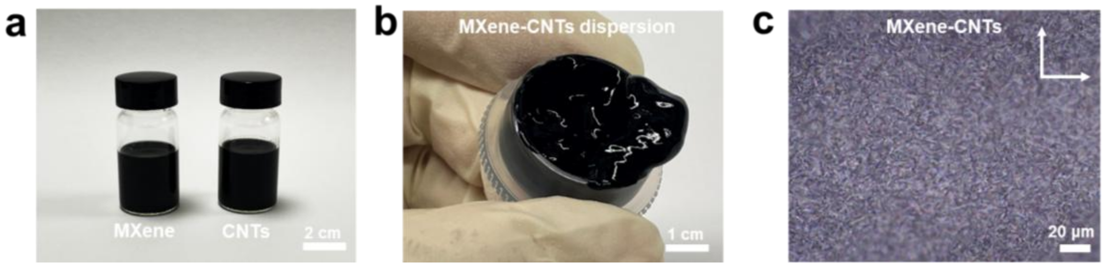

Supplementary Figure 9 | a, Photo of MXene and CNTs dispersion. **b,** Photo of MXene-CNTs dispersion with the concentration of 40 mg mL^{-1} for wet spinning. **c,** POM image of MXene-CNTs spinning dispersion with the concentrations of 40 mg mL^{-1}, exhibiting optical birefringence.

Supplementary Note 5. Interpretation of FTIR and XPS spectra of MXene composite fibers

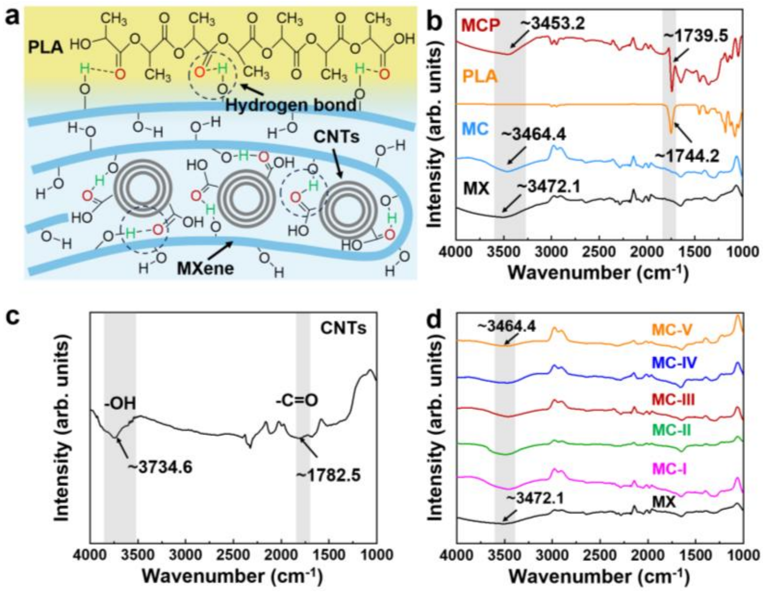

Supplementary Figure 10 | FTIR spectra of MC fibers. **a**, Cartoon illustration of interfacial interactions in MCP fiber. **b**, FTIR spectra of the obtained fibers including MX, MC, PLA, and MCP fibers. **c,** FTIR spectrum of the CNTs. The spectrum revealed a peak at a wavenumber of ~3734.6 cm^{-1} for the -OH group and another at ~1782.5 cm^{-1} for the -C=O group, indicating the presence of -COOH groups on the surface of the CNTs. **d,** FTIR spectra show that the wavenumber of ~3472.1 cm^{-1} for hydroxyl functional group (-OH) of MXene nanosheets for MX is shifted to that of ~3464.4 cm^{-1} for MC fibers with different weight percentages of CNTs, indicating the formation of hydrogen bonds between MXene nanosheets and CNTs.

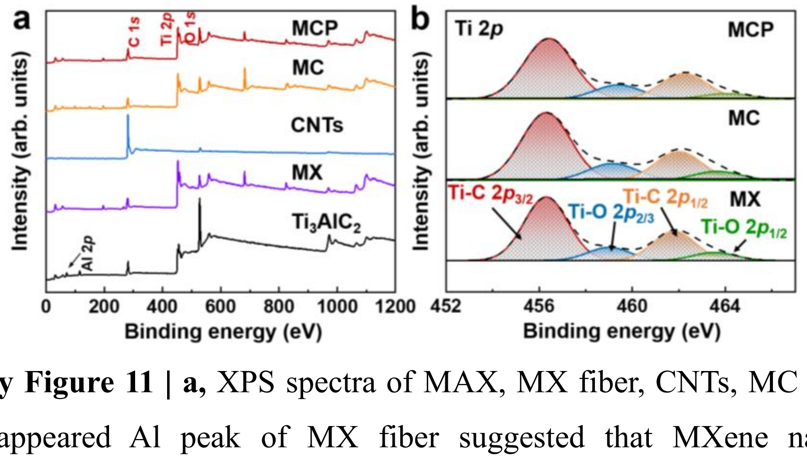

Supplementary Figure 11 | a, XPS spectra of MAX, MX fiber, CNTs, MC fiber, and MCP fiber. The disappeared Al peak of MX fiber suggested that MXene nanosheets have successfully etched from the primitive Ti_3AlC_2. Meanwhile, the increased percentage of O in MC and MCP fibers indicated the formation of hydrogen bonds between CNTs and MXene nanosheets, as well as PLA and MXene nanosheets. **b**, Ti 2*p* spectra of MX, MC, and MCP fibers.

Supplementary Note 6. Photo of MXene composites fibers

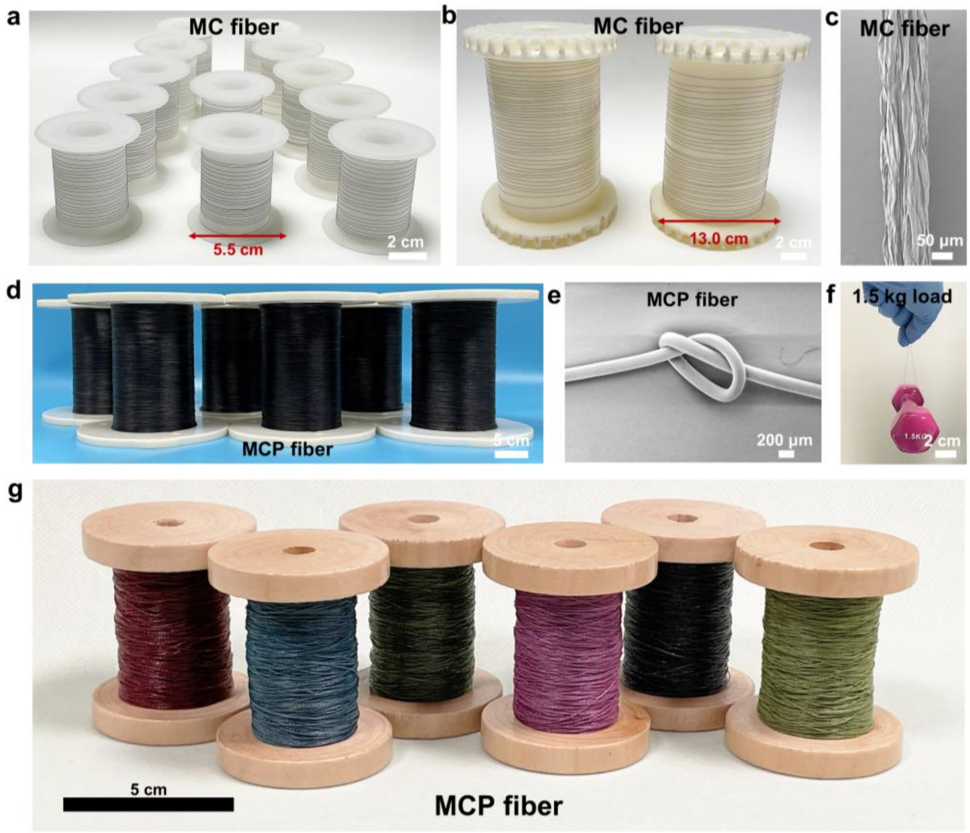

Supplementary Figure 12 | a and **b,** Photographs of MC fiber with the same diameter wound around reels with two different diameters: 5.5 cm and 13.0 cm. **c**, SEM image of the axis of the MC fiber. **d**, Photographs of MCP fiber with thousands of meters long. **e**, SEM image of a knotted MCP fiber. **f**, Photograph of the MCP fiber lifting a 1.5 kg load. **g**, MCP fibers of various colors were prepared with a dyeing efficiency of 10 meters per minute. The obtained MCP fibers were dyed in color baths of different hues at 70 °C, with a dyeing speed of 10 meters per minute. Afterward, the fibers were dried at room temperature for 24 hours to obtain MCP fibers in various colors.

Supplementary Note 7. nano-CT of MXene composites fibers

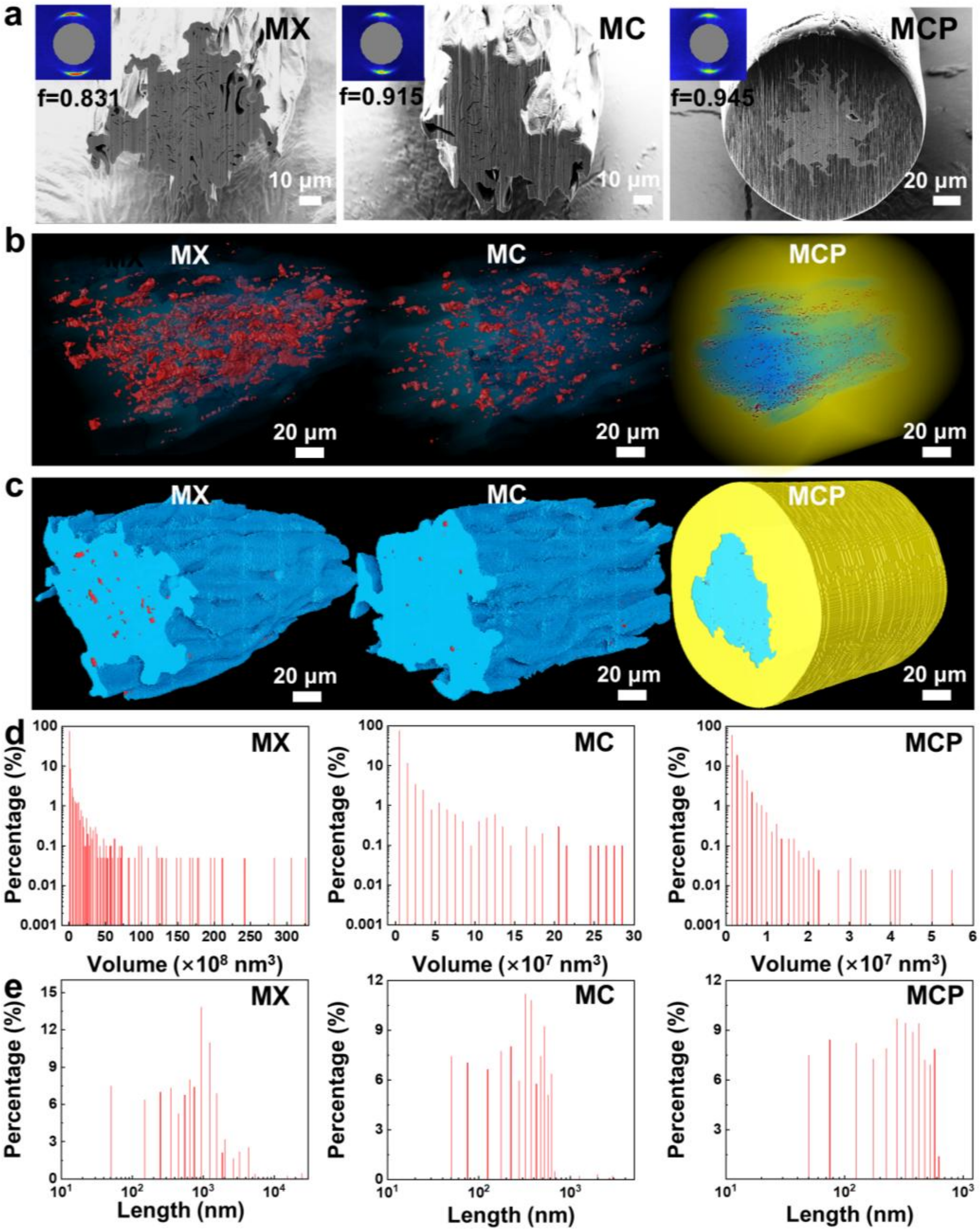

Supplementary Figure 13 | SEM images and 3D reconstruction of the MXene composite fibers through nano-CT. a, SEM images of the cross-section for MX, MC, and MCP fibers (the insets for WAXS patterns). **b**, Three-dimensional (3D) reconstruction of the axial-section nano-CT and fibers of MX, MC, and MCP fibers. **c**, 3D reconstruction of the whole fibers with voids within it, while the blue for MXene in MX fiber, and that for MXene and CNTs in MC fiber, including the red for voids, and the yellow for the PLA. **d**, The volume distributions of the voids in the MXene composite fibers of MX, MC, and MCP fibers. **e**, The length distributions of the voids in the MXene composite fibers of MX, MC, and MCP fibers along the axial of the fibers.

Supplementary Note 8. Porosity and orientation factor of MC fibers fabricated with different lengths of CNTs

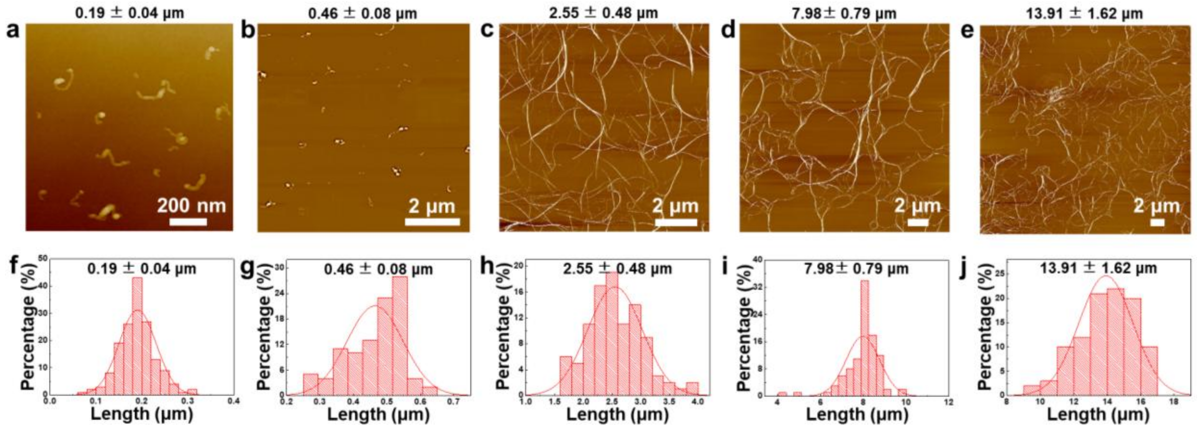

Supplementary Figure 14 | Distribution of length for CNTs with different lengths according to the AFM images. **a**, The length of 0.19 ± 0.04 μm. **b**, The length of 0.46 ± 0.08 μm. **c**, The length of 2.55 ± 0.48 μm. **d**, The length of 7.98 ± 0.79 μm. **e**, The length of 13.91 ± 1.62 μm. **f-j**, Length distribution curve of CNTs ranging from ~0.19 μm to ~13.91 μm. All error bars show mean ± SD.

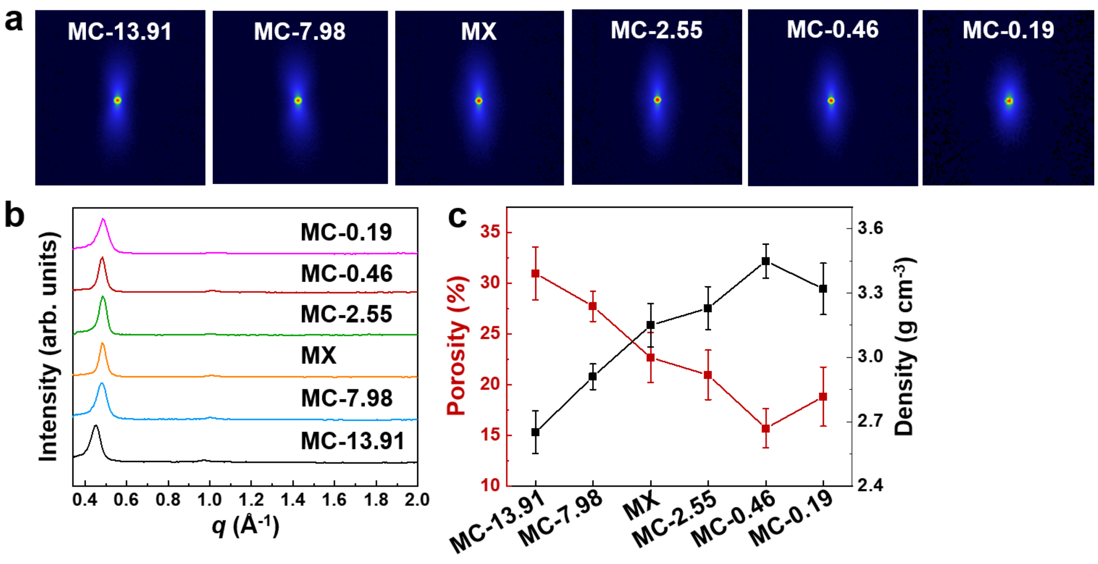

Supplementary Figure 15 | a, SAXS patterns of MC fibers fabricated with different lengths of CNTs from ~0.19 μm to ~13.91 μm at the weight percentage of 1%. MC-13.91 fiber was fabricated from a length of ~13.91 μm. MC-7.98 fiber was fabricated from a length of ~7.98 μm. MC-2.55 fiber was fabricated from a length of ~2.55 μm. MC-0.46 fiber was fabricated from a length of ~0.46 μm. MC-0.19 fiber was fabricated from a length of ~0.19 μm. **b,** XRD patterns of the fabricated fibers. **c,** The porosity and density of the fabricated MC fibers. All error bars show mean ± SD.

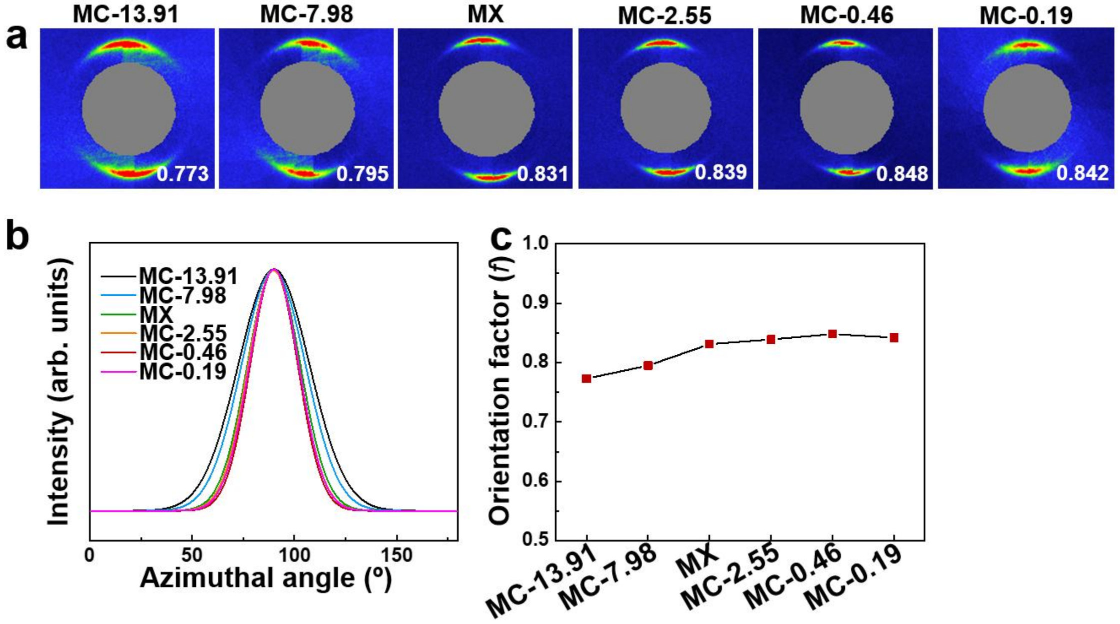

Supplementary Figure 16 | a, WAXS patterns of MC fibers fabricated with different lengths of CNTs from ~0.46 μm to ~13.91 μm at the weight percentage of 1%. MC-13.91 fiber was fabricated from a length of ~13.91 μm. MC-7.98 fiber was fabricated from a length of ~7.98 μm. MC-2.55 fiber was fabricated from a length of ~2.55 μm. MC-0.46 fiber was fabricated from a length of ~0.46 μm. MC-0.19 fiber was fabricated from a length of ~0.19 μm. **b,** Plots of the azimuthal angle of the fabricated fibers. **c,** The *f* of the fabricated MC fibers.

Supplementary Note 9. SEM images and EDS mapping of MC fibers fabricated with different lengths of CNTs

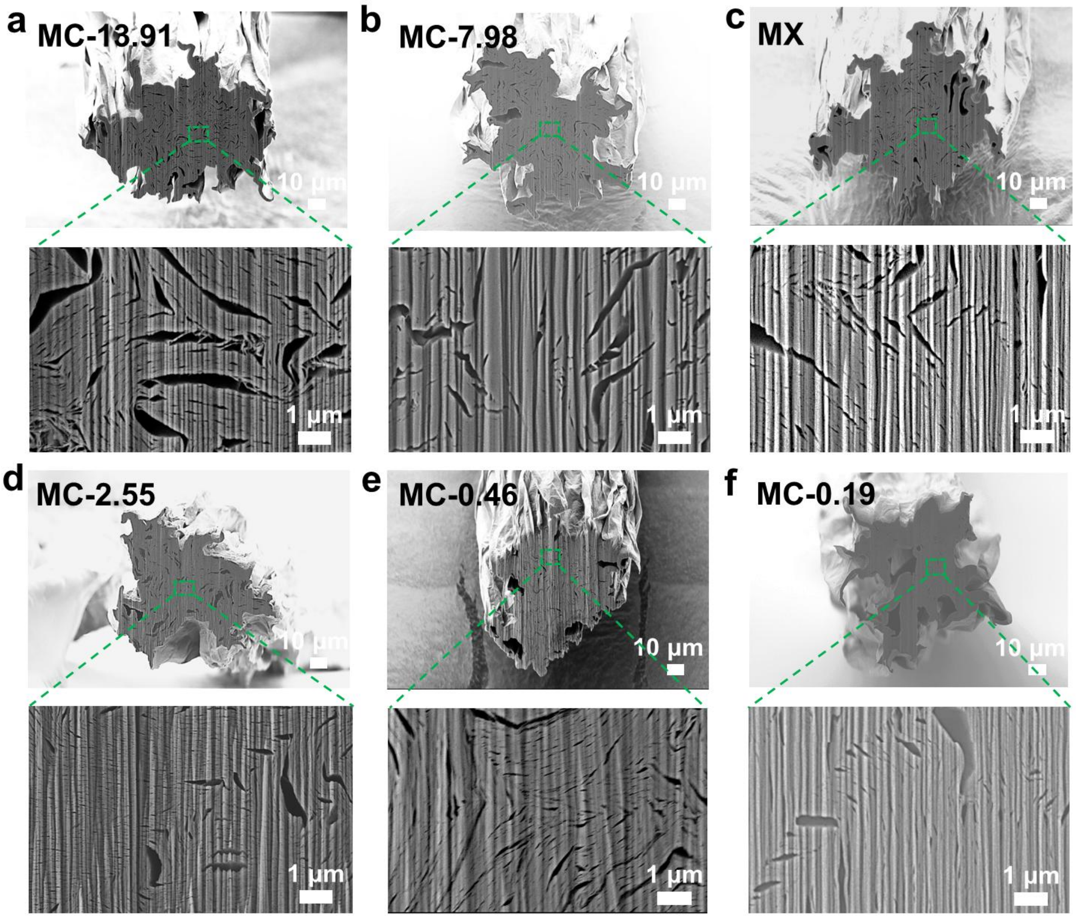

Supplementary Figure 17 | SEM images of the cross-sections of MC fibers fabricated with different lengths of CNTs from ~0.19 μm to ~13.91 μm at a weight percentage of 1%. **a,** MC-13.91 fiber fabricated from the length of ~13.91 μm. **b,** MC-7.98 fiber fabricated from the length of ~7.98 μm. **c,** MX fiber. **d,** MC-2.55 fiber fabricated from the length of ~2.55 μm. **e,** MC-0.46 fiber fabricated from the length of ~0.46 μm. **f,** MC-0.19 fiber fabricated from the length of ~0.19 μm.

Supplementary Note 10. Mechanical and electrical properties of MC fibers fabricated with different lengths of CNTs

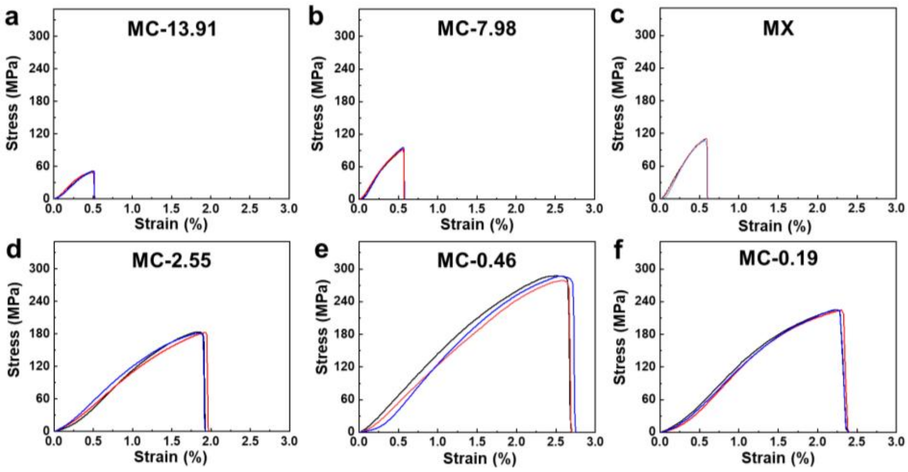

Supplementary Figure 18 | Stress-strain curves for MC fibers fabricated with different lengths of CNTs from ~0.19 μm to ~13.91 μm at the weight percentage of 1%. **a,** MC-13.91 fiber fabricated from the length of ~13.91 μm. **b,** MC-7.98 fiber fabricated from the length of ~7.98 μm. **c,** MX fiber. **d,** MC-2.55 fiber fabricated from the length of ~2.55 μm. **e,** MC-0.46 fiber fabricated from the length of ~0.46 μm. **f,** MC-0.19 fiber fabricated from the length of ~0.19 μm.

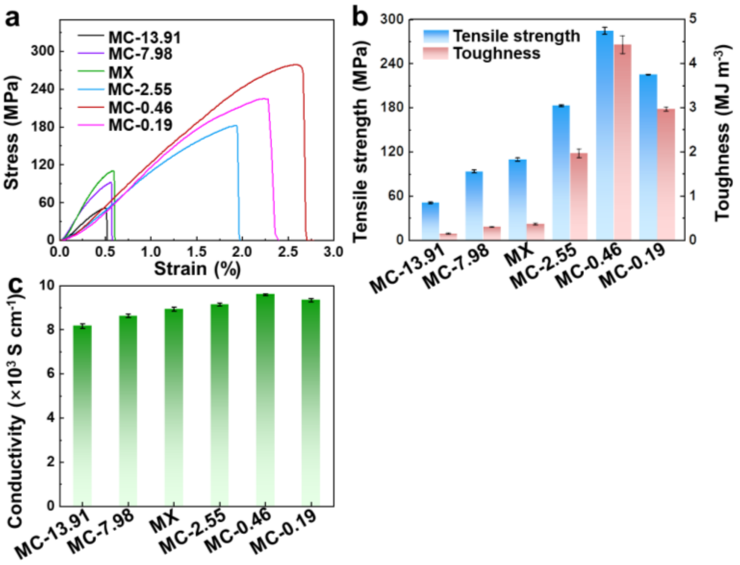

Supplementary Figure 19 | a, Stress-strain curves of MC fibers fabricated with different lengths of CNTs from ~0.16 μm to ~13.91 μm at the weight percentage of 1%, in which MC-13.91 fiber was fabricated from CNTs with the length of ~13.91 μm, MC-7.98 fiber was fabricated from CNTs with the length of ~7.98 μm, MC-2.55 fiber was fabricated from CNTs with the length of ~2.55 μm, MC-0.46 fiber was fabricated from CNTs with the length of ~0.46 μm, and MC-0.19 fiber was fabricated from CNTs with the length of ~0.19 μm. **b**, Tensile strength and toughness of the fabricated MC fibers. **c**, Conductivities of the fabricated MC fibers. All error bars show mean ± SD.

Supplementary Note 11. Porosity and orientation factor of MC fibers fabricated with different weight percentages of CNTs

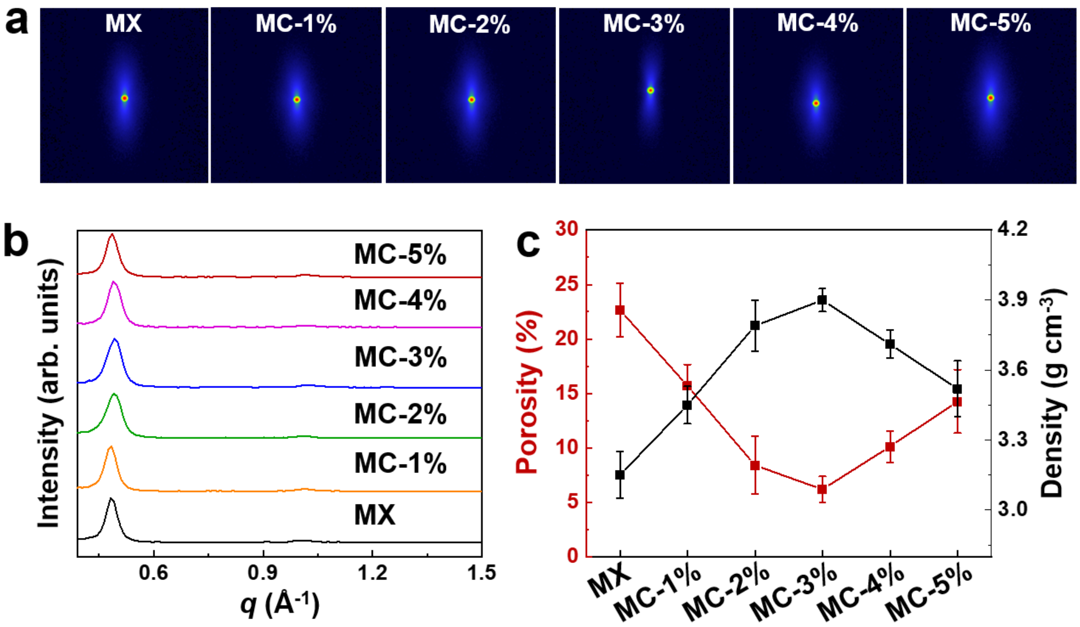

Supplementary Figure 20 | a, SAXS patterns of MC fibers fabricated with different weight percentages of CNTs from 1% to 5% with a length of ~0.46 μm. **b,** XRD patterns of the fabricated fibers with different weight percentages of CNTs. **c,** The porosity and density of the fabricated MC fibers with different weight percentages of CNTs. All error bars show mean ± SD.

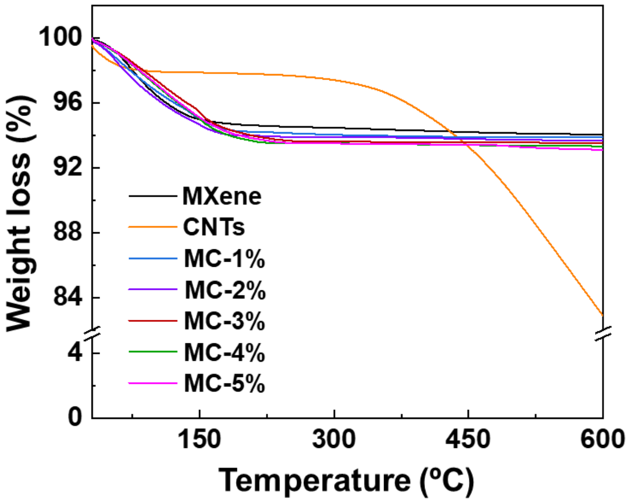

Supplementary Figure 21 | TGA curves of pure MXene, CNTs, MC-1%, MC-2%, MC-3%, MC-4%, and MC-5%. These results were obtained in a nitrogen atmosphere using a heating rate of 10 K min^{-1}. The component content of MXene and CNTs in MC fibers were calculated

according to the TGA curves. M_{MXene}, M_{CNTs}, and M_{MC} are the fractions of weight loss for MXene, CNTs, and MC fibers fabricated with different weight percentage of CNTs. The weight content (W_{CNTs}) of CNTs in the MC fiber was calculated using equation (1). The results are shown in Supplementary Table 4, indicating that MC-3% contains an actual 3.2 wt% of CNTs. We have added the figure and table in in the revised Supplementary Information.

$$W_{CNTs}=\frac{M_{MC}\text{-}M_{MXene}}{M_{CNTs}\text{-}M_{MXene}} \quad (13)$$

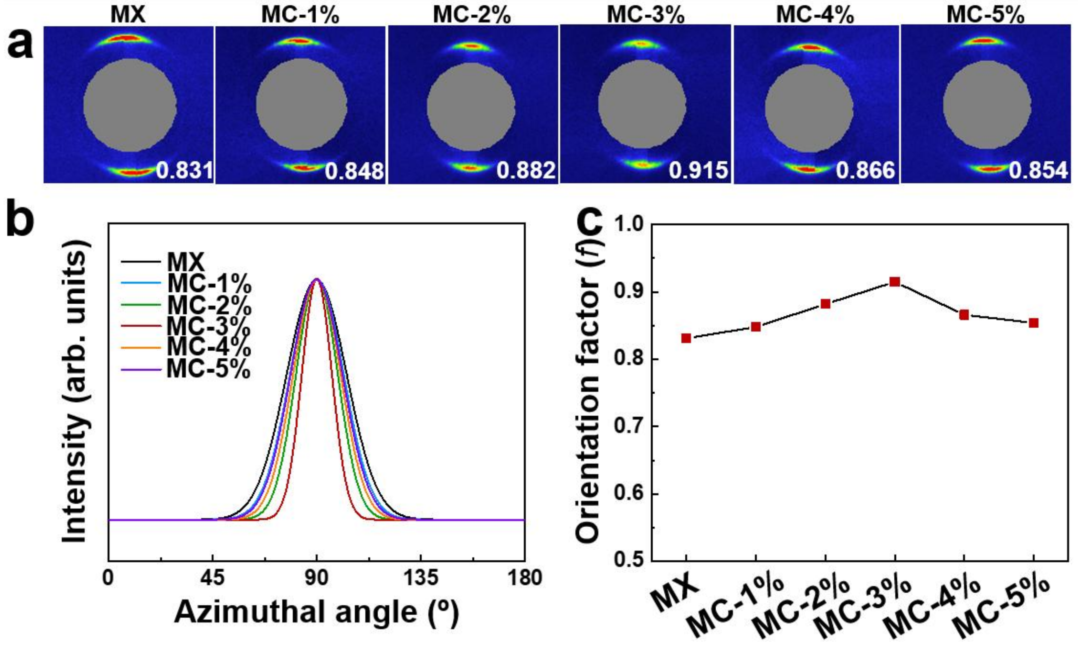

Supplementary Figure 22 | a, WAXS patterns of MC fibers fabricated with different weight percentages of CNTs from 1% to 5% with a length of ~0.46 μm, which MC-1% to MC-5% fiber were fabricated with 1 wt%, 2 wt%, 3 wt%, 4 wt%, and 5 wt% CNTs. **b,** Plots of azimuthal angle of the fabricated fibers with different weight percentages of CNTs. **c,** The *f* of the fabricated MC fibers with different weight percentages of CNTs.

Supplementary Note 12. SEM images and EDS mapping of MC fibers fabricated with different weight percentages of CNTs

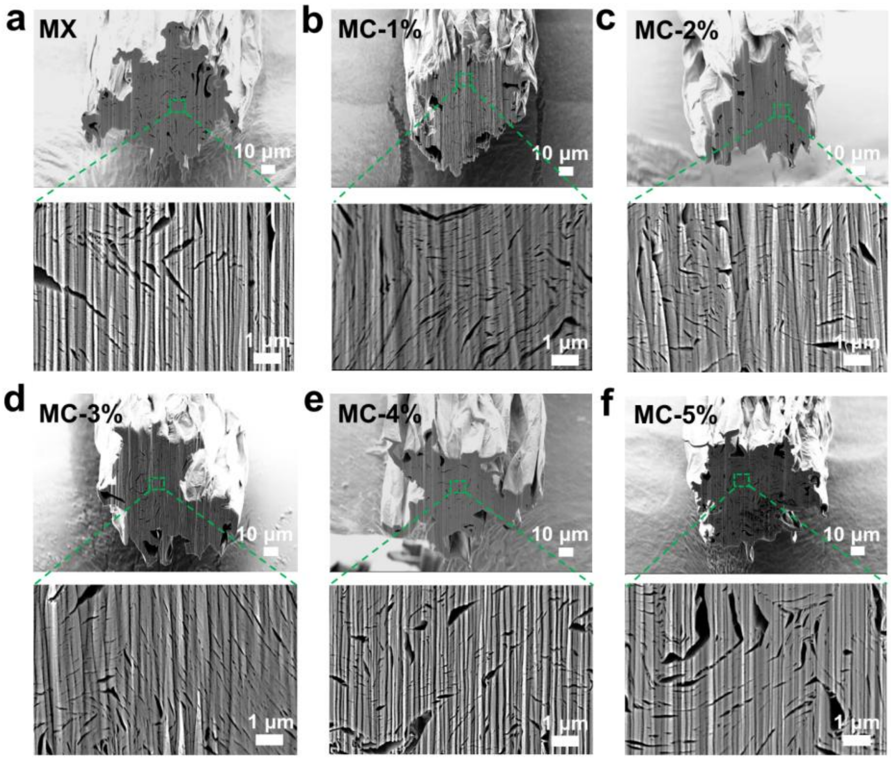

Supplementary Figure 23 | SEM images of the cross-section for MC fibers. SEM images of the cross-sections of MC fibers fabricated with different weight percentages of short CNTs with a length of ~0.46 µm. **a**, MX fiber. **b**, MC-1% fiber for the weight percentage of 1% of short CNTs. **c**, MC-2% fiber for the weight percentage of 2% of short CNTs. **d**, MC-3% fiber for the weight percentage of 3% of short CNTs. **e**, MC-4% fiber for the weight percentage of 4% of short CNTs. **f**, MC-5% fiber for the weight percentage of 5% of short CNTs.

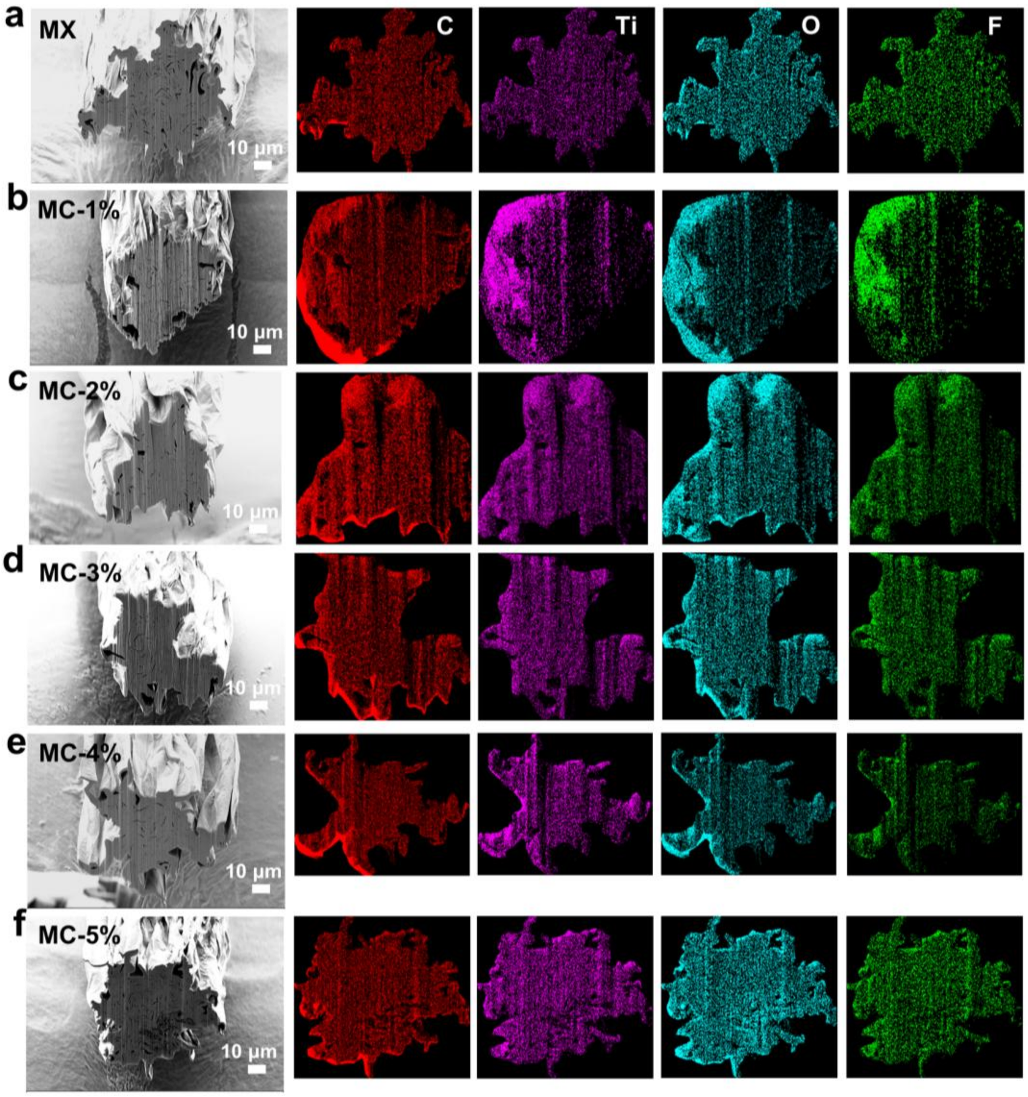

Supplementary Figure 24 | SEM images and the corresponding EDS mappings of the cross-sections of MC fibers fabricated with different weight percentages of short CNTs with the length of ~0.46 μm. **a,** MX fiber. **b,** MC-1% fiber for the weight percentage of 1% of short CNTs. **c,** MC-2% fiber for the weight percentage of 2% of short CNTs. **d,** MC-3% fiber for the weight percentage of 3% of short CNTs. **e,** MC-4% fiber for the weight percentage of 4% of short CNTs. **f,** MC-5% fiber for the weight percentage of 5% of short CNTs.

Supplementary Note 13. Characterization of MC fibers fabricated through different diameters of spinning nozzle

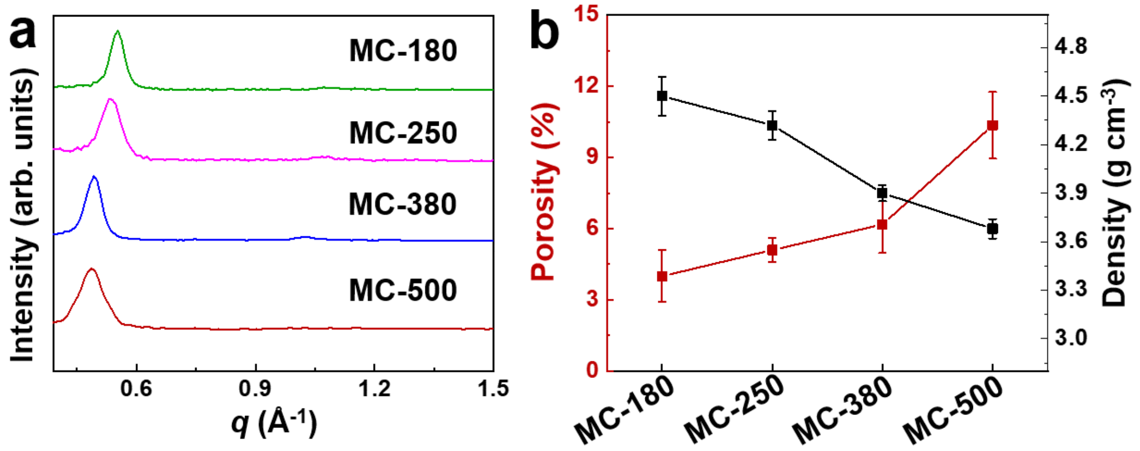

Supplementary Figure 25 | a, XRD patterns of the MC fibers through different spinning nozzle with diameters from 180 μm to 500 μm with ~3 wt% CNTs in a spinning solution concentration of ~40 mg mL^{-1}. **b**, The porosity and density of the MC fibers fabricated through different spinning nozzle diameters. All error bars show mean ± SD.

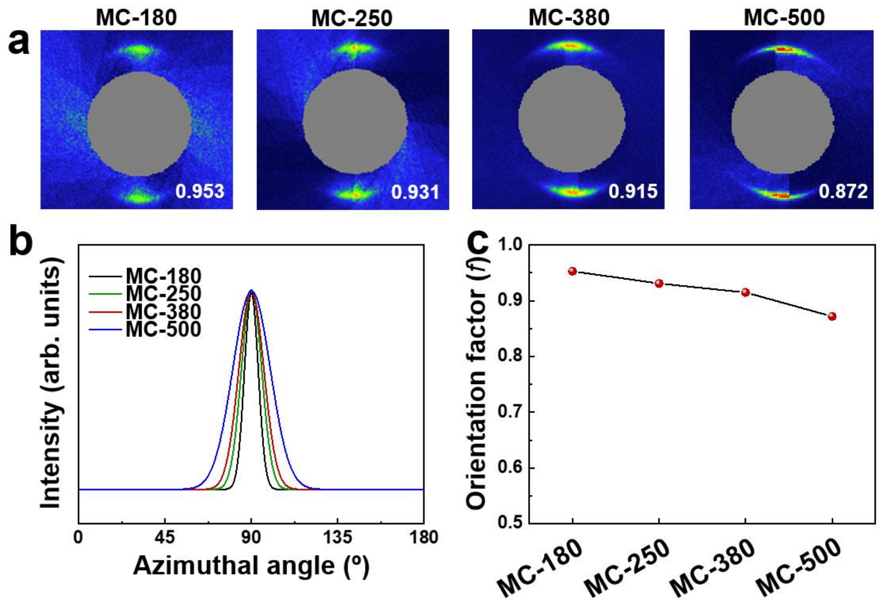

Supplementary Figure 26 | Orientation factor of MC fibers fabricated through different spinning nozzle with diameters from 180 μm to 500 μm according to the WAXS pattern. **a**, WAXS patterns of MC fibers fabricated through different spinning nozzle with diameters from 180 μm, 250 μm, 380 μm, and 500 μm. These fibers, labeled as MC-180, MC-250, MC-380, and MC-500, were fabricated with ~3 wt% CNTs in a spinning solution concentration of ~40 mg mL^{-1}. **b**, Plots of azimuthal angle of the fabricated fibers. **c**, The orientation factor (*f*) of the MC fibers fabricated through different spinning nozzle diameters.

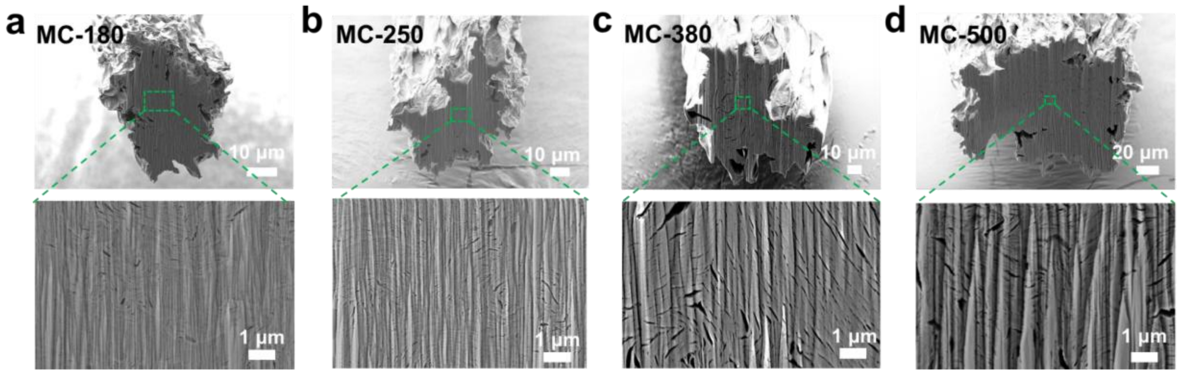

Supplementary Figure 27 | SEM images of the cross-section for MC fibers fabricated through different spinning nozzle with diameters from 180 μm to 500 μm. These fibers, labeled as MC-180 to MC-500, were fabricated with ~3 wt% CNTs in a spinning solution concentration of ~40 mg mL^{-1}. **a**, MC-180 fiber fabricated through the spinning nozzle diameter of 180 μm. **b**, MC-250 fiber fabricated through the spinning nozzle diameter of 250 μm. **c**, MC-380 fiber fabricated through the spinning nozzle diameter of 250 μm. **d**, MC-500 fiber fabricated through the spinning nozzle diameter of 500 μm.

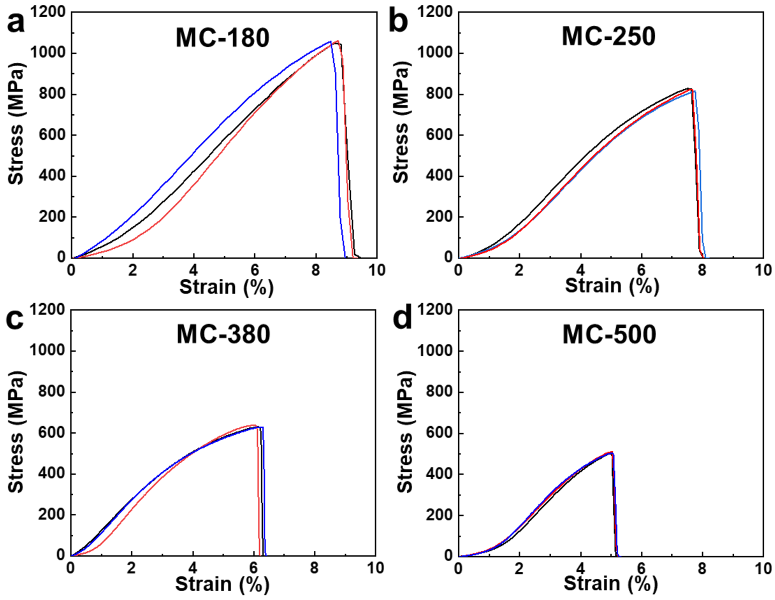

Supplementary Figure 28 | Stress-strain curves of MC fiber fabricated through different spinning nozzle with diameters from 180 μm to 500 μm. a, MC-180 fiber fabricated through the spinning nozzle diameter of 180 μm. **b**, MC-250 fiber fabricated through the spinning nozzle diameter of 250 μm. **c**, MC-380 fiber fabricated through the spinning nozzle diameter of 250 μm. **d**, MC-500 fiber fabricated through the spinning nozzle diameter of 500 μm.

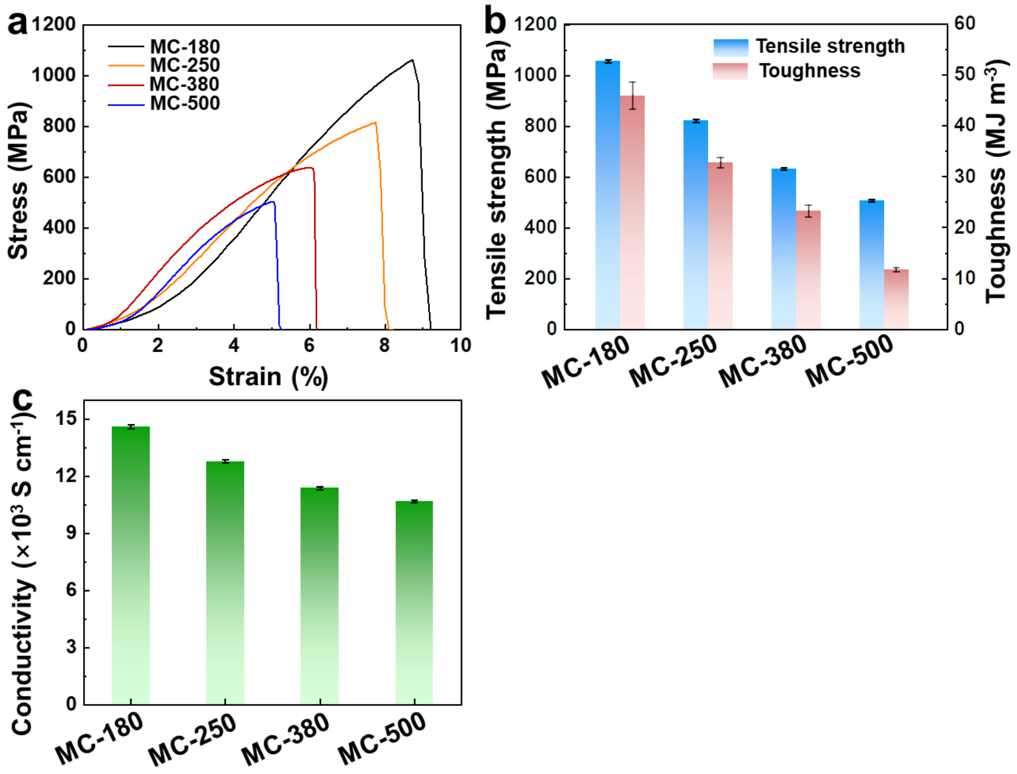

Supplementary Figure 29 | a, The stress-strain curves of the obtained MC fibers fabricated through different spinning nozzle with diameters from 180 μm to 500 μm with ~3 wt% CNTs in a spinning solution concentration of ~40 mg mL^{-1}. **b**, The tensile strengths and toughness of MC fibers fabricated through different spinning nozzle with diameters from 180 μm to 500 μm. **c**, The conductivities of MC fibers fabricated through different spinning nozzle with diameters from 180 μm to 500 μm. All error bars show mean ± SD.

Supplementary Note 14. FEA simulation and in-situ XRD characterization of MC-3% fiber being heated

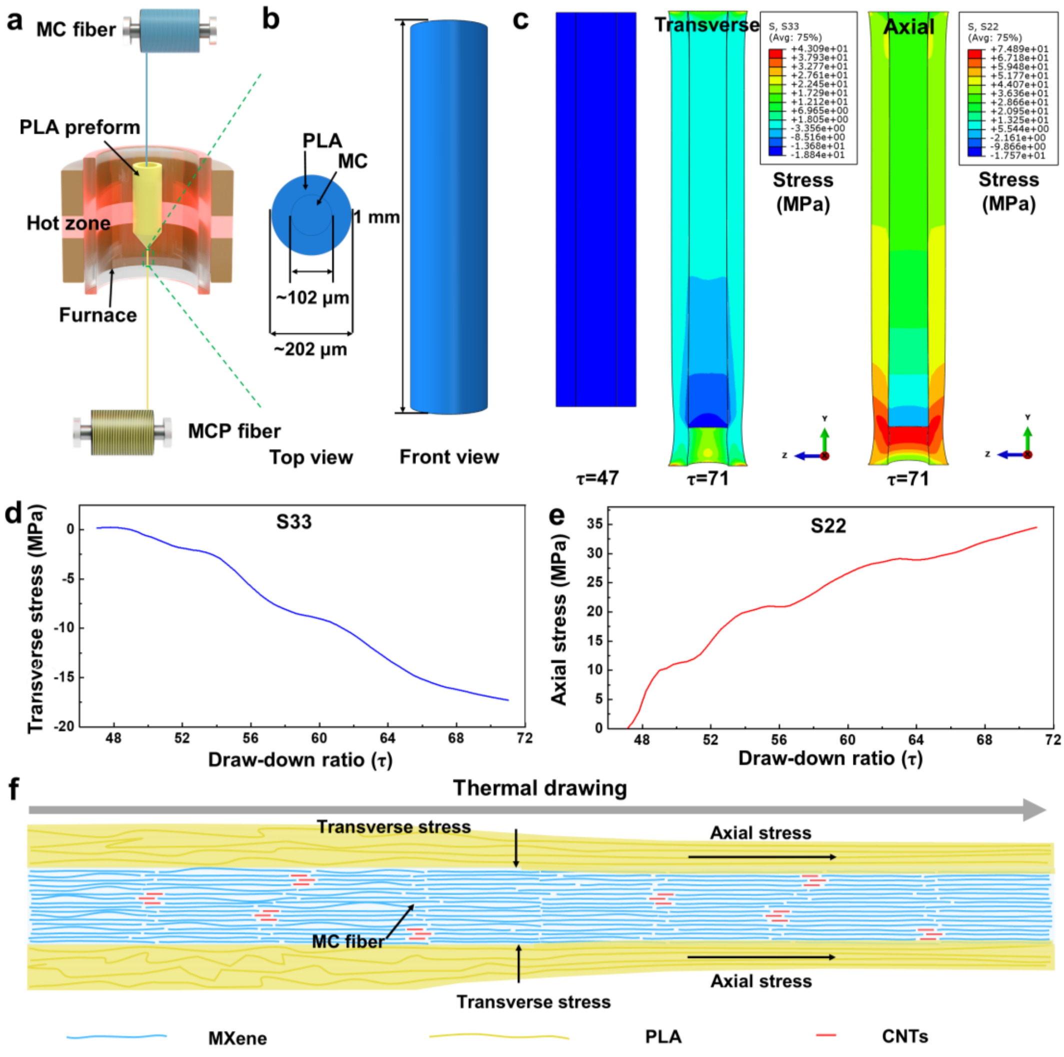

Supplementary Figure 30 | The FEA of mechanical behavior of inner MC fibers in the process of thermal drawing. a, The dynamic thermal drawing process for fabricating MCP fiber. **b**, A FEA model of MC fiber in the PLA preforms during the dynamic thermal drawing, while the inner MC fiber with a diameter of ~102.0 μm and PLA preforms with a diameter of ~202.0 μm. The height of the model was constructed as 1.0 mm. **c**, Cloud images of transverse and axial stresses distribution of the FEA simulation at the increased draw-down ratio up to 71. The transverse (**d**) and axial (**e**) stresses at the increment of draw-down ratio according to FEA. **f**, Illustration of the mechanical behavior of inner MC fibers in the dynamic process of thermal drawing.

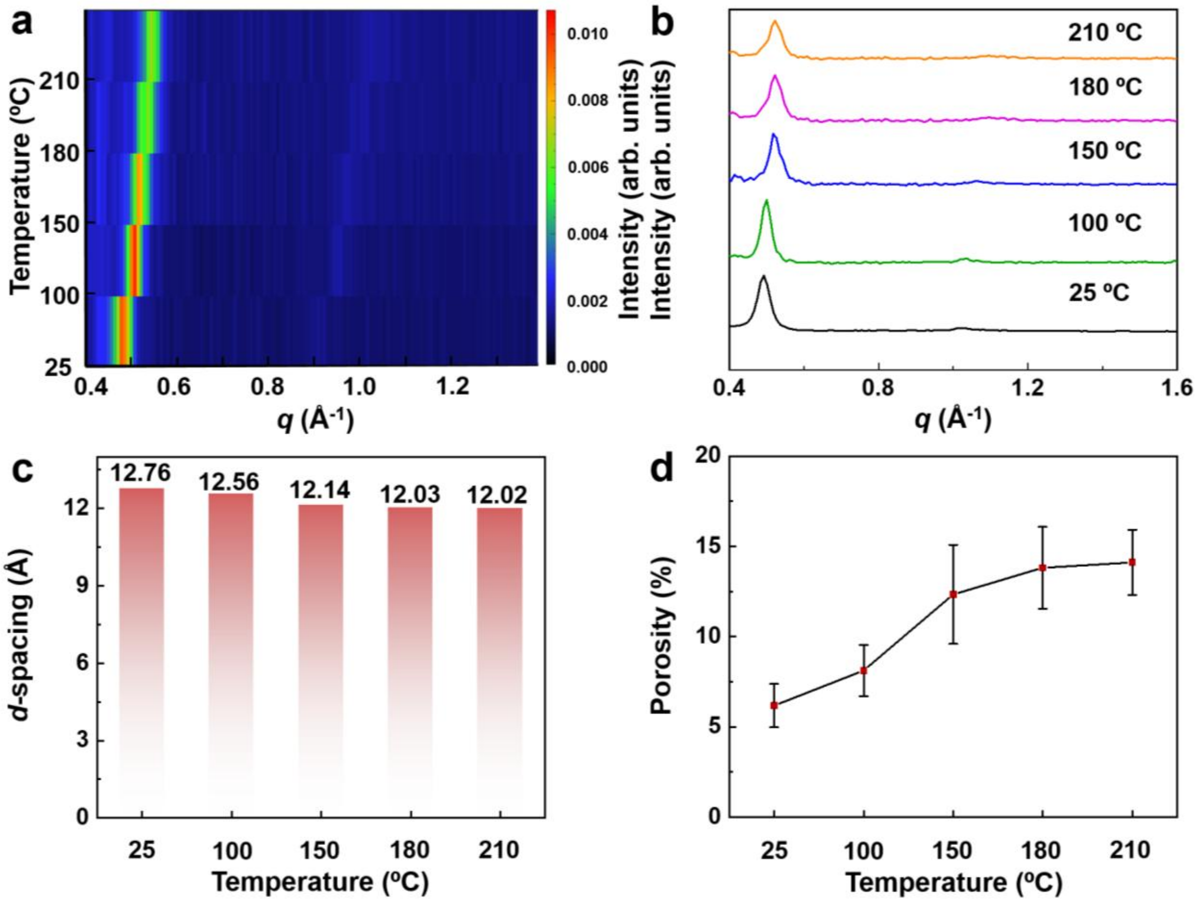

Supplementary Figure 31 | In-situ XRD patterns of fabricated MC fibers being heated under variable temperatures from 25 °C to 210 °C. **a,** A schematic diagram of MC-3% fibers being heated. **b,** In-situ XRD patterns of fabricated MC-3% fibers being heated under variable temperatures from 25 °C to 210 °C. **c,** The *d*-spacing between MXene nanosheets was decreased with the increment of heating temperature. **d,** The porosity of MC-3% fibers under variable temperatures from 25 °C to 210 °C. All error bars show mean ± SD.

Supplementary Note 15. Porosity and orientation factor of MCP fibers

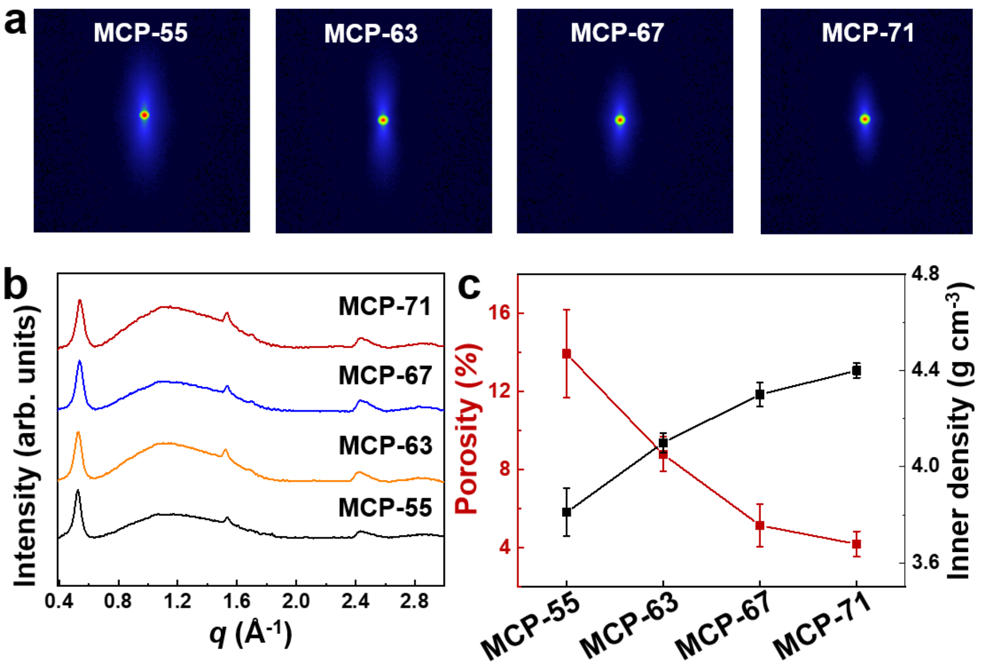

Supplementary Figure 32 | a, SAXS patterns of MCP fibers fabricated by thermal drawing at the increasing draw-down ratios. The MCP fiber were fabricated through dynamic thermal drawing using various τ of 55, 63, 67, and 71, and were labeled as MCP-55, MCP-63, MCP-67, and MCP-71, respectively. **b,** XRD patterns of the fabricated fibers fabricated by thermal drawing at the increasing draw-down ratios. **c,** The porosity and density of the MCP fibers fabricated by thermal drawing at the increasing draw-down ratios. All error bars show mean ± SD.

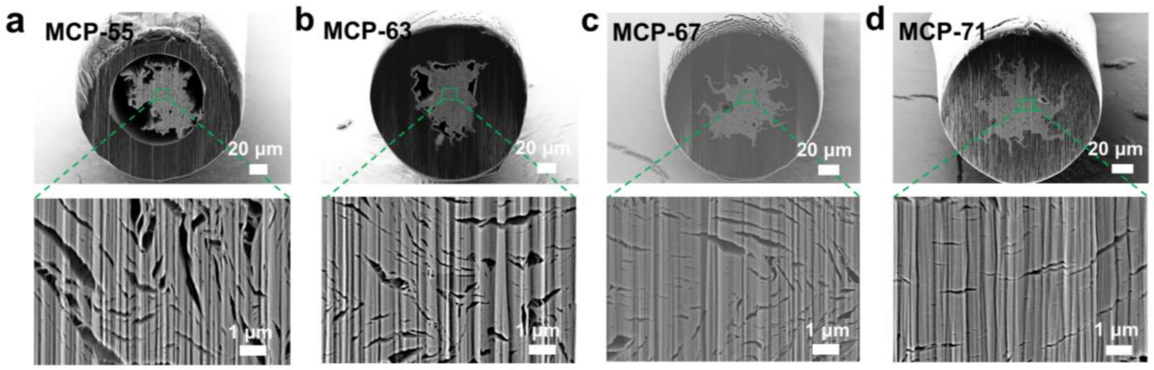

Supplementary Figure 33 | SEM cross-section of MCP fibers fabricated by different τ. **a**, MCP-55 fiber fabricated by the τ of 55. **b**, MCP-63 fiber fabricated by the τ of 63. **c**, MCP-67 fiber fabricated by the τ of 67. **d**, MCP-71 fiber fabricated by the τ of 71. The results showed that MCP fibers get more compact to significantly reduce the porosity and enhance the alignment of fibers with the increment of the τ.

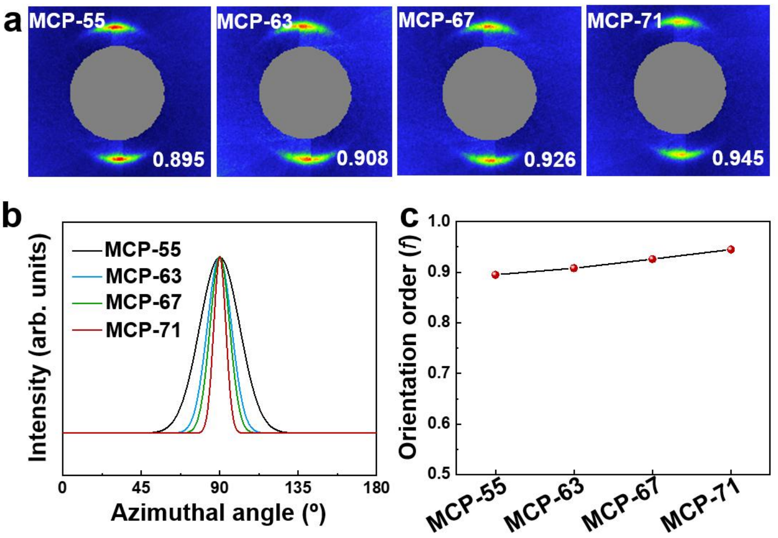

Supplementary Figure 34 | a, WAXS patterns of MCP fibers fabricated through thermal drawing at increasing draw-down ratios. **b,** Plots of azimuthal angle of the MCP fibers fabricated through thermal drawing at increasing draw-down ratios according to the WAXS pattern. **c,** The *f* of the MCP fibers fabricated through thermal drawing at increasing draw-down ratios according to the WAXS pattern.

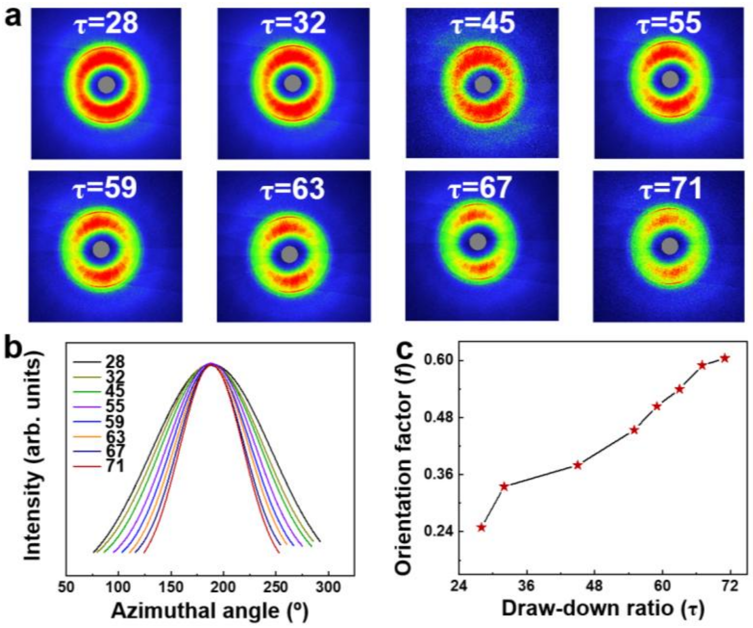

Supplementary Figure 35 | a, WAXS patterns of the PLA fibers fabricated by thermal drawing at various draw-down ratios. **b,** Plots of azimuthal angle of PLA fibers fabricated by thermal drawing at various draw-down ratios according to WAXS patterns. **c,** The *f* of PLA fibers fabricated by thermal drawing at various draw-down ratios.

Supplementary Note 16. EDS mapping of MCP fibers

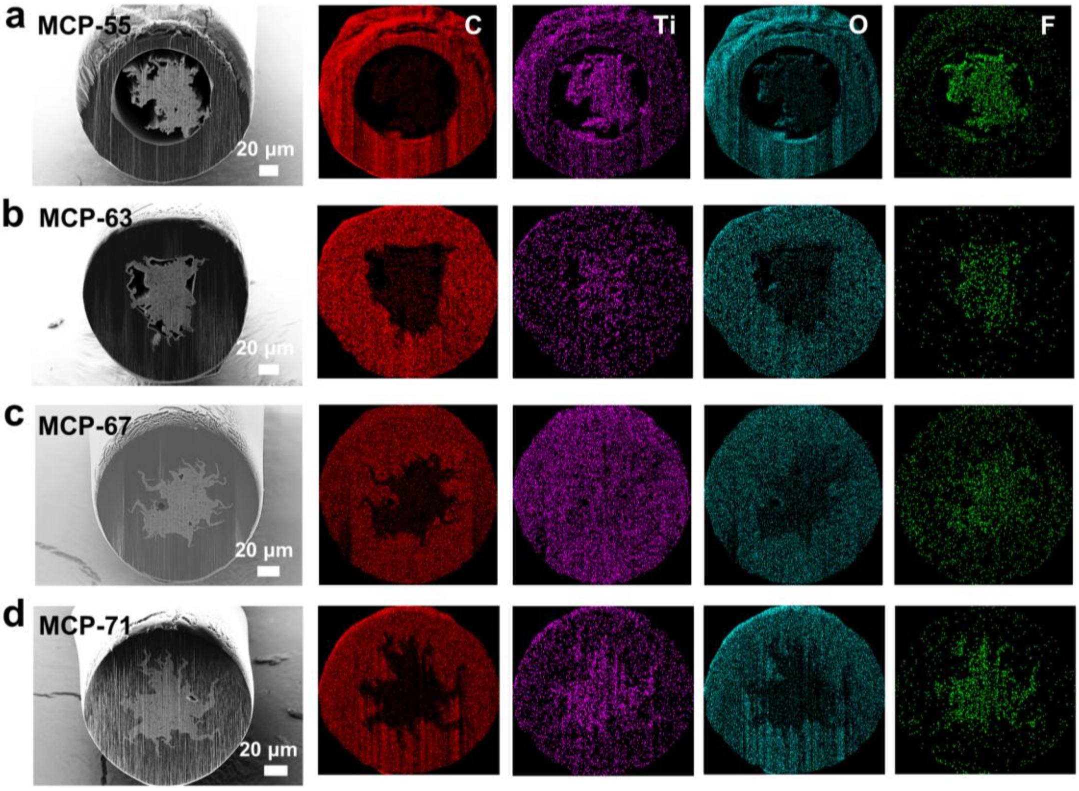

Supplementary Figure 36 | SEM cross-section images and the corresponding EDS mappings of MCP fibers through different draw-down ratios with the EDS mapping. **a,** MCP-55 fiber fabricated by the τ of 55. **b,** MCP-63 fiber fabricated by the τ of 63. **c,** MCP-67 fiber fabricated by the τ of 67. **d,** MCP-71 fiber fabricated by the τ of 71. The results showed that MCP fibers get more compact with the increment of the draw-down ratio from 55 to 71, which significantly reduced the porosity and enhanced the alignment of fibers.

Supplementary Note 17. Mechanical properties of MC fibers fabricated with different weight percentages of CNTs

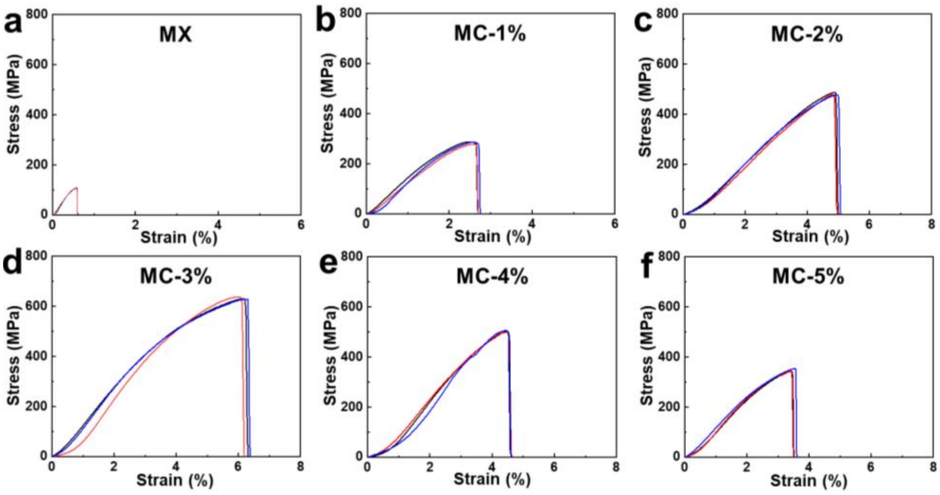

Supplementary Figure 37 | Stress-strain curves for MC fibers fabricated with different weight percentage of short CNTs with a length of ~0.46 μm. **a,** MX fiber. **b,** MC-1% fiber with a weight percentage of 1%. **c,** MC-2% fiber with a weight percentage of 2%. **d,** MC-3% fiber with a weight percentage of 3%. **e,** MC-4% fiber with a weight percentage of 4%. **f,** MC-5% fiber with a weight percentage of 5%.

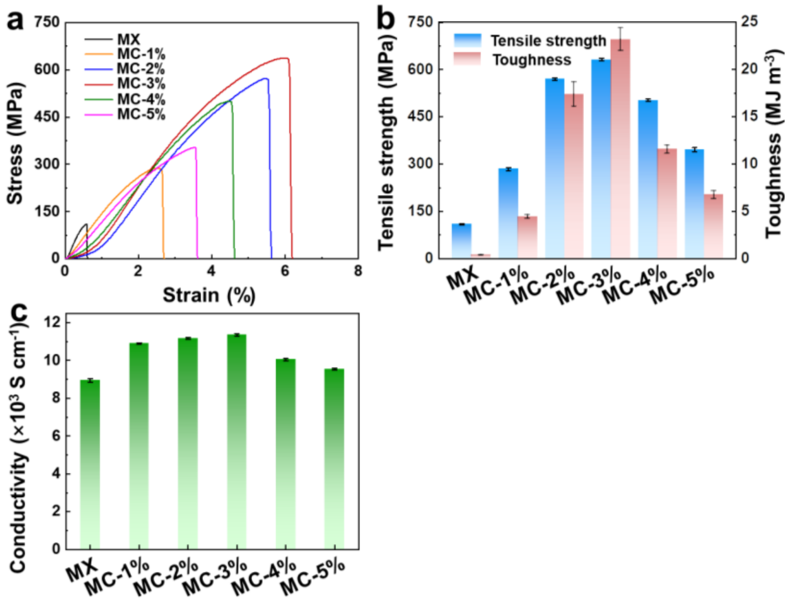

Supplementary Figure 38 | a, Stress-strain curves for MC fibers fabricated with different weight percentages of short CNTs with a length of ~0.46 μm. **b,** Tensile strength and toughness of the MC fibers fabricated with different weight percentages of short CNTs. **c,** Electrical conductivity of the MC fibers fabricated with different weight percentages of short CNTs. All error bars show mean ± SD.

Supplementary Note 18. Mechanical and electrical properties of MCP fibers

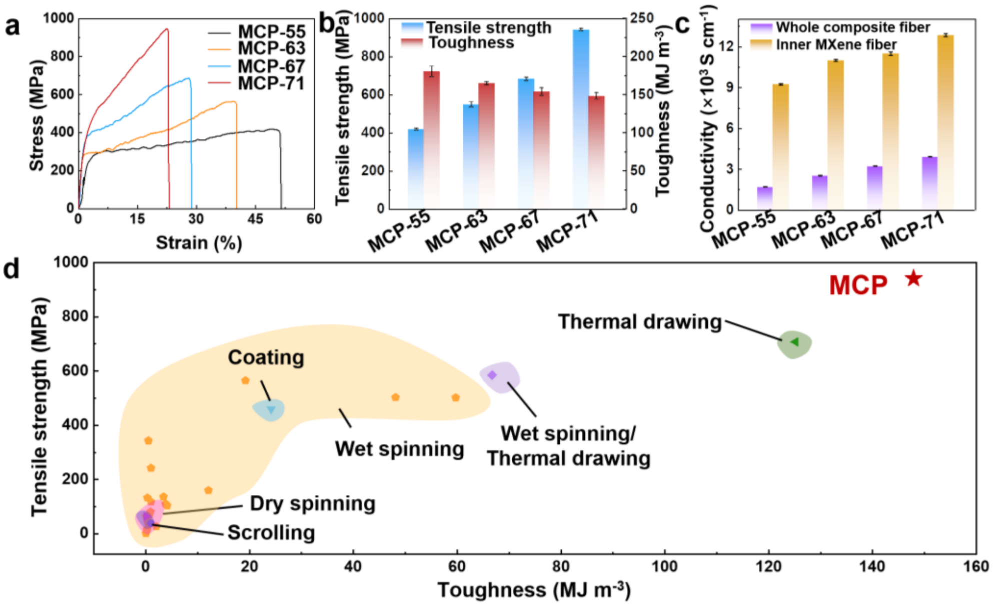

Supplementary Figure 39 | a, The stress-strain curves of the obtained MCP fibers. **b**, The tensile strengths and toughness of fabricated MCP fibers. **c**, The conductivities of fabricated MCP fibers for the whole composite fiber and inner MXene fiber. The electrical conductivity of MCP fibers enhanced at the increasing τ. **d**, Comparison of tensile strength and toughness of the MCP fiber with the reported MXene-based fibers fabricated by various methods of wet and dry spinning, coating, scrolling, wet spinning/thermal drawing, and thermal drawing. All error bars show mean ± SD.

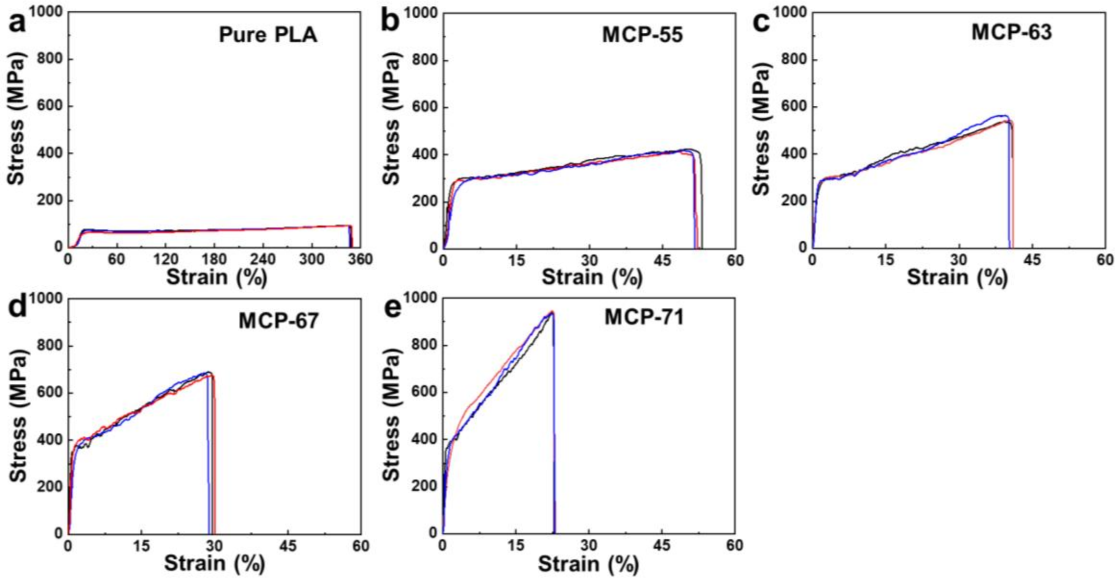

Supplementary Figure 40 | Stress-strain curves for MCP fibers fabricated with different draw-down ratios. **a,** Pure PLA fiber. **b,** MCP-55 fiber. **c,** MCP-63 fiber. **d,** MCP-67 fiber. **e,** MCP-71 fiber.

Supplementary Note 19. Stress-strain curves of loading-unloading cycles for MXene composite fibers

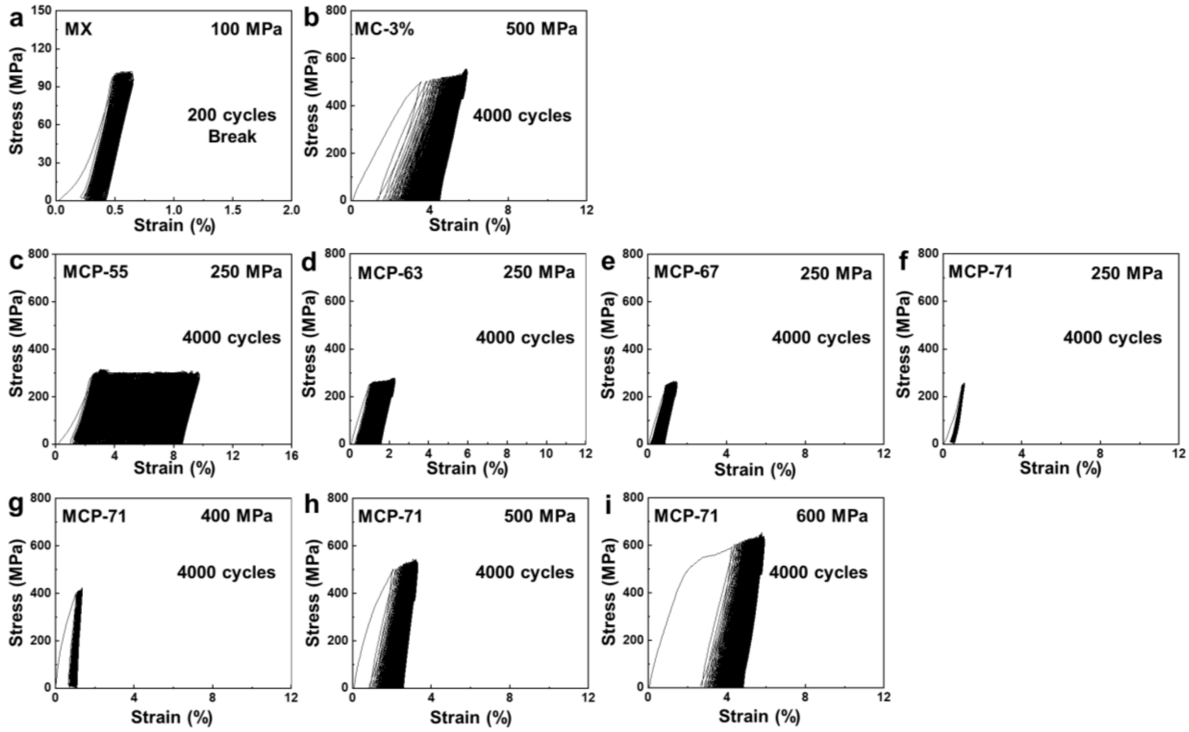

Supplementary Figure 41 | The loading-unloading cyclic mechanical behavior of MXene-based fibers. **a,** The mechanical stress-strain curves of the MX fiber at 200 loading-unloading cycles under the tensile stress of 100 MPa. **b,** The mechanical stress-strain curves of the MC-3% fiber at 4,000 loading-unloading cycles under the tensile stress of 500 MPa. The mechanical stress-strain curves of the MCP fibers with various draw-down ratios at 4,000 loading-unloading cycles under the tensile stress: **c,** MCP-55 under 250 MPa. **d,** MCP-63 under 250 MPa. **e,** MCP-67 under 250 MPa. **f,** MCP-71 under 250 MPa. **g,** MCP-71 under 400 MPa. **h,** MCP-71 under 500 MPa. **i,** MCP-71 under 600 MPa.

Supplementary Note 20. DFT models of four various interfaces

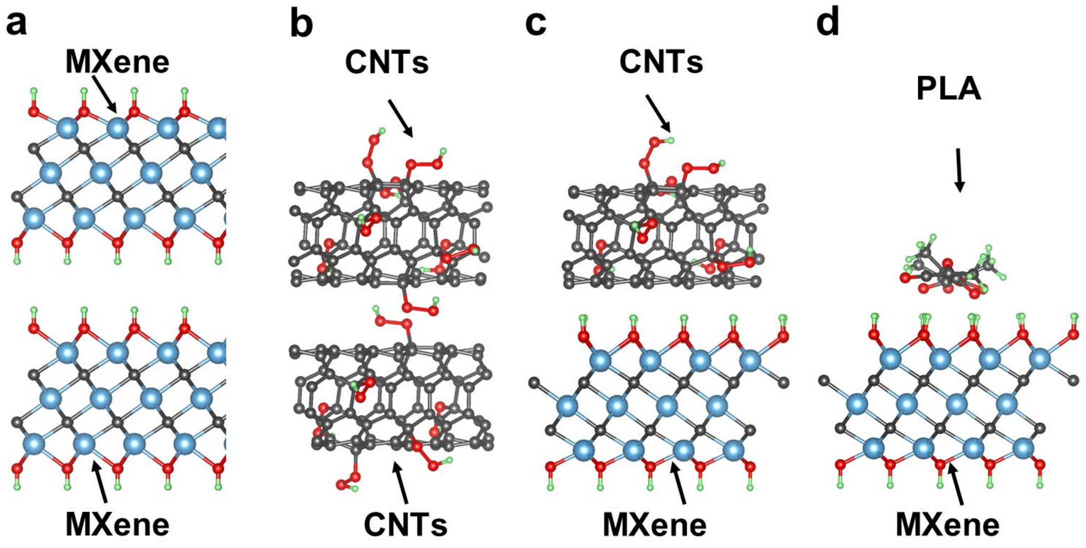

Supplementary Figure 42 | Four kinds of interfaces for DFT calculation, including (**a**) MXene-MXene. (**b**) CNTs-CNTs. (**c**) CNTs-MXene. (**d**) PLA-MXene.

Supplementary Note 21. Fracture mechanism of MCP fiber simulated through FEA

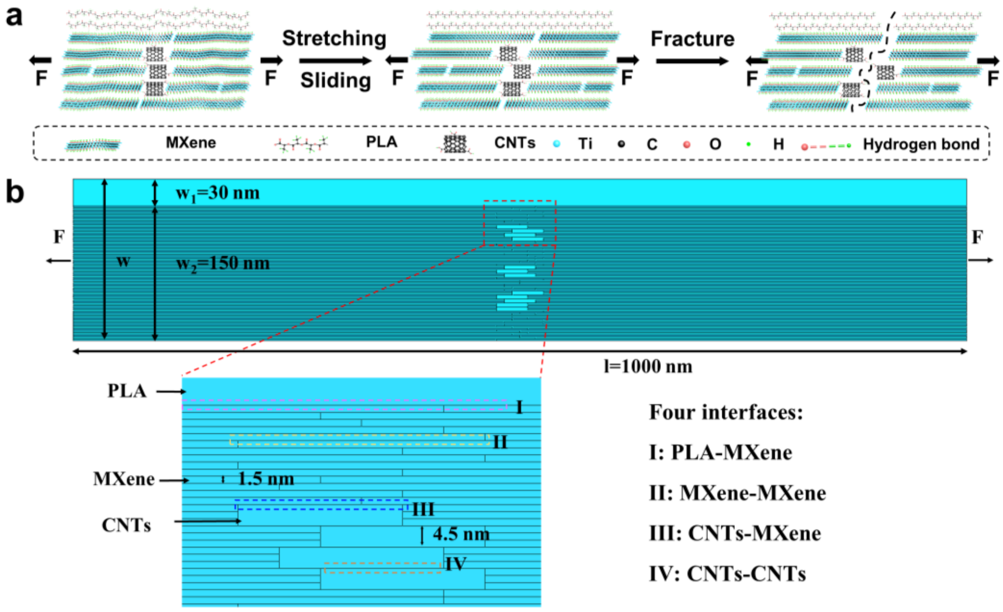

Supplementary Figure 43 | Fracture morphology of the cross-section and FEA model of MCP fiber fabricated through thermal drawing. a, Fracture cartoon schematic diagram of MCP fiber, as well as sliding between MXene nanosheets and failure of the hydrogen bonds of CNTs-MXene and PLA-MXene interfaces. **b**, FEA model with a length (l) of 1,000 nm and width (w) of 180 nm was developed to further reveal the fracture mechanism of MCP fibers. Four kinds of cohesive elements including PLA-MXene (I), MXene-MXene (II), CNTs-

MXene (III), and CNTs-CNTs (IV) represent the four kinds of interfacial interactions, while the MXene nanosheets have a thickness of 1.5 nm and the short CNTs have a diameter of 4.5 nm according to that of the experiments.

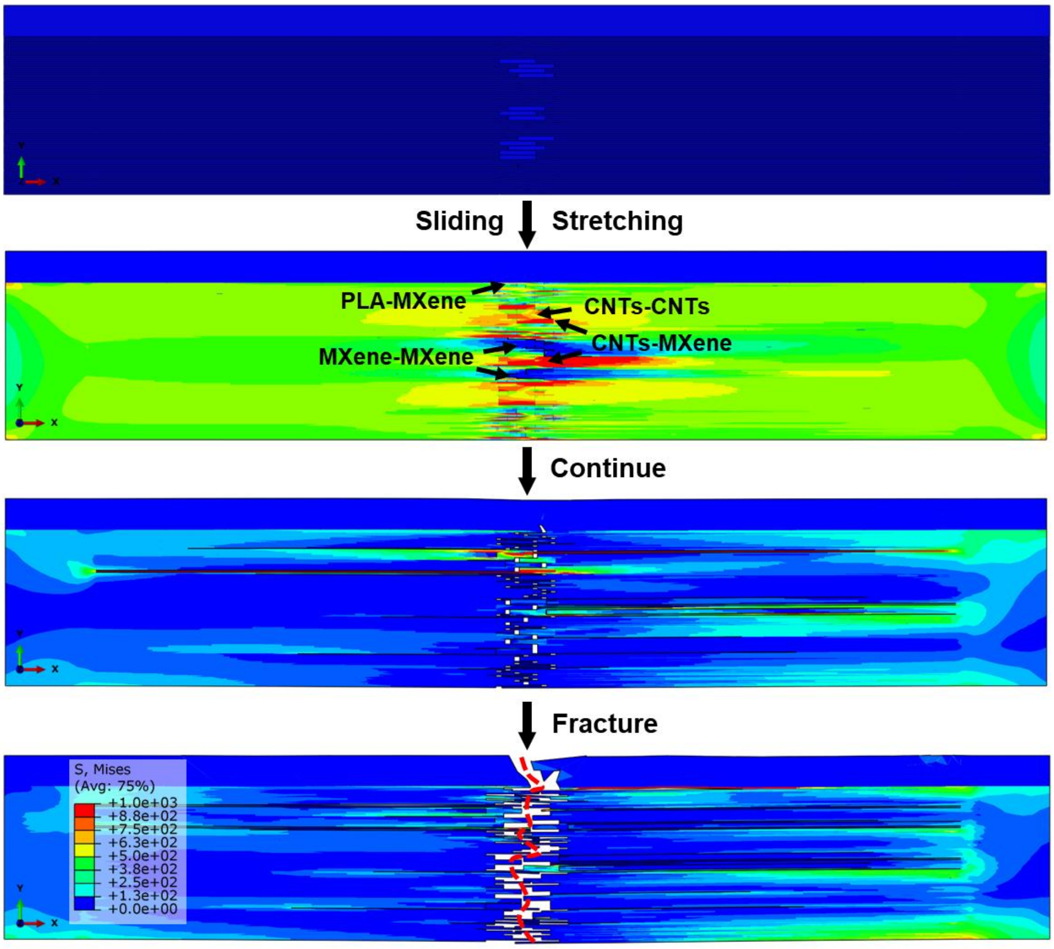

Supplementary Figure 44 | Schematic diagram of the fracture mechanism of MCP fiber according to the FEA simulation.

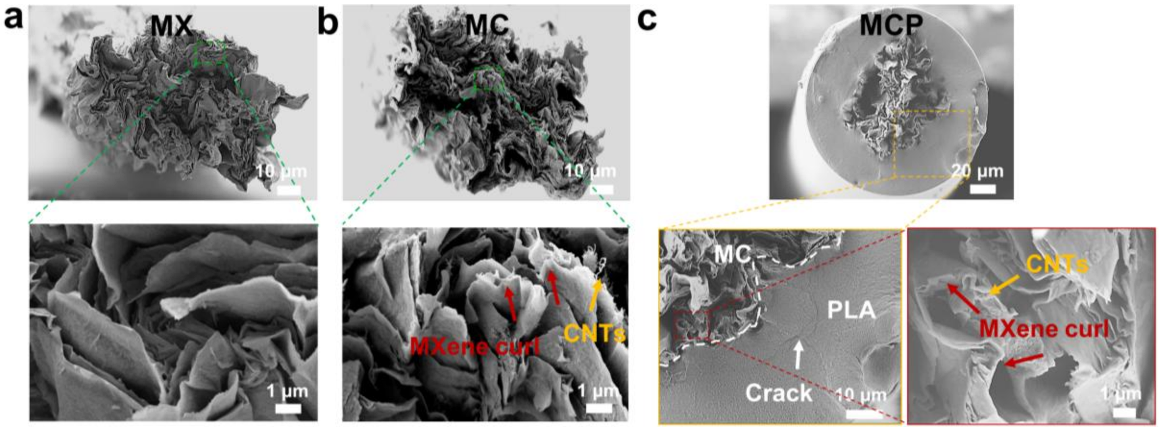

Supplementary Figure 45 | Fracture morphology of the MXene composite fibers, including

MX (**a**), MC (**b**), and MCP (**c**) fibers. The results indicated the pull-out of short CNTs and the crack of the encapsulated PLA layer at the interface between MXene nanosheets and PLA.

Supplementary Note 22. Photographs of the smart textiles

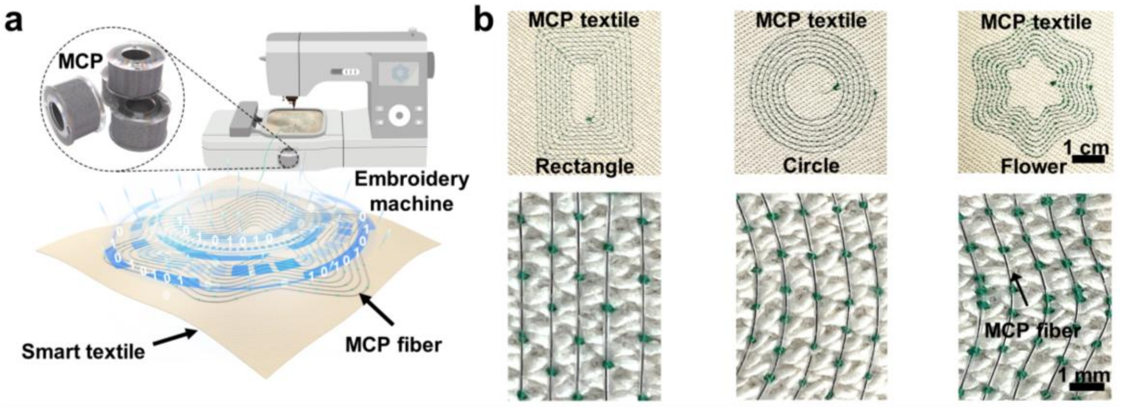

Supplementary Figure 46 | a, The embroidery machine method to fabricate the smart textiles using the MCP fiber. **b**, MCP textiles with rectangle, circle, and flower patterns.

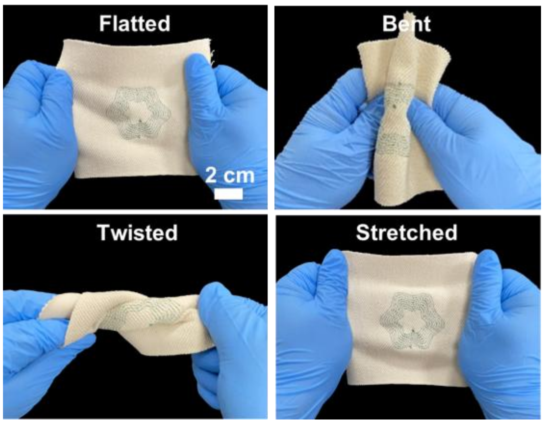

Supplementary Figure 47 | Photographs of the smart textile when flattened, bent, twisted, and stretched.

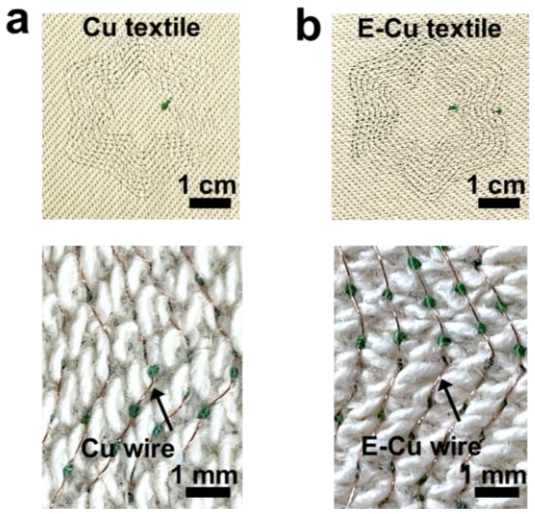

Supplementary Figure 48 | Flower smart textiles fabricated on Cu wire (**a**) and E-Cu wire (**b**).

Supplementary Note 23. Durability of smart textiles

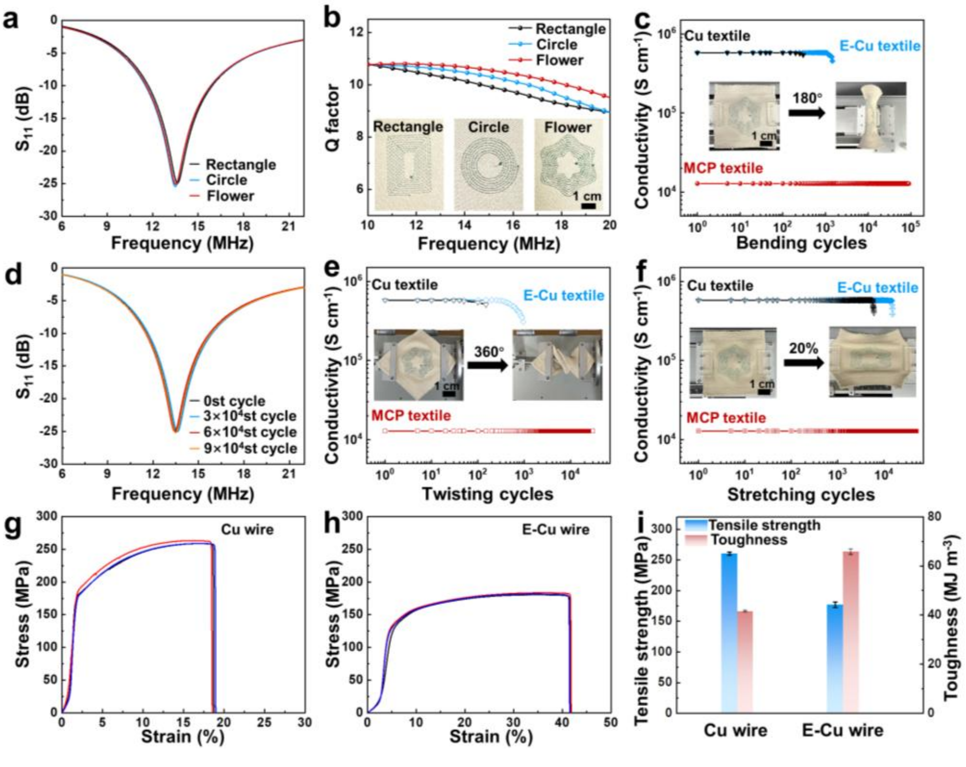

Supplementary Figure 49 | Mechanical durability of the smart textiles. S_{11} (**a**) and Q (**b**) of MCP textiles with rectangle, circle, and flower patterns. **c**, The mechanical durability of Cu, E-Cu, and MCP textiles subjected to 180° bending cycles. **d**, S_{11} curves during 9×10^4 cycles of bending for MCP textiles with flower pattern. The mechanical durability of Cu, E-Cu, and MCP textiles subjected to 360° twisting cycles (**e**) and stretching cycles at 20% strain (**f**). Stress-strain curves of Cu wire (**g**) and E-Cu wire (**h**). **i**, Tensile strength and toughness of Cu wire and E-Cu wire. All error bars show mean ± SD.

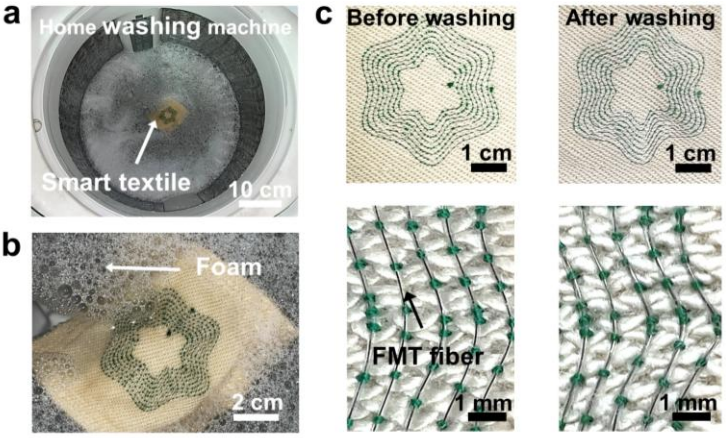

Supplementary Figure 50 | a, and **b**, Smart textile with the flower pattern for home washing. **c**, Photograph of the MCP textile with flower pattern before washing and after 90 cycles of washing.

Supplementary Note 24. The electromagnetic performance of the textiles at 13.56 MHz

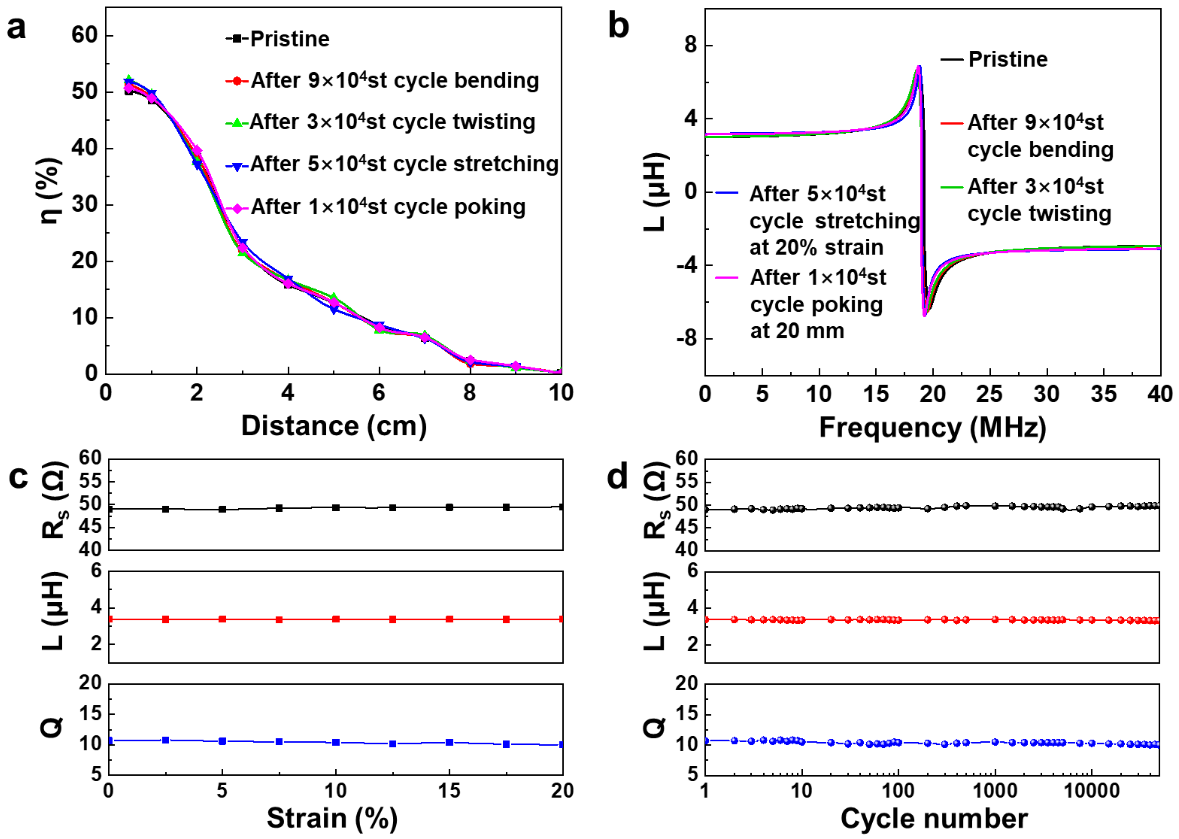

Supplementary Figure 51 | Durability of the MCP spiral inductor textile. **a**, Power-transfer efficiency (η) of the MCP spiral inductor textile, even after enduring 9×10^4 bending cycles at a 180° angle, 3×10^4 twisting cycles, 5×10^4 stretching cycles under 20% strain, and 1×10^4

poking cycles with displacement ranging from 0 mm to 20 mm. **b**, Inductance of the MCP spiral inductor textile, even after enduring 9×10^4 bending cycles at a 180° angle, 3×10^4 twisting cycles, 5×10^4 stretching cycles under 20% strain, and 1×10^4 poking cycles with displacement ranging from 0 mm to 20 mm. **c**, Comparison of the measured resistance (Rs), inductance (L), and quality factor (Q) of the MCP spiral inductor textile under 0-20% strain at 13.56 MHz. **d**, Stability assessment of the MCP spiral inductor textile, subjected to over 5×10^4 cycles under a 20% strain.

Supplementary Note 25. The MVTR of the smart textiles

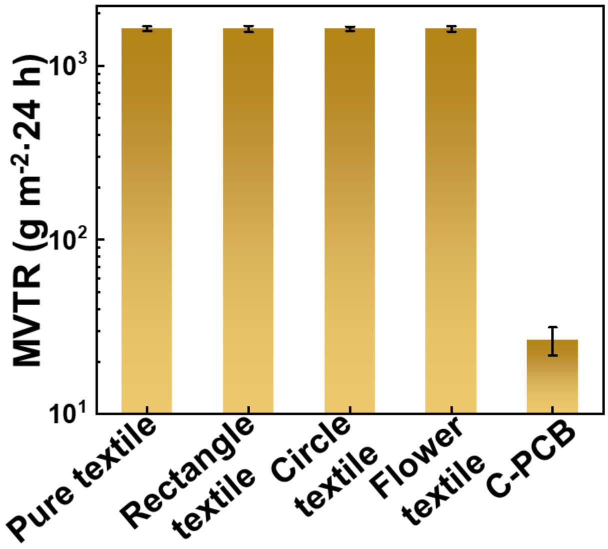

Supplementary Figure 52 | The MVTR of the pure textile without MCP fiber, and smart textile embroidered with different patterns using MCP fiber, including rectangle, circle, and flower, compared to that of the rigid C-PCB. All error bars show mean ± SD.

Supplementary Note 26. The performance of the power unit based on smart textiles

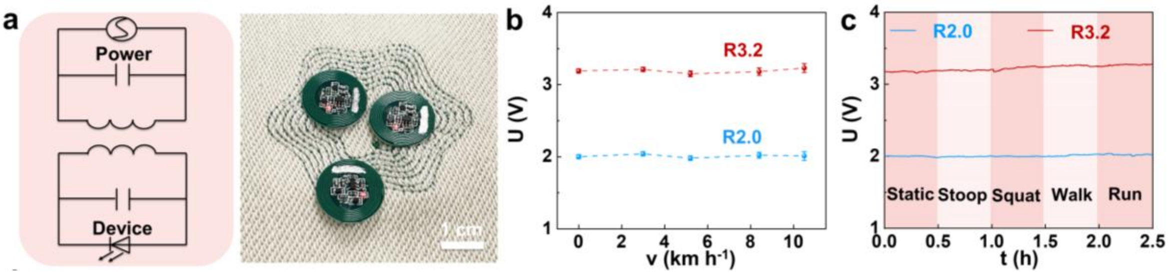

Supplementary Figure 53 | a, The block diagram of the wireless power unit and photography of powering light-emitting devices. The received voltage at different v (**b**) from 0 km h^{-1} to 10.5 km h^{-1} and human being's condition (**c**) when output from the smart textile in wireless power unit.

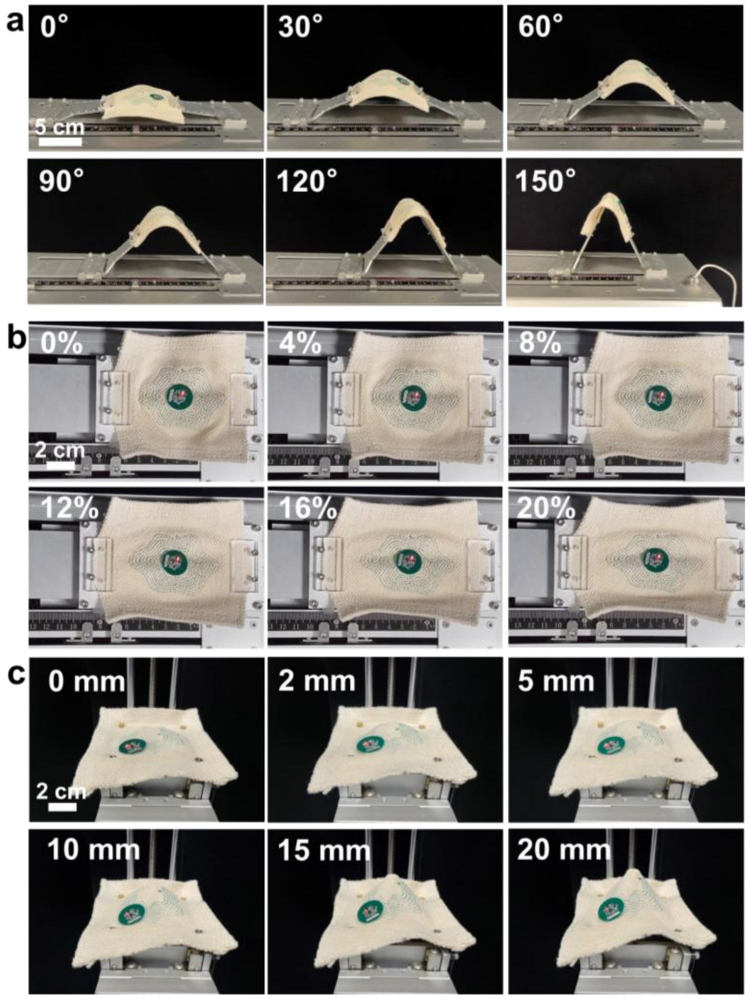

Supplementary Figure 54 | Durability of the wireless power unit in the smart textile utilizing MCP fibers. (**a**) Performance under bending angles ranging from 0° to 150°. (**b**) Stretching under strains varying from 0% to 20%. (**c**) Resistance to poking with distances from 0 mm to 20 mm.

Supplementary Note 27. Schematic block diagram of the designed chip

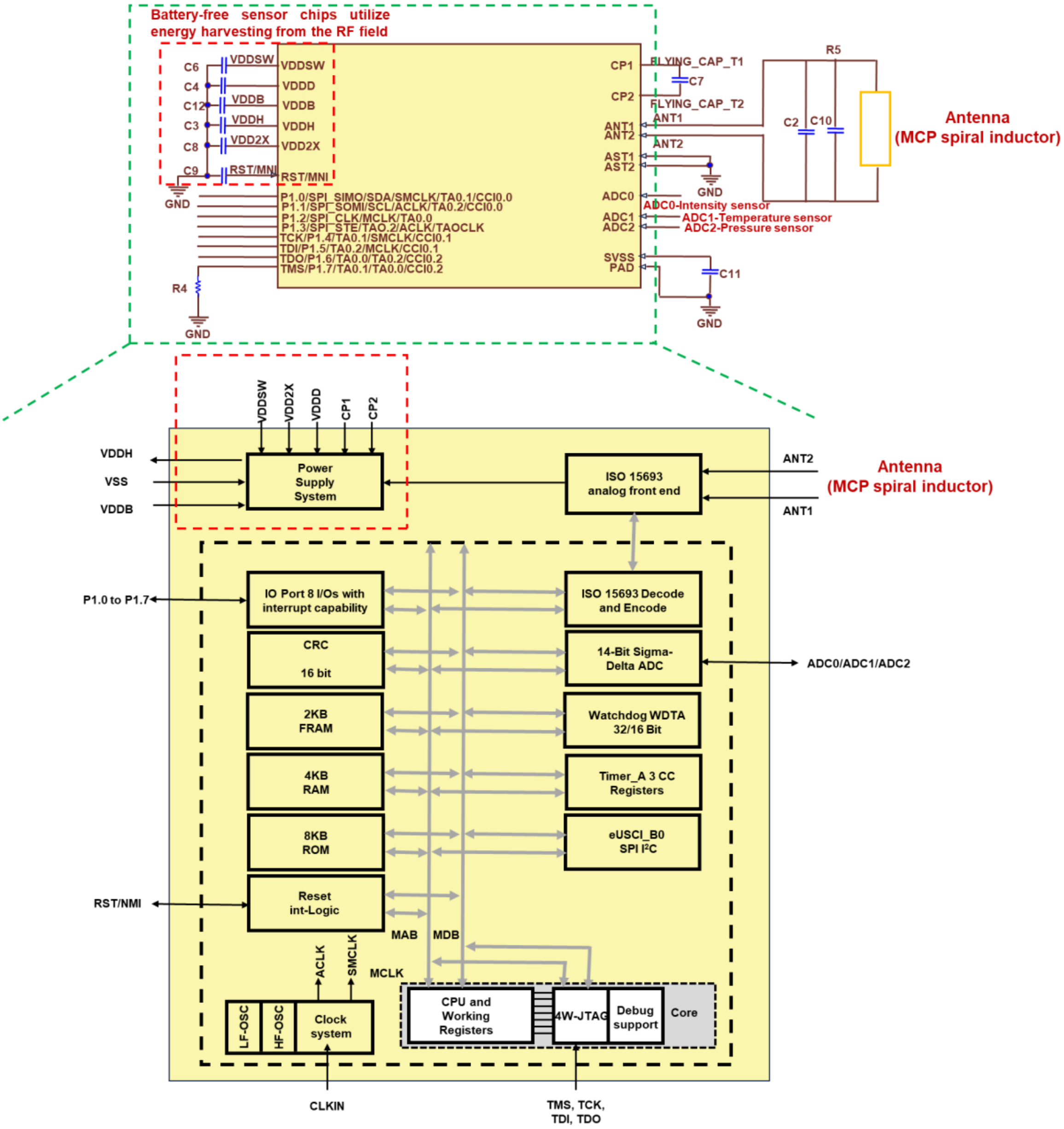

Supplementary Figure 55 | Schematic block diagram of the designed chip within the sensing unit.

Supplementary Note 28. The mechanism of long-range, battery-free wireless human health monitoring system

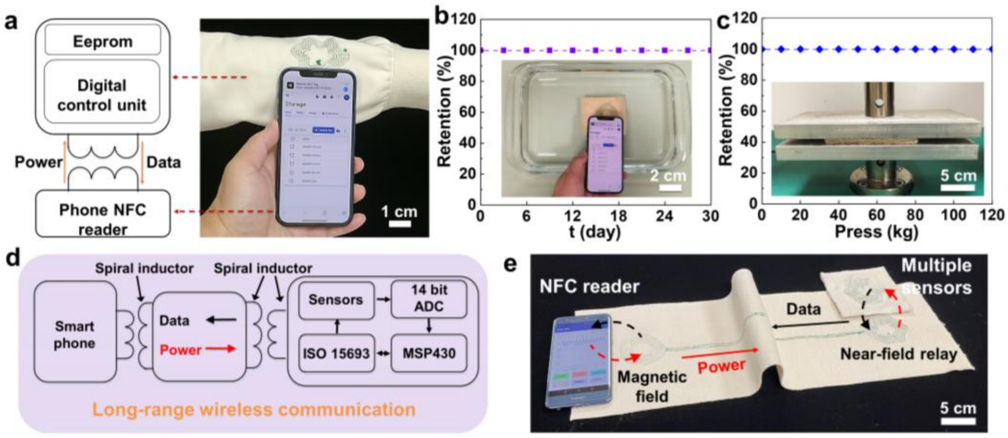

Supplementary Figure 56 | Long-range, battery-free wireless human health monitoring system. a, Photograph of reading the database when transferred from the sensing unit with the block diagram of the storage unit. Wireless sensing unit assembled from MCP textile. **b**, The retention of the storage unit performance when being immersed in water for 30 days. **c**, The retention of the storage unit performance under a pressing load ranging from 0 kg to 120 kg. **d**, The block diagram for long-range wireless communication. **e**, Photography of long-range, battery-free wireless human health monitoring system assembled from MCP textile a relay for a long-range (~50 cm) wireless communication.

Supplementary Note 29. Temperature durability of the wireless communication system

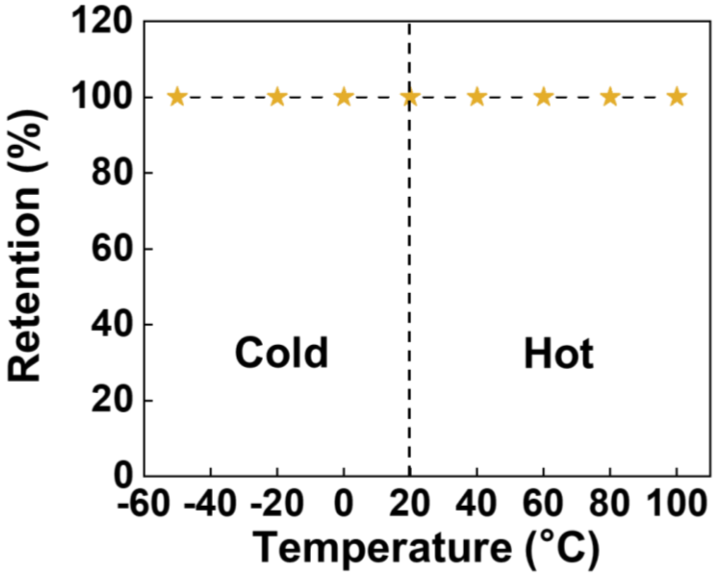

Supplementary Figure 57 | Retention of the entire wireless system under the working

temperature range of -50 °C to 100 °C.

Supplementary Note 30. Battery-free, body-coupled wireless textiles

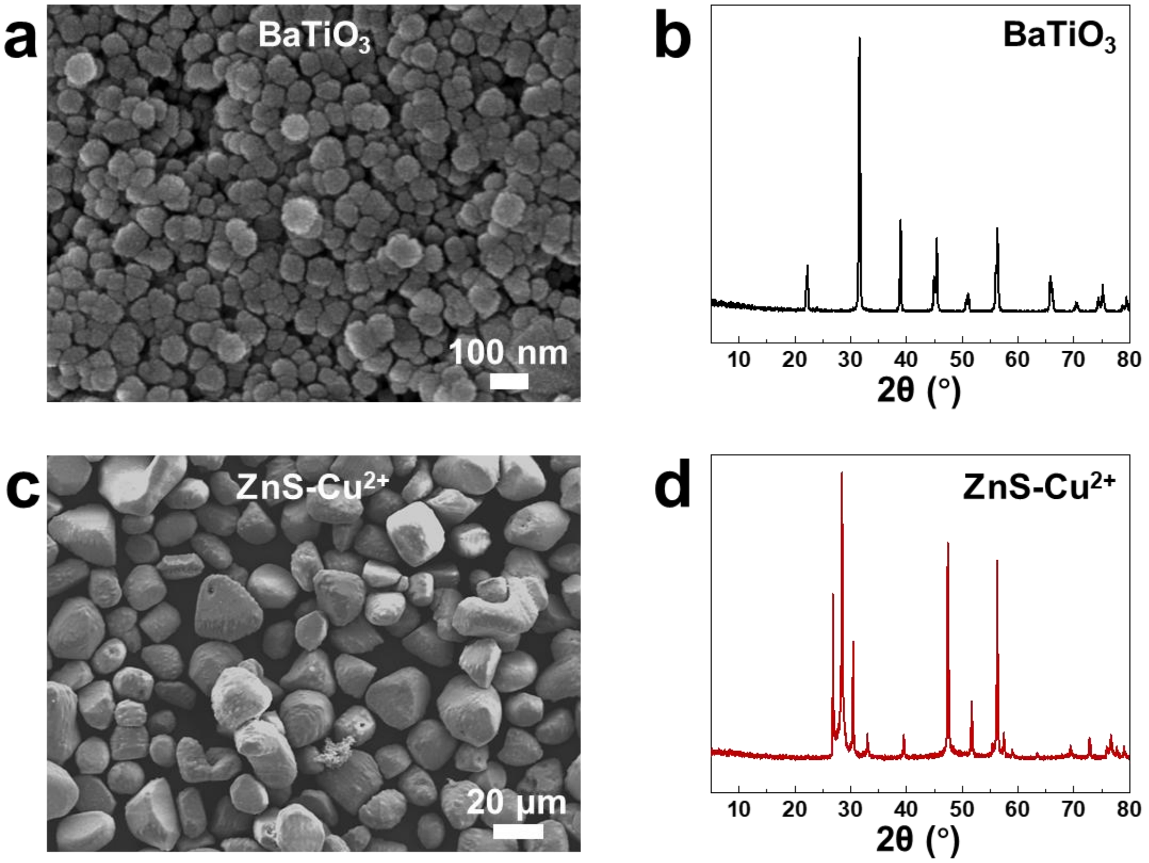

Supplementary Figure 58 | a, SEM image of $BaTiO_3$ nanoparticles. **b**, XRD pattern of $BaTiO_3$ nanoparticles. **c**, SEM image of ZnS-Cu^{2+} particles. **d**, XRD pattern of ZnS-Cu^{2+} particles.

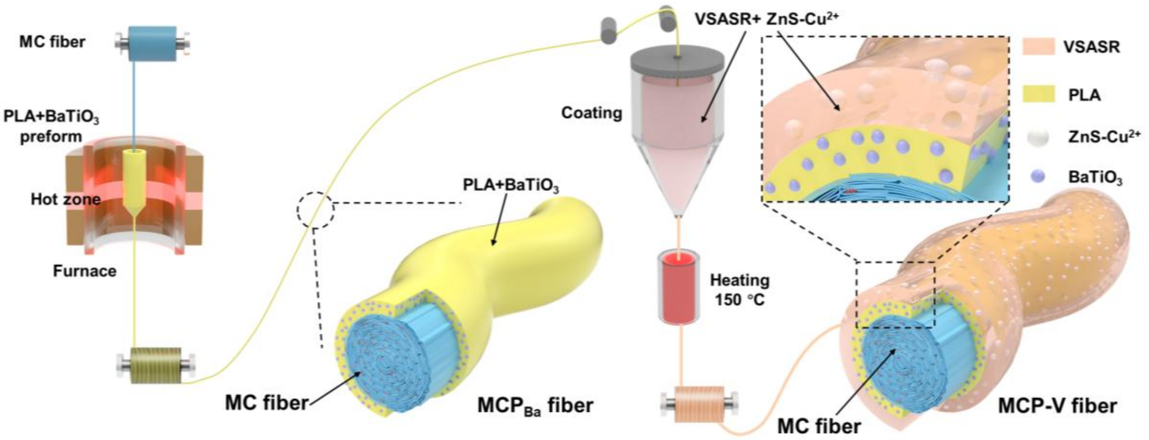

Supplementary Figure 59 | Fabrication of MCP-V fiber through thermal drawing and coating based on the MC fiber.

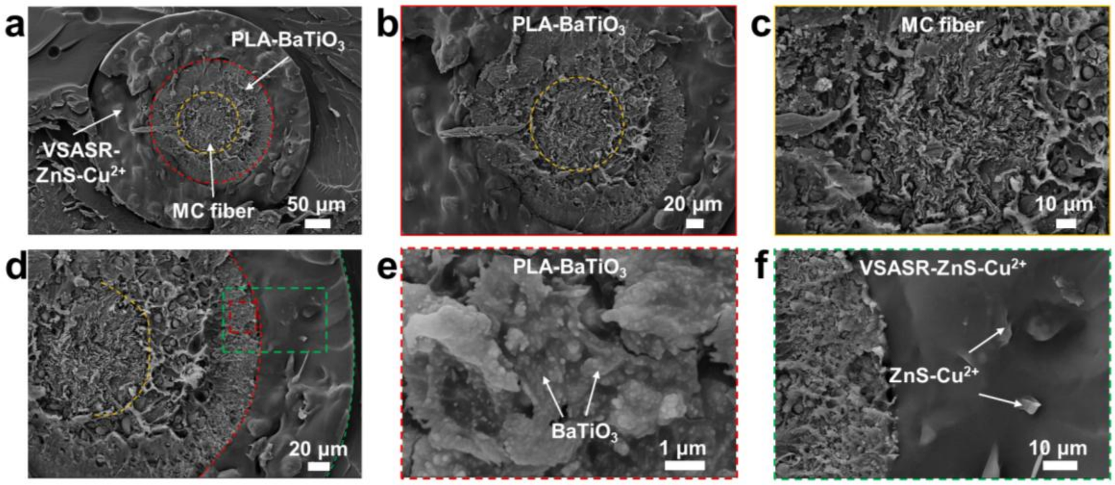

Supplementary Figure 60 | SEM image of the cross-section of the MCP-V fiber. a, Cross-section of the MCP-V fiber. **b**, PLA-$BaTiO_3$ layer in MCP-V fiber. **c**, Inner MC fiber in MCP-V fiber. **d**, Three layers of the MCP-V fiber, including inner MC fiber, PLA-$BaTiO_3$ layer, and VSASR-ZnS-Cu^{2+}. **e**, The distribution of the $BaTiO_3$ in the PLA-$BaTiO_3$ layer of MCP-V fiber. **f**, The distribution of the ZnS-Cu^{2+} in the VSASR-ZnS-Cu^{2+} layer of MCP-V fiber.

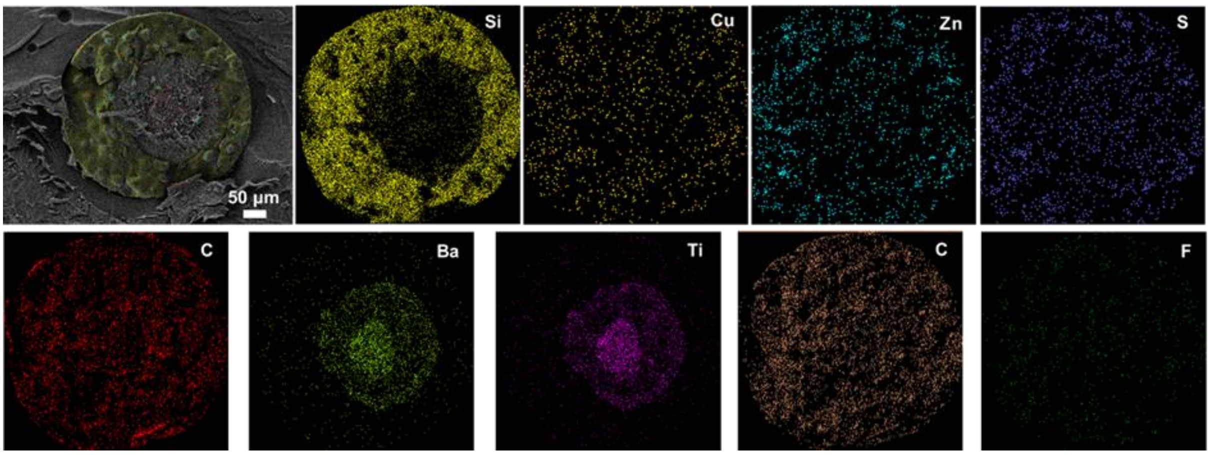

Supplementary Figure 61 | SEM images and the corresponding energy-dispersive X-ray spectroscopy (EDS) mappings of the cross-sections of MCP-V fibers.

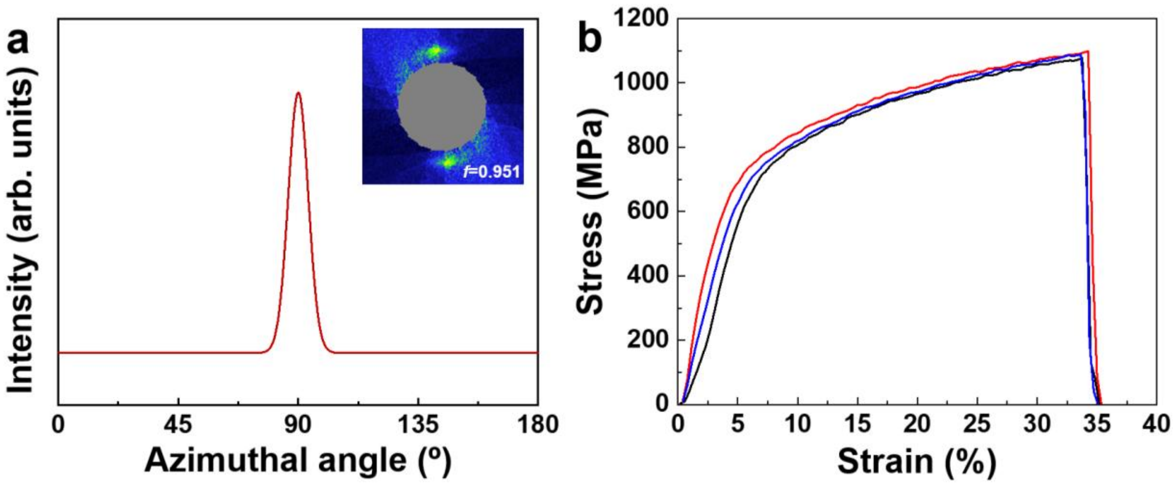

Supplementary Figure 62 | a, Plots of azimuthal angle of the MCP-V fiber with the WAXS patterns. **b**, Stress-strain curves of the MCP-V fiber.

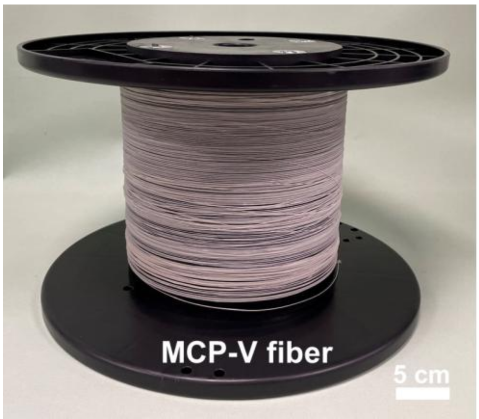

Supplementary Figure 63 | Photograph of the reel holding the MCP-V fiber, extending hundreds of meters in length.

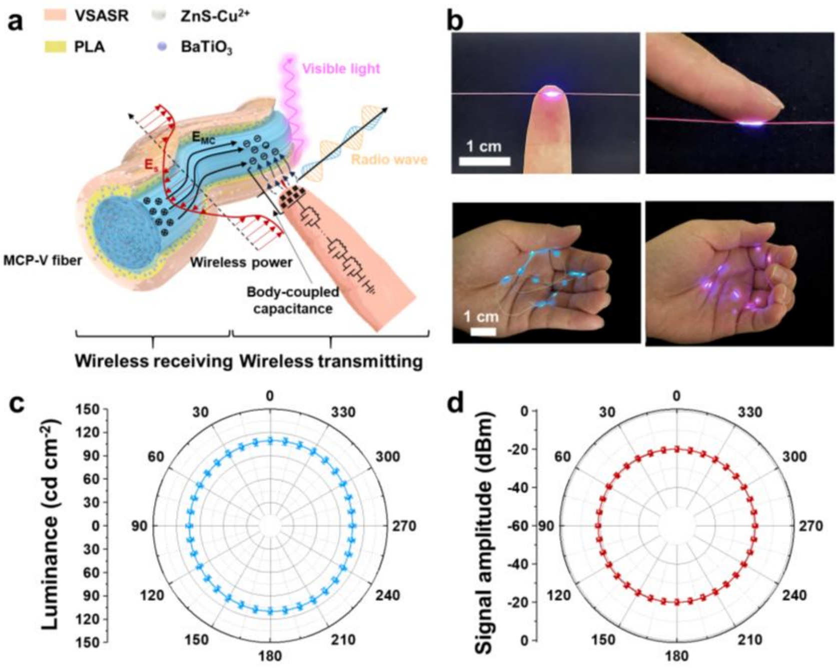

Supplementary Figure 64 | a, Illustration of the working principle of body-coupled MCP-V fiber. Where E_{MC} represents the induced electric field within the MC fiber antenna, and E_s denotes the EM field acting on the MCP-V fiber's surface. **b**, The photograph shows the MCP-C fiber being wirelessly powered by the finger, while also capturing the surrounding electromagnetic energy. **c**, Directional diagrams illustrating wireless optical luminance. **d**, Directional diagrams depicting wireless electrical signals. Data are presented based on three repeated measurements (mean ± SD).

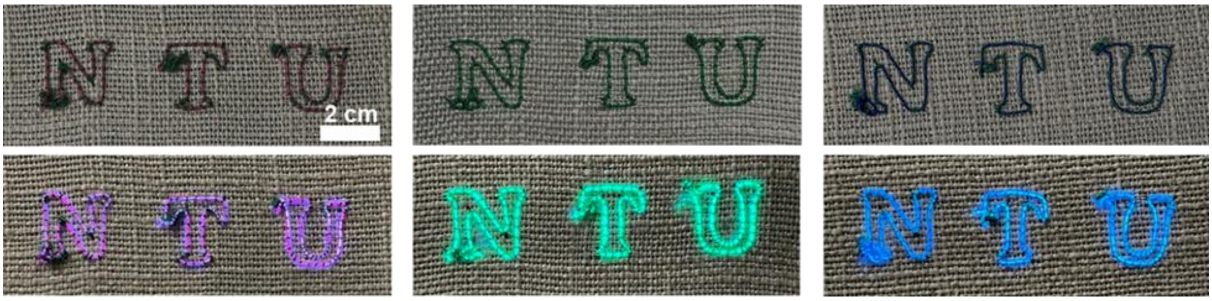

Supplementary Figure 65 | Photographs of the dyed MCP-V fibers emit a red, green, and blue glow when exposed to body-coupled EM fields for the assisted communication.

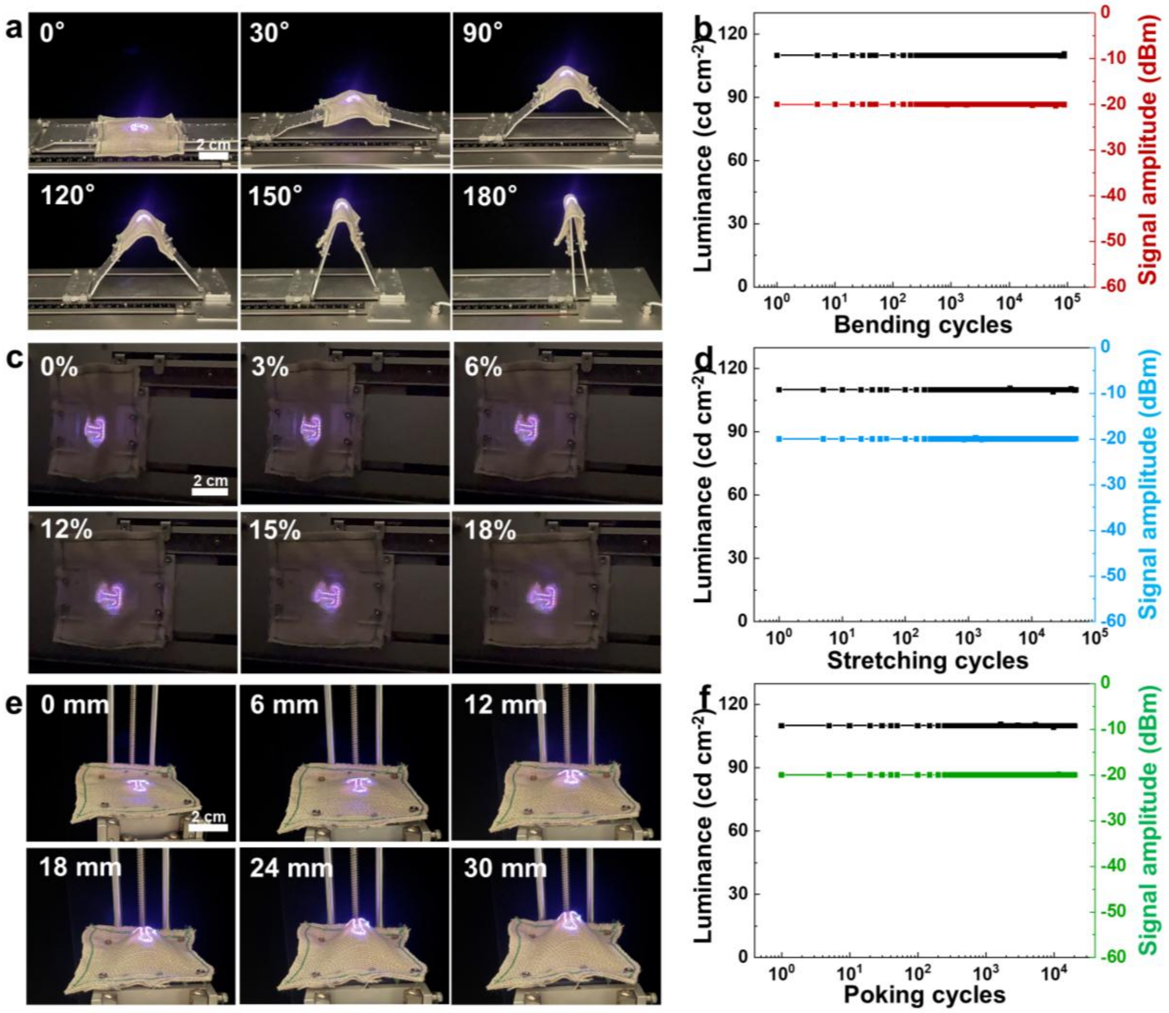

Supplementary Figure 66 | Durability of the MCP-V Textile. (**a**) and (**b**) Performance of wireless optical and electrical signals under bending angles ranging from 0° to 180° and after repeated cycles. (**c**) and (**d**) Stretching behavior under strains varying from 0% to 18% and after repeated cycles. (**e**) and (**f**) Resistance to puncture under poking distances from 0 mm to 30 mm and after repeated cycles.

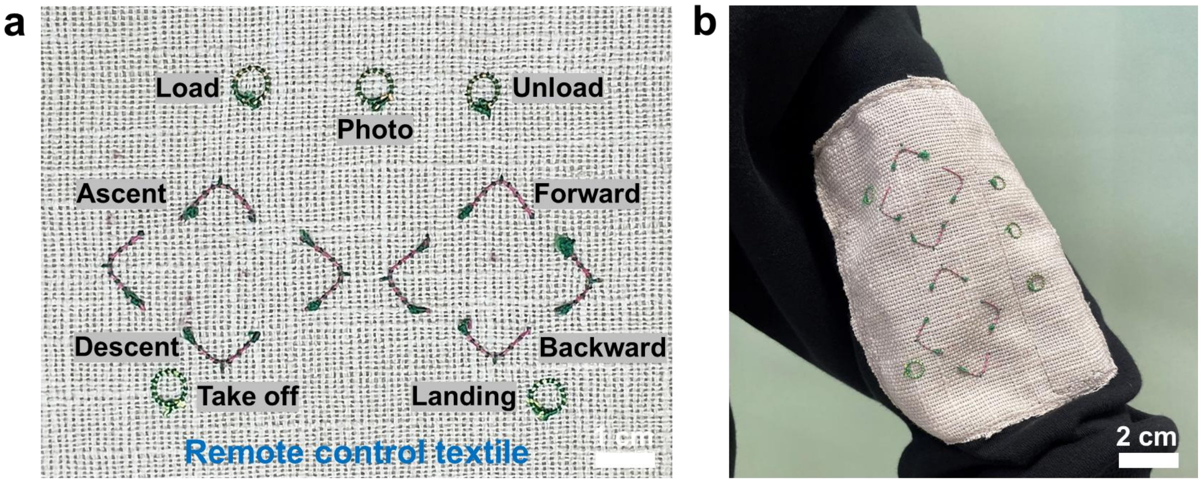

Supplementary Figure 67 | a, Photograph of the MCP-V textile used for drone remote control, showcasing functionalities such as takeoff, landing, ascent, descent, forward and backward movement, as well as load and unload. **b**, Photograph of a drone remote control design embroidered on a hoodie. The buttons in the MCP-V textile were embroidered with MCP-V fibers of varying lengths. As a result, the buttons can emit radio electromagnetic (EM) wave signals with different intensities. Each signal intensity is mapped to a specific control function button.

Supplementary Tables

Supplementary Table 1 | The *f*, density, and porosity of fabricated MC fibers with different lengths of CNTs. All error bars show mean ± SD.

Samples	Orientation order (*f*)	*d*-spacing (Å)	Density (ρ) (g cm^{-3})	Porosity (%)
**MC-13.91**	0.773	13.81	2.65 ± 0.10	31.0 ± 2.6
**MC-7.98**	0.795	13.17	2.91 ± 0.06	27.7 ± 1.5
**MX**	0.831	13.02	3.15 ± 0.10	22.7 ± 2.5
**MC-2.55**	0.839	12.98	3.23 ± 0.10	21.0 ± 2.5
**MC-0.46**	0.848	12.96	3.45 ± 0.08	15.7 ± 2.0
**MC-0.19**	0.842	12.97	3.32 ± 0.12	18.8 ± 2.9

Supplementary Table 2 | The tensile strength, Young's modulus, strain, and toughness of the fabricated MC fibers with different lengths of CNTs. All error bars show mean ± SD.

Samples	S (μm^2) Whole fiber	Tensile strength (MPa)	Young's modulus (GPa)	Strain (%)	Toughness (MJ m^{-3})
**MC-13.91**	5,749.7 ± 112.5	50.4 ± 1.0	14.9 ± 0.3	0.50 ± 0.01	0.14 ± 0.01
**MC-7.98**	5,377.3 ± 99.5	93.2 ± 2.0	18.5 ± 0.1	0.56 ± 0.01	0.29 ± 0.01
**MX**	5,754.9 ± 73.9	109.3 ± 2.2	21.5 ± 0.1	0.58 ± 0.01	0.36 ± 0.02
**MC-2.55**	5,181.8 ± 88.5	182.5 ± 1.2	24.1 ± 0.2	1.87 ± 0.04	1.96 ± 0.10
**MC-0.46**	5,116.8 ± 68.8	284.3 ± 4.7	32.8 ± 0.3	2.56 ± 0.03	4.42 ± 0.20
**MC-0.19**	5,017.0 ± 96.8	225.0 ± 0.4	26.2 ± 0.2	2.26 ± 0.03	2.96 ± 0.05

Supplementary Table 3 | The electrical conductivity of the fabricated MC fibers with different lengths of CNTs. All error bars show mean ± SD.

Samples	Conductivity (S cm^{-1})
**MC-13.91**	8,173.2 ± 106.45
**MC-7.98**	8,633.8 ± 87.24
**MX**	8,944.1 ± 85.76
**MC-2.55**	9,142.4 ± 76.88
**MC-0.46**	9,597.3 ± 40.68
**MC-0.19**	9,337.8 ±76.54

Supplementary Table 4 | The weight percentages of CNTs and MXene in MC fibers calculated according to the TGA curves.

Samples	MXene (wt%)	CNTs (wt%) added in the experiment	CNTs (wt%) calculated according to the TGA
**MX**	100	0	0
**MC-1%**	98.9	1	1.1
**MC-2%**	97.7	2	2.3
**MC-3%**	96.8	3	3.2
**MC-4%**	95.6	4	4.4
**MC-5%**	94.3	5	5.6

Supplementary Table 5 | The *f*, density, and porosity of the fabricated MC fibers fabricated with different weight percentages of CNTs. All error bars show mean ± SD.

Samples	Orientation factor (*f*)	*d*-spacing (Å)	Density (ρ) (g cm^{-3})	Porosity (%)
**MX**	0.831	13.02	3.15 ± 0.10	22.7 ± 2.5
**MC-1%**	0.848	12.96	3.45 ± 0.08	15.7 ± 2.0
**MC-2%**	0.882	12.82	3.79 ± 0.11	8.4 ± 2.7
**MC-3%**	0.915	12.76	3.90 ± 0.05	6.2 ± 1.2
**MC-4%**	0.866	12.85	3.71 ± 0.06	10.1 ± 1.5
**MC-5%**	0.854	12.92	3.52 ± 0.12	14.3 ± 2.9

Supplementary Table 6 | The f, density, and porosity of MC fibers fabricated through different spinning nozzle with diameters from 180 μm to 500 μm. All error bars show mean ± SD.

Samples	Orientation factor (f)	*d*-spacing (Å)	Density (ρ) (g cm^{-3})	Porosity (%)
**MC-180**	0.953	11.32	4.50 ± 0.12	4.0 ± 1.1
**MC-250**	0.931	11.65	4.32 ± 0.09	5.1 ± 0.5
**MC-380**	0.915	12.76	3.90 ± 0.05	6.2 ± 1.2
**MC-500**	0.872	12.92	3.68 ± 0.06	10.4 ± 1.4

Supplementary Table 7 | The tensile strength and toughness of the MC fibers fabricated through different spinning nozzles with diameters from 180 μm to 500 μm. All error bars show mean ± SD.

Samples	S (μm^2) Whole fiber	Ultimate tensile force (N)	Tensile strength (MPa)	Strain (%)	Toughness (MJ m^{-3})
**MC-180**	1,152.6 ± 64.3	1.36 ± 0.01	1,056.0 ± 5.8	8.60 ± 0.12	45.9 ± 2.7
**MC-250**	2,284.7 ± 86.5	1.98 ± 0.02	822.0 ± 6.5	7.54 ± 0.29	32.7 ± 1.0
**MC-380**	5,139.2 ± 104.6	4.87 ± 0.03	631.8 ± 4.5	6.14 ± 0.17	23.2 ± 1.2
**MC-500**	1,3780.7 ± 96.7	10.44 ± 0.08	507.0 ± 4.1	5.00 ± 0.03	11.7 ± 0.4

Supplementary Table 8 | The electrical conductivity of the fabricated MC fibers fabricated through different spinning nozzle with diameters from 180 μm to 500 μm. All error bars show mean ± SD.

Samples	Conductivity (S cm^{-1})
**MC-180**	14,589.5 ± 95.6
**MC-250**	12,768.4 ± 86.7
**MC-380**	11,364.8 ± 65.8
**MC-500**	10,679.5 ± 76.8

Supplementary Table 9 | The density and porosity of MC fibers under heating from 25 °C to 210 °C. All error bars show mean ± SD.

**Temperature (°C)**	***d*-spacing (Å)**	**Density (ρ) (g cm^{-3})**	**Porosity (%)**
**25**	12.76	3.90 ± 0.05	6.2 ± 1.2
**100**	12.56	3.88 ± 0.06	8.1 ± 1.4
**150**	12.14	3.83 ± 0.12	12.3 ± 2.8
**180**	12.03	3.80 ± 0.10	13.8 ± 2.3
**210**	12.02	3.79 ± 0.08	14.1 ± 1.8

Supplementary Table 10 | The precise composition of the MCP fibers.

**Samples**	**MXene (wt%)**	**CNTs (wt%)**	**PLA (wt%)**
**MCP-55**	43.5	1.6	54.9
**MCP-63**	49.2	1.8	49.0
**MCP-67**	55.6	2.0	42.4
**MCP-71**	59.6	2.2	38.2

Supplementary Table 11 | The density and porosity of the fabricated MCP fibers with increasing draw-down ratios. All error bars show mean ± SD.

**Samples**	***d*-spacing (Å)**	**Density (ρ) (Inner MC fiber) (g cm^{-3})**	**Density (ρ) (MCP) (g cm^{-3})**	**Porosity (Inner MC fiber) (%)**
**MC-3%**	12.76	3.90 ± 0.05	-	6.2 ± 1.2
**MCP-55**	11.98	3.81 ± 0.1	1.43 ± 0.16	13.9 ± 2.3
**MCP-63**	11.80	4.10 ± 0.04	1.68 ± 0.10	8.8 ± 0.9
**MCP-67**	11.70	4.30 ± 0.05	1.88 ± 0.08	5.2 ± 1.1
**MCP-71**	11.55	4.40 ± 0.03	1.97 ± 0.11	4.2 ± 0.7

Supplementary Table 12 | The *f* of the fabricated MCP fibers with increasing draw-down ratios.

Samples	Orientation factor (Inner MXene fiber) (*f*)
**MC-3%**	0.915
**MCP-55**	0.895
**MCP-63**	0.908
**MCP-67**	0.926
**MCP-71**	0.945

Supplementary Table 13 | The diameters of the obtained MCP and inner MC fibers fabricated with various draw-down ratios.

Samples	Outer diameter of MCP (μm)	Inner-diameter of MC (μm)
**MCP-55**	188.1	101.9
**MCP-63**	176.6	101.6
**MCP-67**	165.5	100.7
**MCP-71**	158.6	100.1

Supplementary Table 14 | The tensile strength, Young's modulus, strain, and toughness of the fabricated MC fibers with different weight percentages of CNTs. All error bars show mean ± SD.

Samples	S (μm^2) Whole fiber	Tensile strength (MPa)	Young's modulus (GPa)	Strain (%)	Toughness (MJ m^{-3})
**MX**	5,754.9 ± 73.9	109.3 ± 2.2	21.5 ± 0.1	0.58 ± 0.01	0.36 ± 0.02
**MC-1%**	5,116.8 ± 68.8	284.3 ± 4.7	32.8 ± 0.3	2.56 ± 0.03	4.42 ± 0.20
**MC-2%**	4,968.3 ± 97.7	569.6 ± 3.9	37.2 ± 1.0	5.54 ± 0.09	17.38 ± 1.29
**MC-3%**	5,139.2 ± 104.6	631.8 ± 4.5	42.3 ± 1.1	6.14 ± 0.17	23.19 ± 1.19
**MC-4%**	5,160.8 ± 84.6	502.8 ± 3.5	34.9 ± 1.4	4.45 ± 0.03	11.54 ± 0.43
**MC-5%**	5,530.6 ± 96.8	346.4 ± 6.6	29.1 ± 0.7	3.44 ± 0.07	6.73 ± 0.42

Supplementary Table 15 | The electrical conductivity of the fabricated MC fibers with different weight percentages of CNTs. All error bars show mean ± SD.

Samples	Conductivity (S cm^{-1})
**MX**	8,944.1 ± 85.8
**MC-1%**	10,897.3 ± 40.7
**MC-2%**	11,168.5 ± 36.9
**MC-3%**	11,364.8 ± 65.8
**MC-4%**	10,046.6 ± 54.2
**MC-5%**	9,543.6 ± 46.8

Supplementary Table 16 | The tensile strength, Young's modulus, strain, and toughness of the fabricated MCP fibers with increasing draw-down ratios. All error bars show mean ± SD.

Samples	S (μm^2) Whole fiber	Tensile strength (MPa)	Young's modulus (GPa)	Strain (%)	Toughness (MJ m^{-3})
**PLA**	28,165.5 ± 43.5	93.6 ± 0.3	1.0 ± 0.1	342.92 ± 2.11	256.6 ± 7.5
**MCP-55**	28,015.8 ± 98.5	418.3 ± 5.9	21.9 ± 1.9	49.03 ± 0.86	180.4 ± 6.9
**MCP-63**	21,787.3 ± 104.6	549.2 ± 13.2	31.4 ± 3.0	39.82 ± 0.61	164.8 ± 2.1
**MCP-67**	16,481.2 ± 76.6	683.4 ± 9.0	48.6 ± 3.0	28.75 ± 0.73	153.7 ± 4.9
**MCP-71**	14,654.2 ± 48.7	941.5 ± 5.9	61.43 ± 2.1	22.50 ± 0.07	147.9 ± 4.2

Supplementary Table 17 | The cross-sectional area of the inner MC fibers and electrical conductivity of the fabricated MCP fibers with increasing draw-down ratios. All error bars show mean ± SD.

Samples	S (μm^2) Inner MC fiber	Conductivity (Inner MXene fiber, MCP_{core}) (S cm^{-1})	Conductivity (Whole composite fiber) (S cm^{-1})
**MC-3%**	5,139.2 ± 104.6	11,856.7 ± 39.6	-
**MCP-55**	5,096.2 ± 69.9	9,245.8 ± 54.9	1,681.9 ± 17.9
**MCP-63**	4,976.1 ± 86.5	10,986.8 ± 67.5	2,509.3 ± 23.9
**MCP-67**	4,607.4 ± 124.6	11,498.5 ± 125.7	3,214.5 ± 21.4
**MCP-71**	4,451.2 ± 62.5	12,836.4 ± 108.7	3,899.0 ± 14.8

Supplementary Table 18 | Comparison of the tensile strength, toughness, and electrical conductivity of ultrastrong MCP fiber with that of other reported MXene-based fibers.

Samples	Fabrication method	Tensile strength (MPa)	Toughness (MJ m^{-3})	Conductivity (S cm^{-1})	Density (g cm^{-3})	Specific strength (MPa cm^3 g^{-1})	Encapsulation layer	Ref. No.
**LC MXene fiber**	Wet spinning	40.5	~0.4	7,750	~3.6	~11.25	No	1
**Pure MXene**	Wet spinning	343.68	~0.5	12,503.78	~3.74	~91.89	No	2
**MXene/Cellulose**	Wet spinning	136.5	~3.4	4.8	-	-	No	3
**cPM**	Wet spinning	502.3	59.7	1,115.9	-	-	No	4
**Pure $Ti_3C_2T_x$**	Wet spinning	118	~1.0	7,200	-	-	No	5
**Pure MXene Fiber**	Wet spinning	63.9	~0.1	7,713	-	-	No	6
**MXene/Graphene**	Wet spinning	12.9	~0.2	290	-	-	No	7
**Kevlar/$Ti_3C_2T_x$**	Wet spinning	18	~0.3	0.01128	-	-	No	8
**MXene/rGO**	Wet spinning	132.5	~0.3	72.3	-	-	No	9
**MXene/ANF**	Wet spinning	160	~12.07	26.31	-	-	Yes	10
**ANF/MXene**	Wet spinning	~502.9	~48.1	~3,000	2.6	~193.4	Yes	11
**MGP**	Wet spinning	565.2	19.2	8,110.4	~3.68	~153.6	No	12
**$Ti_3C_2T_x$/ANF**	Wet spinning	104	~4.1	1,025	-	-	No	13
**MXene/PEDOT:PSS**	Wet spinning	58.1	~0.3	1,489.8	-	-	No	14
**rGO/MXene**	Wet spinning	110.7	3.8	743.1	-	-	No	15
**MXene/PU**	Wet spinning	7.8	-	1	-	-	No	16
**MXene Aerogel**	Wet spinning	~1.0	~0.01	117.09	~0.035	~28.6	No	17
**MXene/CNTs/HA**	Wet spinning	~56.67	-	-	-	-	No	18
**M-CMC**	Wet spinning	~81	~0.89	~1,073	-	-	No	19
**MKB**	Wet spinning	~242.6	~1.0	~1,412	2.5	~97.0	No	20

**MXene-silk**	Wet spinning	~70	~0.7	~3,700	-	-	No	21
**CNT@MXene fiber**	Wet spinning	~10.1	-	2.22	-	-	Yes	22
**MLMS**	Dry spinning	~18.2	-	~0.748	-	-	No	23
**FM**	Dry spinning	64	0.108	2,295	-	-	No	24
**MXene/CNT yarns**	Scrolling	~27	~2.03	26	-	-	No	25
**MXene/CNT**	Scrolling	38.4	~1.0	2.7	-	-	No	26
**MXene/PEDOT:PSS-coated carbon fiber**	Coating	~3000	-	~198	-	-	No	27
**MXene-coated cotton yarn**	Coating	460	~24.15	199	-	-	No	28
**MGP-T**	Wet spinning/ Thermal drawing	585.5	66.7	8,802.4	-	-	Yes	12
**CCM**	Thermal drawing	707.73	125.12	11,959.4	-	-	Yes	29
**MC**	**Wet spinning**	**~631.8**	**~23.2**	**~11,856.7**	**~3.90**	**~162.0**	**No**	**This work**
**MCP**	**Wet spinning/ Thermal drawing**	**~941.5**	**~147.9**	**~12,836.4 (For inner MXene fiber, MCP_{core}) ~3899.0 (For whole composite fiber)**	**~1.97**	**~477.9**	**Yes**	**This work**

Supplementary Table 19 | Comparison of the tensile strength, toughness, and electrical conductivity of the MXene composite fiber with that of other reported fibers.

Fibers	Tensile strength (MPa)	Toughness (MJ m^{-3})	Conductivity (S cm^{-1})	Diameter (μm)	Encapsulation layer	Ref. No.
**rGG-Ca^{2+}**	501.5	16.8	410	~6	No	30
**rGO-NaOH**	140.0	3.9	250	~100	No	31
**rGO-Ag-NW**	300.0	7.8	930	~10	No	32
**Porous rGO fiber**	50.0	0.3	25.1	~50	No	33
**Writing rGO**	365.0	5.6	270	~150	No	34
**LGO-SA**	784.9	6.8	35.8	~8.4	No	35
**Dry film scrolled**	39.2	0.3	416	~229	No	36
**rGO-NaOH**	~183	1.6	2.21	-	No	37
**rGO-CTAB**	182	~3.2	35.0	~47	No	38
**CRG-PVA**	~199	~3.6	-	~50	No	39
**rGO-HPG**	~165.0	~1.2	~0.049	~50	No	40
**rGO-NaDC**	238.0	~2.4	308	~40	No	41
**rGO-Ca^{2+}-PCDO**	842.6	15.8	292.4	~21	No	42
**RGG-HPG-HI**	487.0	9.5	~52.6	~10	No	43
**BGNF**	740.1	18.7	384.3	~30	No	44
**rGO-CS-Ca^{2+}**	743.6	26.3	179.0	~20	No	45
**Graphene fiber**	~1,080	~8	1,100	~10	No	46
**GF**	1,450	~5.4	8,000	~1.6	No	47
**Concentric graphene fiber**	~2,000	~2.2	12,100	~430	No	48

**Carbon fiber, AS4, Hexcel**	~4,270	-	650	-	No	49
**Carbon fiber, T300, Cytec**	~3,750	-	556	-	No	49
**Carbon fiber**	~4,000	~25	-	-	No	50
**Kevlar fiber**	~3,600	~50	-	-	-	50
**Nylon fiber**	950	~80	-	-	-	50
**CNT ribbon**	~5,200	-	~5,000	~20	No	51
**CNT fiber**	8,800	-	-	-	No	52
**CNT fiber**	~1,004	~17	~772	~7.3	No	53
**Py-PDA-CNT fiber**	~4,035	~121	~4,856	-	No	53
**CNT fiber**	1,900	~61.8	-	~10	No	54
**S-DWNT fiber**	6,570	~58	22,200	~30	No	25
**SWCNT strands fiber**	~1,000	~40	-	~300	No	56
**SACNT/PVA yarns fiber**	~2,000	~14	~920	~15	No	57
**Liquid metal fiber**	~28	~12	~34,600	~305	Yes	58
**Aluminum (Al) wire**	~283.5	~19.3	270,000	-	No	11
**Silver (Ag) wire**	~230.6	~14.9	590,000	-	No	11
**Silver-coated nylon fiber**	~280	-	-	~226	No	59
**Silver nanowire coated nylon threads**	-	-	~0.8 Ω cm^{-1}	~700	No	60
**Silver coated staple polyamide fiber**	~33 cN tex^{-1}	-	~1030 Ω	~16	No	61
**AgNWs/SWCNTs-coated Nylon-6 fiber**	-	-	~1665	~38	No	62
**Ag-coated Kevlar fiber**	~44 N	-	0.38 Ω cm^{-1}	~20	NO	63
**Ag-coated Kevlar fiber**	16.06 cN	-	0.53 Ω cm^{-1}	~15	No	64

**Ag-coated Kevlar fiber**	-	-	0.89 Ω cm^{-1}	~13	No	65
**Ag-coated Kevlar fiber**	44 N	-	0.2 Ω cm^{-1}	~15	No	66
**Ag-coated Kevlar fiber**	-	-	0.61 mΩ cm^{-1}	~16	No	67
**Silver-plated Kevlar fiber**	18.89 cN dT^{-1}	-	0.067 Ω cm^{-1}	~15	No	68
**Ag-plated Kevlar fiber**	17.48 cN dT^{-1}	-	0.043 Ω cm^{-1}	-	No	69
**Silver-coated PPTA**	-	-	~0.23 Ω cm^{-1}	~16.94	No	70
**Cu wire**	**~260.3**	**~41.53**	**580,000**	**~100**	**No**	**This work**
**E-Cu wire**	**~177.0**	**~65.74**	**580,000**	**~160**	**Yes**	**This work**
**MC-180**	**~1,056.0**	**~45.9**	**~14,589.5**	**~33.8**	**No**	**This work**
**MCP**	**~941.5**	**~147.9**	**~12,836.4 (For inner MXene fiber, MCP_{core}) ~3899.0 (For whole composite fiber)**	**~158.6**	**Yes**	**This work**
**MCP-V**	**~1,088.5**	**~293.5**	**~13,567.4 (For inner MXene fiber) ~395.5 (For whole composite fiber)**	**~430.6**	**Yes**	**This work**

Supplementary Table 20 | The electrical conductivity retention rates under different loading-unloading cyclic testing conditions for MXene composite fibers.

Loading stress (MPa)	Samples	Number of cycles	Electrical conductivity retention rate (%)
**100**	MX	200	0 (Break)
**500**	MC-3%	4000	68.4
**250**	MCP-55	4000	52.3
	MCP-63	4000	69.7
	MCP-67	4000	84.4
	MCP-71	4000	98.8
**400**	MCP-71	4000	92.4
**500**	MCP-71	4000	85.6
**600**	MCP-71	4000	60.9

Supplementary Table 21 | Comparison of the tensile strength and electrical resistance change under mechanical bending of MCP and MCP-V fibers with those of reported conductive materials for wireless applications.

Sample	Type	Dimension (μm)	Tensile strength (MPa)	Tested cycles	Resistance relative change (%)	Ref.
**MCP-V fiber**	**Textile**	**~430.6 (diameter)**	**~1,088.5**	**~90,000**	**~0.2**	**This work**
**MCP fiber**	**Textile**	**~158.6 (diameter)**	**~941.5**	**~90,000**	**~0.3**	**This work**
**Copper wire**	**Textile**	**~100 (diameter)**	**~260.3**	**~300**	**break**	**This work**
**E-Cu**	**Textile**	**~130 (diameter)**	**~177.0**	**~1450**	**break**	**This work**
**Liquid metal fiber**	Textile	305 (diameter)	~28	24,000	<1	58
**Liquid metal**	PDMS	289 (thickness)	-	5,000	<1	71
**Liquid metal (LM)**	PVA	150 (thickness)	-	10,000	<1	72
**Conductive thread**	Textile	370 (diameter)	~60	10,000	<1	58
**Elektrisola E-threads**	Textile	120 (diameter)	-	300	-	73
**MXene**	PET	5.5 (thickness)	-	5,000	<1	74
**MXene**	PDMS/PI	~2 (thickness)	-	1,000	5	75
**Graphene**	PEN	~50 (thickness)	-	11,000	<3	76
**Graphene**	A4 Paper	~7.8 (thickness)	-	2,000	5	77
**Silver conductive ink**	Textile	~75 (thickness)	-	1,000	5-10	78
**Copper paste**	PET	~110 (thickness)	-	500	~760%	79

Supplementary Table 22 | The orientation factor, porosity, and specific strength of the fabricated MCP-V fiber. All error bars show mean ± SD.

Sample	Density (g cm^{-3})	Porosity (Inner MXene fiber) (%)	Orientation factor (*f*)	Specific strength (MPa cm^3 g^{-1})
**MCP-V**	1.75 ± 0.15	4.1 ± 0.1	0.951	622.0 ± 5.8

Supplementary Table 23 | The tensile strength, toughness, and conductivity of the fabricated MCP-V fiber. All error bars show mean ± SD.

Sample	Tensile strength (MPa)	Strain (%)	Toughness (MJ m^{-3})	Conductivity (S cm^{-1})
**MCP-V**	1,088.5 ± 10.1	33.9 ± 0.33	293.5 ± 10.5	13,567.4 ± 210.5 (For inner MXene fiber) 395.5 ± 10.2 (For whole composite fiber)

Supplementary References

1. Zhang, J. et al. Additive-free MXene liquid crystals and fibers. *ACS Cent. Sci.* **6**, 254-265 (2020).

2. Shin, H. et al. Highly electroconductive and mechanically strong $Ti_3C_2T_x$ MXene fibers using a deformable MXene gel. *ACS Nano* **15**, 3320-3329 (2021).

3. Cao, W. et al. MXene-reinforced cellulose nanofibril inks for 3D-printed smart fibres and textiles. *Adv. Funct. Mater.* **29**, 1905898 (2019).

4. Gu, J. et al. Extremely Robust and Multifunctional Nanocomposite Fibers for Strain-Unperturbed Textile Electronics. *Adv. Mater.* **35**, 2209527 (2023).

5. Li. S. et al. Assembly of nanofluidic MXene fibers with enhanced ionic transport and capacitive charge storage by flake orientation. *ACS Nano* **15**, 7821-7832 (2021).

6. Eom, W. et al. Large-scale wet-spinning of highly electroconductive MXene fibers. *Nat. Commun.* **11**, 2825 (2020).

7. Yang, Q. et al. MXene/graphene hybrid fibers for high performance flexible supercapacitors. *J. Mater. Chem. A* **5**, 22113-22119 (2017).

8. Cheng, B., Wu, P. Scalable fabrication of Kevlar/$Ti_3C_2T_x$ MXene intelligent wearable fabrics with multiple sensory capabilities. *ACS Nano* **15**, 8676-8685 (2021).

9. Seyedin, S., Yanza, R., Razal, J. Knittable energy storing fiber with high volumetric performance made from predominantly MXene nanosheets. *J. Mater. Chem. A* **5**, 24076-24082 (2017).

10. Wang, L., Zhang, M., Yang, B., Tan, J. Lightweight, robust, conductive composite fibers based on MXene@aramid nanofibers as sensors for smart fabrics. *ACS Appl. Mater. Interfaces* **13**, 41933-41945 (2021).

11. Liu, L. et al. Super-tough and environmentally stable aramid nanofiber@MXene coaxial fibers with outstanding electromagnetic interference shielding efficiency. *Nano-Micro Lett.* **14**, 111 (2022).

12. Zhou, T. et al. Ultra-compact MXene fibers by continuous and controllable synergy of interfacial interactions and thermal drawing-induced stresses. *Nat. Commun.* **13**, 4564 (2022).

13. Liu, Q. et al. Full-temperature all-solid-state $Ti_3C_2T_x$/aramid fiber supercapacitor with optimal balance of capacitive performance and flexibility. *Adv. Funct. Mater.* **31**, 2010944 (2021).

14. Zhang, J. et al. Highly conductive $Ti_3C_2T_x$ MXene hybrid fibers for flexible and elastic fiber-shaped supercapacitors. *Small* **15**, 1804732 (2019).

15. He, N. et al. Effects of electrolyte mediation and MXene size in fiber-shaped supercapacitors. *ACS Appl. Energy Mater.* **3**, 2949-2958 (2020).
16. Seyedin, S. et al. MXene composite and coaxial fibers with high stretchability and conductivity for wearable strain sensing textiles. *Adv. Funct. Mater.* **30**, 1910504 (2020).
17. Li, Y., Zhang, X. Electrically conductive, optically responsive, and highly orientated $Ti_3C_2T_x$ MXene aerogel fibers. *Adv. Funct. Mater.* **32**, 2107767 (2022).
18. Zheng, T. et al. Wet-spinning of continuous hyaluronic-based MXene/CNTs hybrid fibers for flexible supercapacitor applications. *Mater. Letters* **336**, 133891 (2023).
19. Wang, H. et al. Nacre-Inspired Strong MXene/Cellulose Fiber with Superior Supercapacitive Performance via Synergizing the Interfacial Bonding and Interlayer Spacing. *Nano Lett.* **23**, 5663-5672 (2023).
20. Dai, H. et al. Bio-Inspired Interfacial Engineering of MXene Fibers Toward Synergistic Improvement in Mechanical Strength and Electrochemical Performance. *Adv. Funct. Mater.* **34**, 2312654 (2024).
21. Usman, K. et al. Robust Biocompatible Fibers from Silk Fibroin Coated MXene Sheets. *Adv. Mater. Interfaces* **10**, 2201634 (2023).
22. Feng, W. et al. Core-Sheath CNT@MXene Fibers Toward Absorption-Dominated Electromagnetic Interference Shielding Fabrics. *Adv. Fiber Mater.* **6**, 1657-1668 (2024).
23. Yi, P. et al. MXene-Reinforced Liquid Metal/Polymer Fibers via Interface Engineering for Wearable Multifunctional Textiles. *ACS Nano* **16**, 14490-14502 (2022).
24. Xia, Z. et al. Rheology Engineering for Dry-Spinning Robust N-Doped MXene Sediment Fibers toward Efficient Charge Storage. *Small* **19**, 2304687 (2023).
25. Wang, Z. et al. High-performance biscrolled MXene/carbon nanotube yarn supercapacitors. *Small* **14**, 1802225 (2018).
26. Yu, C. et al. A solid-state fibriform supercapacitor boosted by host-guest hybridization between the carbon nanotube scaffold and MXene nanosheets. *Small* **14**, 1801203 (2018).
27. Zhang, J. et al. MXene: a potential candidate for yarn supercapacitors. *Nanoscale* **9**, 18604-18608 (2017).
28. Uzun, S. et al. Knittable and Washable Multifunctional MXene-Coated Cellulose Yarns. *Adv. Funct. Mater.* **29**, 1905015 (2019).
29. Zhou, T. et al. Interlocking-Governed Ultra-Strong and Highly Conductive MXene Fibers Through Fluidics-Assisted Thermal Drawing. *Adv. Mater.* **35**, 2305807 (2023).

30. Xu, Z., Sun, H., Zhao, X., Gao, C. Ultrastrong fibers assembled from giant graphene oxide sheets. *Adv. Mater.* **25**, 188-193 (2013).
31. Xu, Z., Gao, C. Graphene chiral liquid crystals and macroscopic assembled fibres. *Nat. Commun.* **2**, 571 (2011).
32. Xu, Z., Liu, Z., Sun, H., Gao, C. Highly electrically conductive Ag-doped graphene fibers as stretchable conductors. *Adv. Mater.* **25**, 3249-3253 (2013).
33. Aboutalebi, S. H. et al. High-performance multifunctional graphene yarns: toward wearable all-carbon energy storage textiles. *ACS Nano* **8**, 2456-2466 (2014).
34. Cao, J. et al. Programmable writing of graphene oxide/reduced graphene oxide fibers for sensible networks with in situ welded junctions. *ACS Nano* **8**, 4325-4333 (2014).
35. Hu, X. et al. A novel wet-spinning method of manufacturing continuous bio-inspired composites based on graphene oxide and sodium alginate. *Nano Research* **9**, 735-744 (2016).
36. Cruz-Silva, R. et al. Super-stretchable graphene oxide macroscopic fibers with outstanding knotability fabricated by dry film scrolling. *ACS Nano* **8**, 5959-5967 (2014).
37. Jalili, R. et al. Scalable one-step wet-spinning of graphene fibers and yarns from liquid crystalline dispersions of graphene oxide: towards multifunctional textiles. *Adv. Funct. Mater.* **23**, 5345-5354 (2013).
38. Cong, H., Ren, X., Wang, P., Yu, S. Wet-spinning assembly of continuous, neat, and macroscopic graphene fibers. *Sci. Rep.* **2**, 613 (2012).
39. Kou, L., Gao, C. Bioinspired design and macroscopic assembly of poly(vinyl alcohol)-coated graphene into kilometers-long fibers. *Nanoscale* **5**, 4370-4378 (2013).
40. Hu, X., Xu, Z., Gao, C. Multifunctional, supramolecular, continuous artificial nacre fibres. *Sci. Rep.* **2**, 767 (2012).
41. Huang, G. et al. Highly strong and elastic graphene fibres prepared from universal graphene oxide precursors. *Sci. Rep.* **4**, 4248 (2014).
42. Zhang, Y. et al. Ultrastrong bioinspired graphene-based fibers via synergistic toughening. *Adv. Mater.* **28**, 2834-2839 (2016).
43. Hu, X., Xu, Z., Liu, Z., Gao, C. Liquid crystal self-templating approach to ultrastrong and tough biomimic composites. *Sci. Rep.* **3**, 2374 (2013).
44. Zhang, Y. et al. Bioinspired supertough graphene fiber through sequential interfacial interactions. *ACS Nano* **12**, 8901-8908 (2018).
45. Wang, X. et al. Ultratough bioinspired graphene fiber via sequential toughening of hydrogen and ionic bonding. *ACS Nano* **12**, 12638-12645 (2018).

46. Xin, G. et al. Highly thermally conductive and mechanically strong graphene fibers. *Science* **349**, 1083-1087 (2015).
47. Xu, Z. et al. Ultrastiff and strong graphene fibers via full-scale synergetic defect engineering. *Adv. Mater.* **28**, 6449-6456 (2016).
48. Li, P. et al. Bidirectionally promoting assembly order for ultrastiff and highly thermally conductive graphene fibres. *Nat. Commun.* **15**, 409 (2024).
49. Liu, Y., Kumar, S. Recent Progress in Fabrication, Structure, and Properties of Carbon Fibers. *Polym. Rev.* **52**, 234-258 (2012).
50. Omenetto, F. G., Kaplan, D. L New Opportunities for an Ancient Material. *Science* **329**, 528-531 (2010).
51. Tran, T. et al. Super-strong and highly conductive carbon nanotube ribbons from post-treatment methods. *Carbon* **99**, 407-415 (2016).
52. Koziol, K. et al. Windle, High-Performance Carbon Nanotube Fiber. *Science* **318**, 1892-1895 (2007).
53. Ryu, S. et al. Direct Insulation-to-Conduction Transformation of Adhesive Catecholamine for Simultaneous Increases of Electrical Conductivity and Mechanical Strength of CNT Fibers. *Adv. Mater.* **27**, 3250-3255 (2015).
54. Zhang, X. et al, Ultrastrong, Stiff, and Lightweight Carbon-Nanotube Fibers. *Adv. Mater.* **19**, 4198-4201 (2007).
55. Lee, D. et al. Ultrahigh strength, modulus, and conductivity of graphitic fibers by macromolecular coalescence. *Sci. Adv.* **8**, eabn0939 (2022).
56. Zhu, H. W. et al. Direct Synthesis of Long Single-Walled Carbon Nanotube Strands. *Science* **296**, 884-886 (2002).
57. Liu, K. et al. Scratch-Resistant, Highly Conductive,and High-Strength Carbon Nanotube-Based Composite Yarns. *ACS Nano* **4**, 5827-5834 (2010).
58. Lin, R. et al. Digitally-embroidered liquid metal electronic textiles for wearable wireless systems. *Nat. Commun.* **13**, 2190-2199 (2022)
59. Qureshi, Y., Tarfaoui, M., Lafdi, K. K., Lafdi, K. Real-time strain monitoring performance of flexible Nylon/Ag conductive fiber. *Sens. Actuators A: Phys.* **295**, 612-622 (2019).
60. Atwa, Y., Maheshwari, N., Goldthorpe, I. A. Silver nanowire coated threads for electrically conductive textiles. *J. Mater. Chem. C* **3**, 3908-3912 (2015).

61. Ozen, M. S., Sancak, E., Soin, N., Shah, T. H., Siores, E. Investigation of electromagnetic shielding effectiveness of needle punched nonwoven fabric produced from conductive silver coated staple polyamide fibre. *J. Text. Inst.* **107**, 912-922 (2016).
62. Yang, Z. et al. Strain-Durable High-Conductivity Nylon-6 Fiber with 1D Nanomaterial Lamellar Cladding for Massive Production. *ACS Appl. Mater. Interfaces* **13**, 57759-57767 (2021).
63. Yua, D., Mu, S., Liu, L., Wang, W. Preparation of electroless silver plating on aramid fiber with good conductivity and adhesion strength. *Colloids Surf. A: Physicochem. Eng. Asp.* **483**, 53-59 (2015).
64. Sun, B. et al. Preparation of silver nanoparticle functionalized aramid fiber by employing dopamine and silane coupling agent modification. *J. Appl. Polym. Sci.* **139**, e53190 (2022).
65. Sun, Z. et al. Preparation of Silver-Plated Para-Aramid Fiber by Employing Low-Temperature Oxygen Plasma Treatment and Dopamine Functionalization. *Coatings* **9**, 599 (2019).
66. Zhang, H. et al. Development of Electroless Silver Plating on Para-Aramid Fibers and Growth Morphology of Silver Deposits. *J Appl Polym Sci.* **124**, 3363-3371 (2011).
67. Wang, W. et al. Surface Silverized Meta-Aramid Fibers Prepared by Bio-inspired Poly(dopamine) Functionalization. *ACS Appl. Mater. Interfaces* **5**, 2062-2069 (2013).
68. Geng, X. et al. Conductive Aramid Fibers from Electroless Silver Plating of Crosslinked HPAMAM-Modified PPTA: Preparation and Properties. *ACS Omega* **7**, 17014-17023 (2022).
69. Geng, S. et al. The strategic use of hyperbranched polyamidoamine through chemical bonding in fabricating stable, highly conductive Ag-plated aramid fiber. *Eur. Polym. J.* **198**, 112402 (2023).
70. Onggar, T., Amrhein, G., Abdkader, A. R., Hund, R., Cherif, C. Wet-chemical method for the metallization of a para-aramid filament yarn wound on a cylindrical dyeing package. *Text. Res. J.* **87**, 1192-1202 (2017).
71. Yang, J. et al. Defect-free, high resolution patterning of liquid metals using reversibly sealed, reusable polydimethylsiloxane microchannels for flexible electronic applications. *J. Mater. Chem. C* **5**, 6790-6797 (2017).
72. Teng, L. et al. Liquid metal-based transient circuits for flexible and recyclable electronics. *Adv. Funct. Mater.* **29**, 1808739 (2019).

73. Zhong, J., Kiourti, A., Sebastian, T., Bayram, Y., Volakis, J. L. Conformal load-bearing spiral antenna on conductive textile threads. *IEEE Antennas Wireless Propag. Lett.* **16**, 230-233 (2017).

74. Han, M. et al. Solution-processed $Ti_3C_2T_x$ MXene antennas for radio-frequency communication. *Adv. Mater.* **33**, 2003225 (2021).

75. Shao, Y. et al. Room-temperature high-precision printing of flexible wireless electronics based on MXene inks. *Nat. Commun.* **13**, 3223 (2022).

76. Scidà, A. et al. Application of graphene-based flexible antennas in consumer electronic devices. *Mater. Today* **21**, 223-230 (2018).

77. Pan, K. et al. Sustainable production of highly conductive multilayer graphene ink for wireless connectivity and Iot applications. *Nat. Commun.* **9**, 5197 (2018).

78. Lee, J. H., Dzagbletey, P. A., Jang, M., Chung, J., So, J. Flat yarn fabric substrates for screen-printed conductive textiles. *Adv. Eng. Mater.* **22**, 2000722 (2020).

79. Shin, K., Lee, J. S., Hong, J., Jang, J. One-step fabrication of a highly conductive and durable copper paste and its flexible dipole tag-antenna application. *Chem. Commun.* **50**, 3093-3096 (2014).
