# Supplementary material for: Ultrastrong MXene composite fibers through static-dynamic densification for wireless electronic textiles
Source: Nat Commun. 2025 Dec 9;16:10968. doi: 10.1038/s41467-025-65931-5 (PMC12689709; doi:10.1038/s41467-025-65931-5)
Supplement: Supplementary file 2 — Description of Additional Supplementary Information [file 41467_2025_65931_MOESM2_ESM.pdf]

Description of Additional Supplementary Files

File Name: Supplementary Movie 1
Description: MC fiber continuously fabricated by wet spinning.

File Name: Supplementary Movie 2
Description: MCP fiber fabricated via thermal drawing.

File Name: Supplementary Movie 3
Description: MCP fiber subjected to repeated loading of 1.5 kg.

File Name: Supplementary Movie 4
Description: Slice section images from nano-CT for MX fiber.

File Name: Supplementary Movie 5
Description: Slice section images from nano-CT for MCP fiber.

File Name: Supplementary Movie 6
Description: Fracture mechanism of MCP fiber.

File Name: Supplementary Movie 7
Description: Spiral flower design using MCP fiber by digital embroidery process.

File Name: Supplementary Movie 8
Description: Wireless power unit based on MCP textile.

File Name: Supplementary Movie 9
Description: Wireless power unit can consistently power the LED light even after multiple cycles of bending, stretching, and poking.

File Name: Supplementary Movie 10
Description: Battery-free wireless sensing unit based on MCP textile.

File Name: Supplementary Movie 11
Description: Wireless storage unit based on smart textile.

File Name: Supplementary Movie 12
Description: Storage unit based on MCP textile work normally in water.

File Name: Supplementary Movie 13
Description: The MCP-V fiber being wirelessly powered by the hand where it captures and couples with the surrounding electromagnetic energy.

File Name: Supplementary Movie 14
Description: MCP-V fibers can be sewn onto textile and maintain their luminous state.

File Name: Supplementary Movie 15

Description: The dyed MCP-V textile emit a red glow when exposed to bodycouple EM fields after multiple cycles of bending, stretching, and poking.

File Name: Supplementary Movie 16
Description: The MCP-V textile is capable of controlling drone flight.
